# Respiratory syncytial virus disease burden in children and adults from Latin America: a systematic review and meta-analysis

**DOI:** 10.3389/fpubh.2024.1377968

**Published:** 2024-10-16

**Authors:** Agustín Ciapponi, María Carolina Palermo, María Macarena Sandoval, Elsa Baumeister, Silvina Ruvinsky, Rolando Ulloa-Gutierrez, Katharina Stegelmann, Sofía Ardiles Ruesjas, Joaquín Cantos, Jorge LaRotta, Rodrigo Sini de Almeida, Ariel Bardach

**Affiliations:** ^1^Instituto de Efectividad Clínica y Sanitaria (IECS-CONICET), Buenos Aires, Argentina; ^2^Centro de Investigaciones Epidemiológicas y Salud Pública (CIESP-IECS), CONICET, Emilio Ravignani 2024 (C1414CPV), Buenos Aires, Argentina; ^3^National Influenza Centre PAHO/WHO, Servicio Virosis Respiratorias, Departamento Virología, Instituto Nacional de Enfermedades Infecciosas, Buenos Aires, Argentina; ^4^Hospital Nacional de Pediatría, ‘Dr. Juan P. Garrahan’, Buenos Aires, Argentina; ^5^Servicio de Aislamiento, Hospital Nacional de Niños "Dr. Carlos Sáenz Herrera", Caja Costarricense de Seguro Social (CCSS), San José, Costa Rica; ^6^Facultad de Medicina, Universidad de Ciencias Médicas (UCIMED), San José, Costa Rica; ^7^Instituto de Investigación en Ciencias Médicas (IICIMED), San José, Costa Rica; ^8^Barcelona Institute for Global Health, Hospital Clínic-Universitat de Barcelona, Barcelona, Spain; ^9^Vaccine Medical, Pfizer SAS, Bogotá, Colombia; ^10^Pfizer Inc, New York, NY, United States

**Keywords:** respiratory syncytial virus, bronchiolitis, lower respiratory tract infection, prevalence, incidence, mortality, seasonality, Latin America and the Caribbean

## Abstract

**Background:**

Respiratory Syncytial Virus (RSV) is a common cause of lower respiratory tract infections (LRTI) and hospitalization worldwide. The impact of RSV in Latin America and the Caribbean (LAC) including expensive treatment options, such as palivizumab, have been extensively discussed. However, publications on the impact of RSV disease burden in the region are scarce. This systematic review aimed to determine the incidence and prevalence of RSV in LAC by age and RSV subtype.

**Methods:**

We conducted a systematic review following Cochrane methods to evaluate the disease burden of RSV in LAC countries. We searched studies from January 2012 to January 2023 in literature databases and grey literature without language restrictions. We included guidelines, observational, economic, and surveillance studies from LAC countries. Pairs of reviewers independently selected, and extracted data from included studies. The risk of bias was assessed using the Study Quality Assessment Tools (NHLBI) and AGREE-II. We performed proportion meta-analyses using methods to stabilize the variance. The protocol was registered in PROSPERO (CRD42023393731).

**Results:**

We included 156 studies, mainly from Brazil (25%), Colombia (14.5%), and Argentina (13.8%), as well as four clinical practice guidelines. Most studies were cross-sectional (76.9%) and were classified as low risk of bias (52.6%). The majority included inpatients (85.6%), pediatric (73.7%), and normal-risk patients (67.1%). The highest pooled prevalence was estimated in patients <1 year old (58%), with type A and B prevalence of 52 and 34%, respectively. The RSV-LRTI incidence was 15/100 symptomatic infants aged <2 years old, and the ICU admission was 42%. The RSV-LRTI lethality was 0.6, 3% in patients aged <2 and 0–5 years old, respectively, and 23% among >65 years old high-risk patients. The identified guidelines lack methodological rigor and have limitations in their applicability. The seasonality was more evident in South America than in Central America and The Caribbean, with a clear gap during the pandemic.

**Conclusion:**

This is the most exhaustive and updated body of evidence describing a significant burden of RSV in LAC, particularly at the extremes of life, and its seasonality patterns. Our findings could contribute could contribute facilitating effective prevention and treatment strategies for this significant public health problem.

**Systematic review registration:**

PROSPERO CRD UK (registration number: CRD42023393731).

## 1 Introduction

Respiratory syncytial virus (RSV) is a leading cause of acute lower respiratory tract infections (LRTIs), among children, old people, and those with underlying comorbidities (1, 2). RSV is the most common cause of viral pneumonia and bronchiolitis in infants, causing 28% of all cases of LRTI, and 13–22% of related mortality in children aged 0–59 months, resulting in a considerable disease burden worldwide (3). Hospitalized infants with RSV bronchiolitis and pneumonia represent a higher healthcare use of resources (4). These include frequent admission to the Pediatric Intensive Care Unit (PICU) and the use of non-invasive respiratory support or mechanical ventilation. While risk factors like prematurity, lower age, and comorbidities such as congenital heart disease (CHD) and chronic lung disease contribute to RSV hospitalization, it is noteworthy that the majority of hospitalized infants are without comorbidities, highlighting the widespread impact of the virus on otherwise healthy infants (5, 6).

In adults, principally individuals aged 65 and above in high-income countries and older than 60 years old in lower-income countries, RSV is an important cause of acute respiratory infection (ARI), hospitalization, and death (7). Among US adults, an estimated 177,000 hospitalizations and 14,000 deaths are associated with RSV infections annually (8, 9).

RSV infection generates a substantial economic burden in the infant and adult populations on a global scale. To analyze the potential benefits of introducing preventive and therapeutic interventions, it is essential to have estimates of the RSV disease epidemiology and economic burdens in Latin America and the Caribbean (LAC). Previous systematic reviews highlighted the evidence gaps about the burden of RSV in Latin America (10, 11). Additionally, data are scarce by age groups, RSV subtype, coinfection, or population risk, and rarely considered seasonality aspects in children and adult patients (≥ 18 years) in the LAC region. A rigorous systematic review is needed to cover these gaps about the disease burden of RSV in LAC.

The objective of this systematic review and meta-analysis was primarily to determine the incidence and prevalence of RSV in LAC. Also, to determine RSV severity and complications in subgroups of interest, the seasonality and RSV outbreaks, and describe the results over time,.

## 2 Methods

We present here part of a broader systematic review that also included the use of resources and direct/indirect costs associated with RSV disease in LAC. The economic findings were published elsewhere in another publication (63). We conducted a systematic literature review and meta-analysis following the Cochrane Manual of Systematic Reviews (12), the PRISMA (13, 14) statement for reporting systematic reviews and meta-analyses, and the MOOSE guidelines (specific for reviews of observational studies) (15). The protocol was registered in PROSPERO (registration number: CRD42023393731).

### 2.1 Inclusion criteria

Studies of any epidemiological design in Spanish, English, or Portuguese and surveillance reports published since January 1st, 2012, and January 4th, 2023, which included LAC patients of any age or risk group, were eligible for inclusion. We considered that previously published studies could make it difficult to estimate the current burden of RSV. We planned to include observational studies such as cohorts, case–control studies, and representative case series (involving at least 50 laboratory-confirmed RSV cases or at least 10 RSV patients with complications), the control arms of the randomized clinical trials (RCTs) and quasi-RCTs, controlled before-and-after studies (CBAs) and uncontrolled before-and-after studies (UBAs), interrupted time series (ITSs), controlled ITSs (CITSs) that meet the inclusion criteria of the Cochrane COPD group (16). We also included clinical practice guidelines and consensus documents that addressed immunoprophylaxis. We considered systematic reviews and meta-analyses only as sources of primary studies.

### 2.2 Search strategy for identification of studies and data sources

We searched records published during the study period in the following databases: PubMed, LILACS, Embase, CINAHL, Cochrane Library, Web of Science, Tufts Economic Database, and EconLIT. The detailed search strategy is described in the Supplementary material. For studies with multiple publications, the one with the largest sample size and/or the most recent publication was considered the primary reference; secondary references were used to complement the data. Reference lists of included articles were hand-searched for additional information. If necessary, authors of relevant articles were consulted for missing or clarifying information. The search for gray literature was performed in the following sources: databases of proceedings of regional and international congresses, websites of major medical regional and international societies and associations related to the topic, and the Virtual Health Library. We also contacted members of the PAHO, who gave us access to the database of RSV cases reported from LAC countries between 2017 and 2023. Generic internet search and metasearch engines (Google) were also searched. The complete search strategy is detailed in the Supplementary material.

### 2.3 Selection of articles and data extraction

Selection, data extraction, and risk of bias assessment of each article were performed independently by pairs of reviewers, and discrepancies were resolved by consensus of the entire team. All phases of study selection were carried out using COVIDENCE^®^, a web-based platform designed for the systematic review process (17) and data extraction used a previously piloted data extraction form (based on five studies). From eligible articles, the research team extracted the following study information: publication and study characteristics (type of publication, year published, authors, geographic location, study design including domains for risk of bias assessment), study population characteristics (age, sex, sample size, population risk, inclusion, and exclusion criteria), and outcomes [incidence, prevalence, case fatality rate, hospitalization rate, intensive care unit (ICU) admission rate, disease complications of RSV, and RSV seasonality and outbreaks].

### 2.4 Risk of bias assessment

We evaluated the risk of bias of the included studies using a checklist developed by the U.S. National Heart, Lung, and Blood Institute, which classifies studies as high risk of bias (poor), uncertain (fair), and low risk of bias (good) (18). For cohort studies and cross-sectional studies assessment, the tool comprises 14 items, while case series studies are assessed based on nine items. The clinical practice guidelines were assessed by the AGREE-II instrument (19). The AGREE-II instrument consists of 23 items, grouped into six domains: (1) Scope and objectives, (2) Participation of decision-makers, (3) Methodological rigor, (4) Clarity of presentation, (5) Applicability, and (6) Editorial independence. A narrative and tabular synthesis were conducted of the available recommendations from clinical practice guidelines and consensus issued by scientific societies and health authorities at the national level.

### 2.5 Statistical analysis

We reported narrative and structured information alongside descriptive statistics to characterize results. To analyze our data, we conducted proportion meta-analyses using the R software (meta-package) for all analyses. The pooled proportion was calculated as the back-transformation of the weighted mean of the transformed proportions, using inverse arcsine variance weights for both the fixed and random effects models, and reported with a 95% confidence interval (95% CI) (20). We applied DerSimonian-Laird weights for the random effects model where heterogeneity between studies was found (21, 22). The I^2^ statistic was calculated as a measure of the proportion of the overall variation attributable to between-study heterogeneity (23). Funnel plots were used to explore publication bias across at least 10 studies, although the usefulness for non-intervention studies is uncertain. Selective reporting within studies was assessed by comparing available protocols with the reports. We described the seasonality pattern by geographic region from 2017 to 2013 based on PAHO surveillance data (24).

#### 2.5.1 Subgroup analysis, sensitivity analysis, and investigation of heterogeneity

If the number of studies allowed, we performed subgroup analyses based on to study design, dataset year, age group (<6 months, 6 to 12 months, 1–2 years, 3 to 5 years, 6 to 14 years, 15 to 65 years, and > 65 years), and the risk level of the population (no risk conditions/average risk, underlying medical conditions/risk factor). If these groups were only reported in wider age groups (i.e., 0–5 years) we also presented them. Additionally, we conducted a sensitivity analysis to assess the impact of risk of bias on the results of the primary analyses by limiting the analysis to studies with low risk of bias in the main domains.

## 3 Results

**Figure 1:**
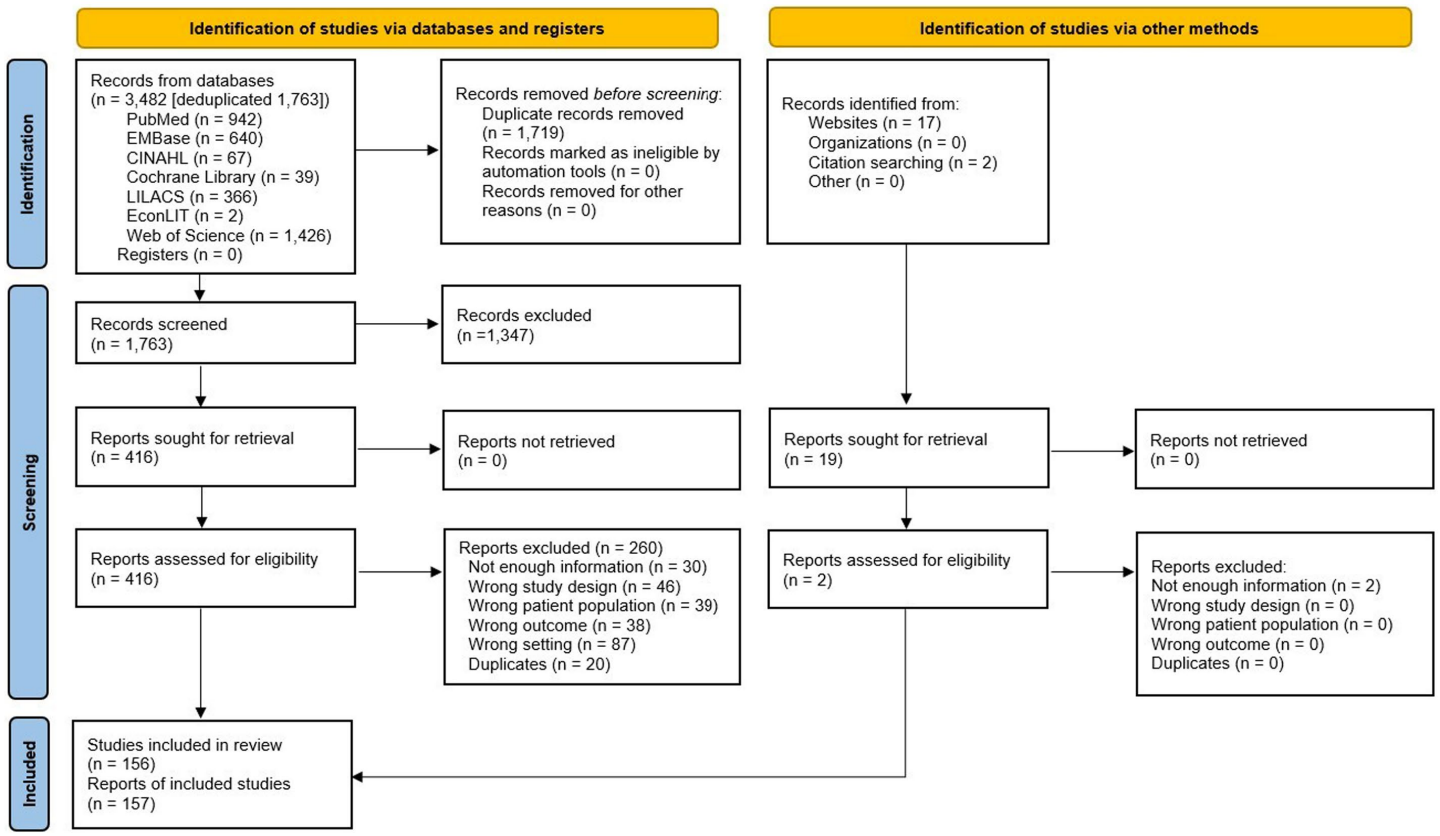
Study flowchart.

We identified 3,482 records in seven different databases, and after eliminating duplicates, we screened the 1,763 remaining records by title and abstract and 416 potentially eligible reports through full-text assessment. Finally, 156 studies (157 records) met the inclusion criteria (Figure 1; Supplementary Table 1). The list of studies excluded and their exclusion reasons are available in the Supplementary Table 2.

### 3.1 Characteristics of the included studies

Supplementary Table 1 summarizes the characteristics of the included studies. We included 156 studies: 125 full texts, 21 congress abstracts, and 6 theses. Among the studies included, there were 4 guidelines. Regarding the methodological design, there were 117 cross-sectional studies, 31 case series, 3 prospective cohorts, and 1 case–control study. The studies were carried out in 20 countries, and the most represented were Brazil (25), Colombia (22), Argentina (21), Chile (15), Peru (9), and Mexico (8). Nine studies included multiple countries from LAC. The articles were published between 2013 and 2022, and the inclusion period of participants was from 2000 to 2022, although we only included data from participants recruited from the year 2012 onwards. A total of 16 (10.5%) studies included participants during the years 2019–2020 of the COVID-19 pandemic. The reported duration of the studies ranged from one to 165 months.

Regarding the study population, 112 (73.7%) studies included pediatric patients, 22 (14.5%), both pediatric and adults, 10 (6.6%) only adult patients, and 8 (5.2%) studies did not report the age of the participants. Only 76 (50%) studies reported the clinical risk of the participants (e.g., prematurity, chronic conditions, immunocompromised), with 51 (67.1%) studies that included normal-risk participants, 18 (23.7%) high-risk, and 7 (9.2%) a mix of participants. Regarding the setting, 113 (85.6%) studies included inpatients, 17 (12.9%) included outpatients, and 2 (1.5%) included both.

A total of 1,445,198 samples, with 18% testing positive for RSV, along with epidemiological data from 69,891 patients, were included in the analysis. The diagnostic methods used were polymerase chain reaction (PCR) in 76 (50%) studies, Immunofluorescence (IF) in 36 (23.7%), direct (DIF) in 11 and indirect (IFI) in 17, immunochromatographic rapid test in nine (5.9%), mixed methods in eight (5.3%). Only 27 (17.8%) studies evaluated the type of RSV (A or B).

### 3.2 Risk of bias of the included studies

For cross-sectional and cohort studies, 57 (47.5%) were assessed as being at low risk (good quality), 56 (46.7%) were assessed as moderate risk (fair quality), and only seven (5.8%) as high risk (poor quality). The most frequent domains that did not meet appropriateness were related to sample size justification, the evaluation of exposures more than once or different levels of exposure, and the participation rate of eligible persons. It should be noted that many domains did not apply to the objectives of the included studies, such as blinding of the evaluators, assessment of exposure before the outcome, sufficiency of timeframe, percentage of loss to follow-up, and evaluation of potential confounding variables. For case series studies, 23 (74.2%) were rated as low risk, 6 (19.4%) as moderate risk, and only two (6.4%) as high risk. The domain that did not meet appropriateness more frequently was whether the cases were consecutive. Only one case–control study was included and rated as moderate risk due to a lack of information about the selection of participants, the use of concurrent controls, the blinding of the assessors, and because potential confounding variables were not measured. The risk of bias assessment by study design is presented in Supplementary Tables 3–5.

### 3.3 RSV burden of disease

**Figure 2:**
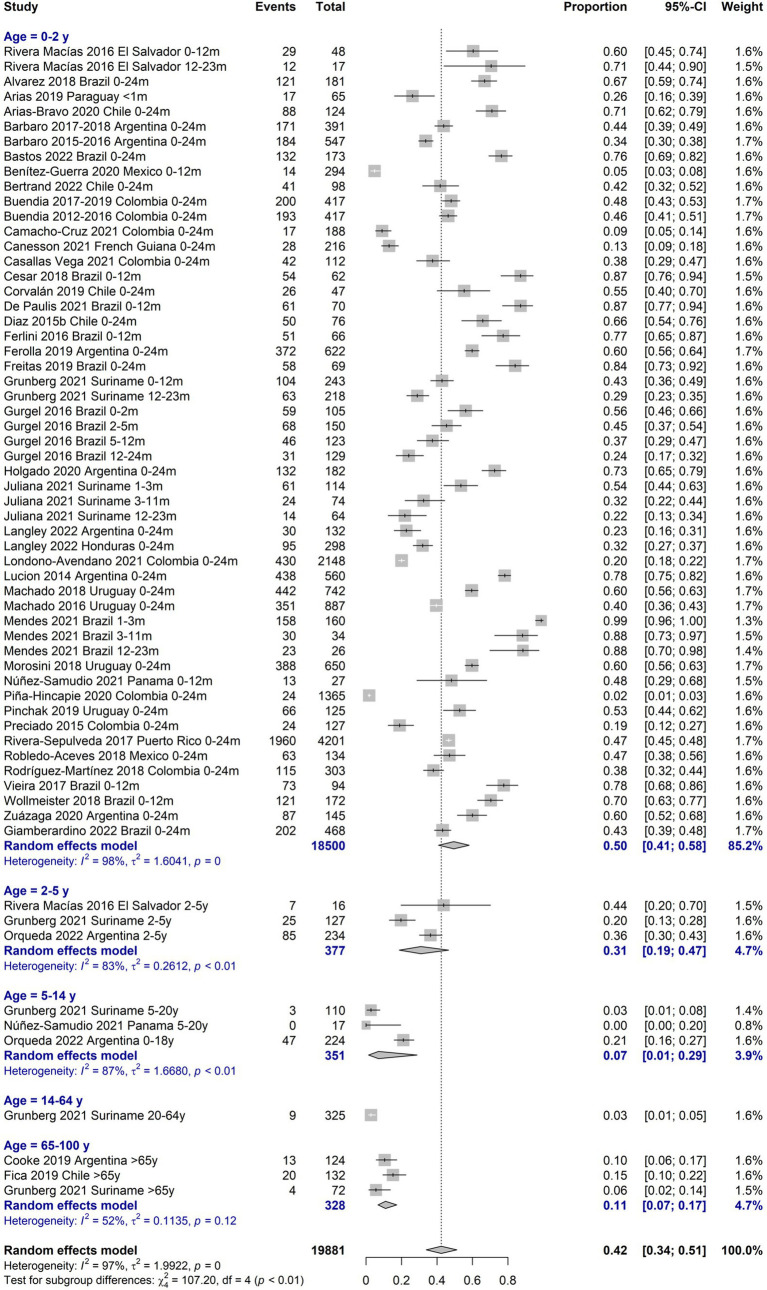
Proportion meta-analysis of RSV/suspected cases all ages.

Table 1 describes the main findings of RSV burden of disease by age groups with separate estimations for periods 2012–2016 and 2017–2022, for low-risk of bias (RoB) studies, and for high-risk patients when available. Regarding the prevalence of RSV infections, the age group most represented among the included studies was 0–24 months (38 studies), mostly presenting a low RoB. The positivity prevalence among suspected cases in this age group was 42.3% (95%CI 34.1–51.0), with the highest prevalence of 57.6% (95%CI 38.7–74.5) among 0-12-month-old infants. The age group of ≥65 years was poorly represented (3 studies), with a prevalence of 10.7% (95%CI 6.7–17.3; Figure 2).

**Table 1:** The epidemiological burden of RSV disease in Latin America.

Condition	Age	Studies (N)	Pooled estimation %; (CI95%)	I^2^
RSV/suspected cases	0–12 months	12	57.6 (38.7 to 74.5)	95.8%
	12–24 months	4	40.3 (22.2 to 61.4)	89.7%
	0–24 months	38	42.3 (34.1 to 51.0)	98.4%
	2012–2016	39	51.6 (41.2 to 62.0)	98%
	2017–2022	11	46.0 (31.3 to 61.4)	94.9%
	HR	4	16.7 (1.7 to 70.0)	98.8%
	Low ROB	31	54.6 (45.0 to 63.8)	97.6%
	2–5 years	3	30.1 (18.7 to 46.5)	82.7%
	5–14 years	3	7.0 (1.4 to 28.7)	87.3%
	2012–2016	1	2.8 (0.2 to 32.2)	–
	2017–2022	2	8.5 (1.0 to 45.5)	92.7%
	14–64 years	1	2.8 (1.5 to 5.2)	–
	≥65 years	3	10.7 (6.7 to 17.3)	52.0%
	2012–2016	1	15.2 (10.0 to 22.3)	–
	2017–2022	2	8.6 (4.8 to 15.0)	26.1%
	HR	2	13.0 (9.03 to 18.4)	98.4%
RSV LRTI/100 symptomatic p-y (mean)	0–6 months	2	21.6 (9.0 to 34.2)	68.2%
	6–12 months	2	19.7 (12.9 to 26.6)	0.0%
	12-23 months	2	9.9 (7.6 to 12.3)	0.0%
	0–24 months	2	14.7 (7.8 to 21.7)	94.4%
LRTI/RSV cases	0–24 months	3	72.1 (9.0 to 98.5)	98.7%
	2012–2016	2	37.6 (3.9 to 89.9)	98.1%
	2017–2022	1	98.0 (94.8 to 99.3)	–
	Low RoB	2	73.0 (0.95 to 99.9)	99.2%
	0–5 years	2	69.8 (62.3 to 76.1)	0.0%
	2012–2016	1	72.5 (61.7 to 81.2)	–
	2017–2022	1	67.7 (57.9 to 76.1)	–
	0–18 years	3	73.6 (38.9 to 92.4)	66.6%
	2012–2016	2	87.8 (61.9 to 96.9)	0.0%
	2017–2022	1	51.5 (39.7 to 63.1)	–
Lethality of RSV LRTI	0–24 months	7	0.6 (0.3 to 1.0)	0.0%
	2012–2016	6	0.6 (0.4 to 1.1)	0.0%
	2017–2022	1	0.3 (0.02 to 3.9)	–
	Low RoB	6	0.5 (0.3 to 1.0)	0.0%
	0–5 years	5	2.9 (0.7 to 1.7)	91.9%
	2012–2016	2	1.6 (0.02 to 56.0)	89.3%
	2017–2022	2	2.8 (0.2 to 28.0)	96.9%
	≥65 years-HR	1	23.1 (7.6 to 52.2)	–
Bronchiolitis/RSV cases	0–24 months	2	74.8 (35.3 to 94.1)	97.9%
	0–5 years	4	56.9 (29.5 to 80.6)	98.6%
	2012–2016	2	35.2 (26.5 to 45.1)	37.5%
	2017–2022	2	76.0 (46.3 to 92.1)	97.4%
	HR	1	30.5 (21.5 to 41.2)	–
	Low RoB	3	62.2 (27.1 to 87.9)	98.6%
Bronchiolitis/LRTI cases	0–2 years	2	76.4 (33.4 to 95.4)	98.1%
	0–5 years	4	70.3 (36.7 to 90.6)	97.9%
	2012–2016	2	42.2 (20.1 to 66.8)	88.0%
	2017–2022	2	86.8 (81.9 to 90.6)	30.4%
	HR	1	30.5 (21.5 to 41.2)	–
	Low RoB	3	74.8 (30.6 to 95.2)	98.3%
ICU admissions /RSV LRTI	0–12 months	2	20.1 (14.6 to 28.2)	0.0%
	0–24 months	8	42.0 (7.9 to 86.0)	92.0%
	2012–2016	4	14.9 (4.0 to 41.9)	85.3%
	2017–2022	4	23.2 (9.2 to 47.5)	92.0%
Length of stay in ICU (days)	0–24 months	5	3.2 (0.1 to 6.2)	91.0%
	2012–2016	2	1.7 (0 to 3.9)	0%
	2017–2022	1	8.0 (6.8 to 9.2)	–
Length of stay in general ward (days)	0–24 months	5	6.5 (4.7 to 8.3)	95.0%
	2012–2016	1	9.2 (7.9 to 10.5)	–
	2017–2022	4	6.4 (4.4 to 8.5)	94.5%
Antibiotic use/inpatients RSV cases	0–24 months	5	47.4 (29.5 to 66.1)	96.6%
	2012–2016	2	56.0 (26.7 to 81.6)	97.1%
	2017–2022	2	51.6 (22.6 to 79.5)	97.5%
Invasive ventilation/RSV cases	0–12 months	3	10.4 (4.3 to 23.2)	77.6%
	0–24 months	9	13.6 (7.22 to 24.1)	90.9%
	2012–2016	3	7.3 (4.7 to 11.2)	0.0%
	2017–2022	6	18.1 (8.3 to 35.0)	91.2%
	Low RoB	7	18.8 (11.2 to 29.8)	89.6%
	0–5 years	4	31.4 (16.1 to 52.2)	87.6%
	2012–2016	3	29.6 (11.0 to 58.9)	91.7%
	2017–2022	1	36.2 (24.9 to 49.2)	–
	Low RoB	4	31.4 (16.1 to 52.2)	87.6%
Type of RSV/viral isolates	0–6 months RSV A	1	76.7 (65.7 to 85.0)	–
	0–12 months RSV A	3	31.6 (6.4 to 75.8)	96.5%
	0–24 months RSV A	11	51.6 (33.6 to 69.2)	96.2%
	2012–2016	8	57.3 (35.9 to 76.3)	94.4%
	2017–2022	3	36.8 (12.3 to 70.8)	96.9%
	Low RoB	6	52.1 (31.2 to 72.3)	96.1%
	≥65 years RSV A	1	15.4 (1.9 to 45.5)	–
	0–12 months RSV B	4	46.3 (24.8 to 69.3)	94.3%
	0–24 months RSV B	11	34.4 (25.5 to 44.5)	91.2%
	2012–2016	8	37.1 (25.1 to 50.9)	92.6%
	2017–2022	3	26.9 (21.5 to 33.2)	39.3%
	Low RoB	6	28.4 (22.9 to 34.6)	58.3%
Coinfection/RSV cases	0–24 months Viral	14	20.8 (12.0 to 33.6)	92.2%
	2012–2016	10	24.1 (13.9 to 38.6)	89.7%
	2017–2022	3	7.4 (1.9 to 24.4)	82.5%
	Low RoB	7	18.2 (9.6 to 32.0)	92.8%
	≥65 years-HR Viral	2	20.4 (1.1 to 86.0)	79.1%
	0–18 years Bacterial	2	19.7 (3.6 to 61.6)	93.4%

RSV, Respiratory syncytial virus; p-y, person-years; HR, High risk (e.g., prematurity, chronic conditions, immunocompromised); LRTI, lower respiratory tract infection; RoB, Risk of bias.

**Figure 3:**
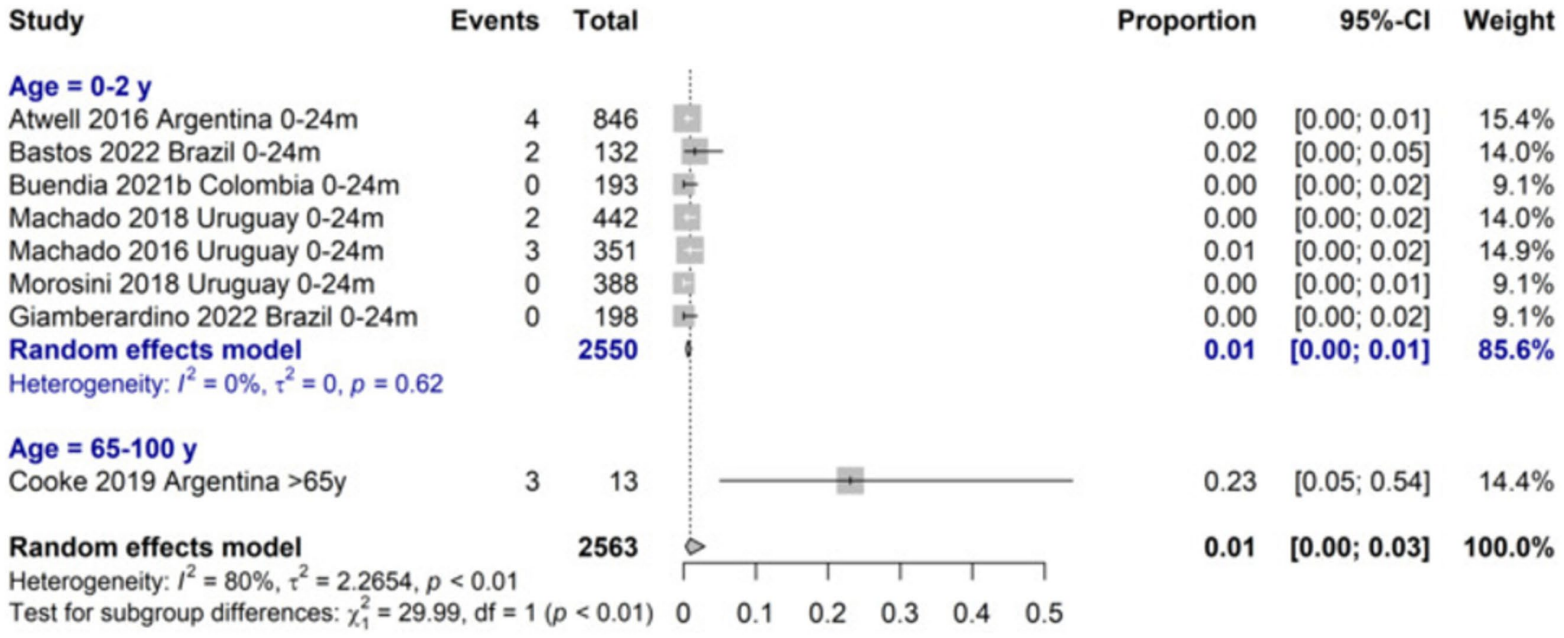
Proportion meta-analysis of lethality of RSV LRTI cases all ages.

The incidence of LRTI due to RSV was 14.7 (95%CI 7.8–21.7) per 100 symptomatic 0–24 months-old infants and higher for the 0–6 months age group (21.6%; 95%CI 9.0–34.2). The proportion of LRTI among RSV cases among 0–24 months-old infants was 72.1 (95%CI 9.0 to 98.5) and was similar for children aged 0 to 18 years old. The RSV-LRTI lethality was 0.6% (95%CI 0.3–1.0) in the 0–24 months and 2.9% (95%CI 0.7–1.7) in the 0–5 years old group, respectively. The ≥65-year lethality was estimated to be 23.1% (7.6 to 52.2) based on a single study on a high-risk population (Figure 3). Among RSV-LRTI cases, studies reporting bronchiolitis showed 76.4% (95%CI 33.4 to 95.4) and 70.3% (95%CI 36.7 to 90.6) in the 0–24 months and 0–5 years old, respectively.

The highest proportion of ICU admissions among RSV-LRTI was 42.0% (95%CI 7.9–86.0) for the 0–24 months age group, with a mean length of stay at ICU of 3.2 days (0.1 to 6.2) and general ward of 6.5 days (4.7 to 8.3), respectively. Antibiotic use in children aged 0–24 months was 47.4, and 13.6% of this age group required invasive ventilation.

RSV type A presented a higher proportion than type B from 0 to 24 months (51.6% vs. 34.4, respectively), being more markedly at lower age. The most frequent co-infections were viral with similar percentages in younger and older patients, 20.8% between 0 and 24 months and 20.4% in ≥65 years, with more coinfections detected in the youngest during 2012–2016 compared to 2017–2022 (24.1% vs. 7.4%). The summary estimates reported in Table 1 are derived from meta-analyses, which forest plots are available in the Supplementary Figures 1–5. The heterogeneity was high for most meta-analyses.

### 3.4 Immunoprophylaxis recommendations: evaluation of guidelines

Based on our literature search, we identified four guidelines for treating RSV in the LATAM region. The guidelines were developed in Argentina (26), Honduras (27), Brazil (28), and Chile (29). Three guidelines focused on the treatment and prevention of RSV-related bronchiolitis, while one focused on bronchiolitis of any viral cause (27). The assessed guidelines show notable methodological limitations. They are narrative reviews without systematic searches, clear methodologies, external expert reviews, and a proposal to update existing guidelines. They lack clear overall objectives (26, 27), and only one guideline specifies the aim of reducing RSV hospitalizations (28). Beneficiaries and outcomes are vaguely described (26, 27). Target populations are not mentioned consistently throughout the guidelines. While one outlines it briefly (26), another specifies it in detail (27). No guideline provides a specific health question. Treatment recommendations are included for premature infants. However, the infants’ age is presented ambiguously (26, 29) or not specified (27, 28). Author details are only fully provided in one guideline (29). No guidelines included views of the target population or provided information on literature searches. Three of the four guidelines do not mention the treatment benefits, side effects, and risks (27–29). Recommendations vary in specificity from general (27) to specific (26, 28, 29). In three guidelines, these recommendations are linked to clinical evidence from previously conducted studies (26, 27, 29). Facilitators, barriers, and stakeholders are not described. All guidelines lack implementation guidance and resource implications. Cost-effectiveness and economic data are missing, and monitoring criteria are not presented. Editorial independence is mentioned in only one guideline (27). The complete evaluation of the assessment of guidelines is in Supplementary Table 6.

These four guidelines provide recommendations for palivizumab use in vulnerable populations, offering insights into inclusion criteria, prescription procedures, post-administration serum levels, and epidemiological data related to RSV infection. There is consensus recommending palivizumab in premature babies less than 29 weeks, without chronic pulmonary disease in premature babies younger than 12 months old at the start of RSV station. Also, to infants under 12 months with hemodynamically significant heart disease or infants under 24 months of age undergoing heart transplant during RSV season.

### 3.5 RSV seasonality

**Figure 4:**
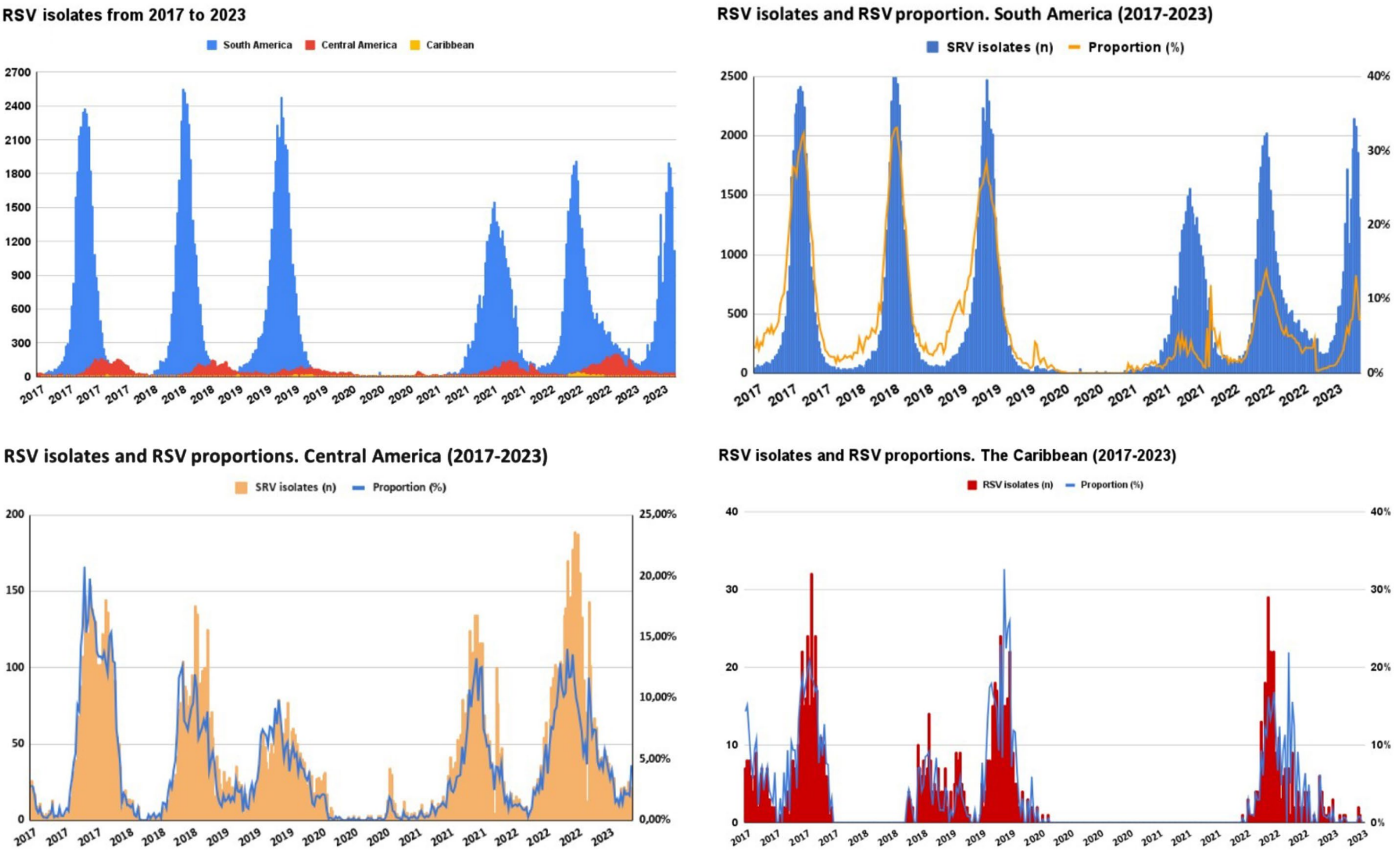
Seasonality pattern of RSV isolates 2017–2023 in South America^a^, Central/North America^b^, and the Caribbean^c^. ^a^Argentina, Bolivia, Brazil, Chile, Colombia, Ecuador, Paraguay, Perú, Uruguay, and Venezuela. ^b^Costa Rica, El Salvador, Guatemala, Honduras, Mexico, Nicaragua, and Panama. ^c^Cuba, Dominican Republic, and Haiti.

The Figure 4 shows the seasonality pattern of RSV isolates from 2017 to 2023 across three geographical regions: South America, Central/North America, and the Caribbean. The time series, from PAHO surveillance, data show a clear cyclical pattern for each region. South America exhibits pronounced peaks in RSV isolates each year in the winter season, having the highest number of isolates; the intensity of these peaks seems to be diminishing slightly over the years. Central America’s RSV isolates also follow a seasonal trend. Still, the peaks are less pronounced than those in South America, relatively consistent over the years, with no notable increase or decrease in the number of isolates. The Caribbean shows the least pronounced peaks among the three regions and is more spread out, with a noticeable increase in recent years. Of note, there was a clear gap during the pandemic (2020–2021).

## 4 Discussion

Our systematic review updates a previous review (10) to evaluate the burden of disease of RSV in the Latin American pediatric and adult population, and, as far as we know, includes the largest number of studies and patients in our region. A total of 156 studies and epidemiological data from 69,891 patients from LAC were included in this study. We identified mainly cross-sectional studies with low risk of bias, with Brazil, Colombia, and Argentina being the most represented countries in the region. National reports from many LAC countries have limited information, likely due to the absence of mandatory reporting and passive surveillance of RSV. In agreement with our review, most systematic reviews reported data on pediatric inpatients and generally involved healthy individuals (3, 30–33). In the countries of LAC, the RSV laboratory surveillance program is predominantly conducted in pediatric patients requiring hospitalization (34), consequently resulting in a higher publication rate of studies focusing on this patient group. Consistently with this pattern, we found only one review assessing the epidemiology of RSV in adults and the older adults in LAC (11).

RSV is the primary cause of bronchiolitis and viral community-acquired pneumonia in infants, and LRTI in the older population. It can lead to life-threatening respiratory disease, particularly in patients with risk factors such as prematurity, young age, and comorbidities such as hemodynamically significant congenital heart disease and chronic lung disease of prematurity (5, 6). A long-term effect following RSV infection in infants is the risk of subsequent wheezing in children, especially if the initial infection requires hospitalization, and 20 to 40% experience recurrent episodes of asthma (35–37). We observed a high pooled RSV prevalence in children under 2 years of age, especially in those under 6 months, with a higher proportion of serotype A infection compared to serotype B. Similar findings have been reported globally (38).

A national study in the US revealed that infants with high-risk comorbidities hospitalized due to RSV during the period from 1997 to 2012 had a fivefold increase in mechanical ventilation requirement compared to infants without high-risk conditions. RSV imposes a significant disease burden on pediatric patients, with hospitalization rates three times higher than those associated with other respiratory viruses (25, 39).

We found that RSV-LRTI ICU admission was 42% and the requirement of invasive ventilation was 14% among 0-2-year-old patients. The RSV-LRTI lethality was 0.6, 3%, and in patients aged <2, 0–5 years, respectively, and 23% among >65 years old high-risk patients. However, the elevated risk of this older adult population explains the high rate reported by a single study. Li et al. estimated that the adjusted hospitalization rate for adults aged 65 years and older was 347 per 100,000 (95%CI 203–595) with an age-dependent increase, ranging from 231 per 100,000 in adults aged 65–74 years to 692 per 100,000 in adults aged 85 years or more (8). Ali et al. reported a high rate of hospitalization due to RSV-infected Mexican adults with influenza-like illness (40.9–69.9%). Liu Li et al. reported, the in-hospital case fatality ratio (CFR) for RSV was 6.1% (95%CI 3.3–11.0) (8) and Savic et al. (5) 7.1% (95%CI 5.4–9.4) among individuals aged 60 years or older (7). Worldwide, RSV claims the lives of over 100,000 children annually, with approximately half of these deaths occurring in infants under 6 months of age. The majority of these fatalities take place in countries with limited resources (3). Our CFR estimations highlighted the magnitude of the problem for a very frequent disease.

Regarding resource utilization, we found an average length of stay of 3.2 days and 6.5 days in the ICU and the general ward in patients aged under 24 months. These findings are aligned with previous reports (40–47). In the group under 2 years of age, RSV infection leads to increased antimicrobial use. A study reported that up to one-third of children with RSV LRTI, without co-bacterial infections, receive unnecessary antibiotic treatment (48). Similarly, we reported viral coinfection occurring in both the group under 2 years and in those over 65, at approximately 21%. Other pediatric cohort studies exhibit a greater rate of viral co-detections at 55% (49), falling between the 34% reported in the US EPIC study (50), focused on viral detection in children under 5 years old with pneumonia, and the 61% documented by the ORAACLE Study Group, a Norwegian clinical cohort, that investigated the length of stay of hospitalized patients with bronchiolitis (51). The variations in these findings may be attributed to the precision of the novel molecular detection methods employed.

Differences in seasonality were previously reported worldwide (52) and in LAC (10, 53). In the Southern Hemisphere, RSV circulates primarily during the late fall or early winter season, characterized by marked peaks in circulation. In contrast, Central American countries exhibit a seasonal trend, but with less pronounced peaks. These observed differences likely stem from variations in climatic patterns that are linked to the behavior of RSV in each region. During the first year of the pandemic of SARS-CoV-2, a gap in RSV circulation was observed in the countries of the region. It may be attributed to the new virus infection displacing the opportunity for RSV circulation. This, combined with strict isolation measures and the initial closure of schools and daycare centers implemented during the pandemic, could have contributed to the observed phenomenon. Numerous studies worldwide evaluate the effectiveness of non-pharmacological interventions (NPIs) in containing the spread of respiratory viruses, primarily SARS-CoV-2 and Influenza (54–58). NPIs aimed at limiting the spread of SARS-CoV-2 appear to have affected the circulation of other respiratory viruses. An increase in the percentage of positivity and the diversity of respiratory viruses was observed as the degree of restriction decreased (59).

Four guidelines addressing the use of palivizumab for the prevention of RSV infection in high-risk populations in Latin America were identified (26–29). However, all of them present serious methodological limitations which limit their ability to offer definitive conclusions regarding the effectiveness and clinical applicability of palivizumab in the region. Therefore, it is highlighted that further work is needed to develop high quality guidelines and recommendations using the GRADE approach (60) to enhance RSV infection prevention in Latin America.

Our study presents some limitations. We did not find population-based cohort studies, which provide the best burden of disease estimates. The limited number of reports conducted in the adult population, as well as in outpatients, concerning the burden of RSV indicates a gap in information in these populations. Due to the different testing methods employed, it is expected variability in RSV estimates, particularly for the adult population, where certain testing methods are known to have lower sensitivity. Consequently, the results may underestimate the true burden of RSV in adults. Standardizing testing approaches could provide more accurate estimates of RSV prevalence. Unfortunately, the data for this age group was scarce avoiding subgroup analysis by testing method. The literature search was conducted in January 2023, therefore some latest published studies could be missed and findings cannot be extrapolated beyond this date. Finally, most meta-analyses showed high levels of heterogeneity. Meta-analysis from observational studies usually exhibits greater heterogeneity in both the frequency and magnitude than pooling experimental studies. This inherent variability stems from the real-world nature of epidemiological research, which encompasses diverse populations, environmental exposures, and healthcare settings. However, we partially addressed this issue by employing the random-effects model that yields broader and more conservative confidence intervals. When faced with high levels of heterogeneity, these confidence intervals provide a more reliable estimation than the point estimates (61). On the other side, our systematic review also has relevant strengths. We conducted exhaustive searches across multiple databases and found more studies than any published systematic review on this topic in LAC. We independently screened, extracted, and assessed the risk of bias in each study by pairs of reviewers, ensuring a comprehensive evaluation of the available evidence, and performed proportion meta-analyses. In summary, we followed a rigorous methodology that also considered RSV types, population risks, seasonality, time variations, and the risk of bias in included studies. Finally, we need to be well prepared in Latin America in terms of knowing the epidemiology, impact, disease burden, associated costs, and microbiological aspects of RSV in both children and adults to achieve a smoother road for new monoclonal antibodies and vaccine introductions (62). The findings from this systematic review provide crucial insights for physicians and health policymakers by highlighting the significant burden of RSV in LAC, particularly among infants under one-year-old and high-risk older adult patients. Policymakers should consider integrating RSV vaccination programs, especially targeting the most vulnerable populations. Furthermore, the observed seasonality patterns of RSV, more pronounced in South America, suggest that timing vaccination and immunoprophylaxis efforts to align with peak RSV seasons could enhance their effectiveness. Recommendations include the establishment of robust surveillance systems to continuously monitor RSV incidence and prevalence. These strategies will help reduce the healthcare burden and improve patient outcomes in Latin America.

## 5 Conclusion

This comprehensive body of evidence thoroughly describes the significant impact of RSV in LAC, especially among the very young and older adult populations, along with its seasonal patterns. Our findings have the potential to inform and enhance strategies for the effective prevention and treatment of this critical public health issue. Additionally, there are some implications for research, including the need for population-based cohort studies that better represent the extremes of life and outpatient settings.

## Data Availability

The datasets presented in this study can be found in online repositories. The names of the repository/repositories and accession number(s) can be found in the article/Supplementary material.
