# Supplementary material for: Respiratory syncytial virus disease burden in children and adults from Latin America: a systematic review and meta-analysis
Source: Front Public Health. 2024 Oct 16;12:1377968. doi: 10.3389/fpubh.2024.1377968 (PMC11521816; doi:10.3389/fpubh.2024.1377968)
Supplement: Supplementary file 1 [file Data_Sheet_1.docx]

Index Supplementary Material

[1 PRISMA checklist 4](#_Toc157340056)

[2 Search strategy and selection of studies 5](#_Toc157340057)

[3 Grey literature sources 12](#_Toc157340058)

[4 Supplementary Table 1. Characteristics of the included studies 13](#_Toc157340059)

[5 Supplementary Table 2. Studies excluded at full text screening stage 16](#_Toc157340060)

[6 Risk of bias assessment instruments 17](#_Toc157340061)

[6.1 Supplementary Table 3. Risk of bias assessment for cohort and cross-sectional studies 17](#_Toc157340062)

[6.2 Supplementary Table 4. Risk of bias assessment for case series studies 20](#_Toc157340063)

[6.3 Supplementary Table 5. Risk of bias assessment for case-control studies 20](#_Toc157340064)

[7 Supplementary Table 6. AGREE-ll assessment for guidelines 21](#_Toc157340065)

[8 Burden of RSV disease: meta-analysis 23](#_Toc157340066)

[8.1 Supplementary Figure 1. RSV/suspected cases 0-2 years 23](#_Toc157340067)

[8.2 Supplementary Figure 2. RSV/suspected cases 0-2 years by five year calendar period (2012-2016 and 2017-2022) 24](#_Toc157340068)

[8.3 Supplementary Figure 3. RSV/suspected cases 0-2 years by risk group (high and normal risk) 24](#_Toc157340069)

[8.4 Supplementary Figure 4. RSV/suspected cases 0-2 years by low ROB 26](#_Toc157340070)

[8.5 Supplementary Figure 5. RSV/suspected cases 5-14 years by five year calendar period (2012-2016 and 2017-2022) 26](#_Toc157340071)

[8.6 Supplementary Figure 6. RSV/suspected cases ≥65 years by five year calendar period (2012-2016 and 2017-2022) 27](#_Toc157340072)

[8.7 Supplementary Figure 7. RSV/suspected cases ≥65 years by risk group (high and normal risk) 27](#_Toc157340073)

[8.8 Supplementary Figure 8. RSV LRTI/100 symptomatic p-y (mean) 0-2 years 27](#_Toc157340074)

[8.9 Supplementary Figure 9. LRTI/RSV cases 0-2 years 27](#_Toc157340075)

[8.10 Supplementary Figure 10. LRTI/RSV cases 0-2 years by five year calendar period (2012-2016 and 2017-2022) 28](#_Toc157340076)

[8.11 Supplementary Figure 11. LRTI/RSV cases 0-2 years by low ROB 28](#_Toc157340077)

[8.12 Supplementary Figure 12. LRTI/RSV cases 0-5 years by five year calendar period (2012-2016 and 2017-2022) 29](#_Toc157340078)

[8.13 Supplementary Figure 13. LRTI/RSV cases 0-18 years by five year calendar period (2012-2016 and 2017-2022) 29](#_Toc157340079)

[8.14 Supplementary Figure 14. Lethality of RSV LRTI 0-2 years by five year calendar period (2012-2016 and 2017-2022) 29](#_Toc157340080)

[8.15 Supplementary Figure 15. Lethality of RSV LRTI 0-2 years by low ROB 30](#_Toc157340081)

[8.16 Supplementary Figure 16. Lethality of RSV LRTI 0-5 years 30](#_Toc157340082)

[8.17 Supplementary Figure 17. Lethality of RSV LRTI 0-5 years by five year calendar period (2012-2016 and 2017-2022) 30](#_Toc157340083)

[8.18 Supplementary Figure 18. Bronchiolitis/RSV cases 0-2 years 31](#_Toc157340084)

[8.19 Supplementary Figure 19. Bronchiolitis/RSV cases 0-5 years by five year calendar period (2012-2016 and 2017-2022) 31](#_Toc157340085)

[8.20 Supplementary Figure 20. Bronchiolitis/RSV cases 0-5 years by risk group (high and normal risk) 31](#_Toc157340086)

[8.21 Supplementary Figure 21. Bronchiolitis/RSV cases 0-5 years by low ROB 32](#_Toc157340087)

[8.22 Supplementary Figure 22. Bronchiolitis/LRTI cases 0-2 years 32](#_Toc157340088)

[8.23 Supplementary Figure 23. Bronchiolitis/LRTI cases 0-5 years 32](#_Toc157340089)

[8.24 Supplementary Figure 24. Bronchiolitis/LRTI cases 0-5 years by five year calendar period (2012-2016 and 2017-2022) 33](#_Toc157340090)

[8.25 Supplementary Figure 25. Bronchiolitis/LRTI cases 0-5 years by risk group (high and normal risk) 33](#_Toc157340091)

[8.26 Supplementary Figure 26. Bronchiolitis/LRTI cases 0-5 years by low ROB 33](#_Toc157340092)

[8.27 Supplementary Figure 27. ICU admissions /RSV LRTI 0-2 years 34](#_Toc157340093)

[8.28 Supplementary Figure 28. ICU admissions /RSV LRTI 0-2 years by five year calendar period (2012-2016 and 2017-2022) 34](#_Toc157340094)

[8.29 Supplementary Figure 29. Length of stay in ICU (days) 0-2 years 35](#_Toc157340095)

[8.30 Supplementary Figure 30. Length of stay in ICU (days) 0-2 years by five year calendar period (2012-2016 and 2017-2022) 35](#_Toc157340096)

[8.31 Supplementary Figure 31. Length of stay in general ward (days) 0-2 years 35](#_Toc157340097)

[8.32 Supplementary Figure 32. Length of stay in general ward (days) 0-2 years by five year calendar period (2012-2016 and 2017-2022) 36](#_Toc157340098)

[8.33 Supplementary Figure 33. Antibiotic use/inpatients RSV cases 0-2 years 36](#_Toc157340099)

[8.34 Supplementary Figure 34. Antibiotic use/inpatients RSV cases 0-2 years by five year calendar period (2012-2016 and 2017-2022) 36](#_Toc157340100)

[8.35 Supplementary Figure 35. Invasive ventilation/RSV LRTI cases 0-2 years 36](#_Toc157340101)

[8.36 Supplementary Figure 36. Invasive ventilation/RSV LRTI cases 0-2 years by five year calendar period (2012-2016 and 2017-2022) 37](#_Toc157340102)

[8.37 Supplementary Figure 37. Invasive ventilation/RSV LRTI cases 0-2 years by low ROB 38](#_Toc157340103)

[8.38 Supplementary Figure 38. Invasive ventilation/RSV LRTI cases 0-5 years by five year calendar period (2012-2016 and 2017-2022) 38](#_Toc157340104)

[8.39 Supplementary Figure 39. Invasive ventilation/RSV LRTI cases 0-5 years by low ROB 38](#_Toc157340105)

[8.40 Supplementary Figure 40. RSV A/viral isolates 0-2 years 39](#_Toc157340106)

[8.41 Supplementary Figure 41. RSV A/viral isolates 0-2 years by five year calendar period (2012-2016 and 2017-2022) 39](#_Toc157340107)

[8.42 Supplementary Figure 42. RSV A/viral isolates 0-2 years by low ROB 40](#_Toc157340108)

[8.43 Supplementary Figure 43. RSV A/viral isolates ≥65 years 40](#_Toc157340109)

[8.44 Supplementary Figure 44. RSV B/viral isolates 0-2 years 40](#_Toc157340110)

[8.45 Supplementary Figure 45. RSV B/viral isolates 0-2 years by five year calendar period (2012-2016 and 2017-2022) 41](#_Toc157340111)

[8.46 Supplementary Figure 46. RSV B/viral isolates 0-2 years by low ROB 41](#_Toc157340112)

[8.47 Supplementary Figure 47. Viral coinfection/RSV cases 0-2 years 42](#_Toc157340113)

[8.48 Supplementary Figure 48. Viral coinfection/RSV cases 0-2 years by five year calendar period (2012-2016 and 2017-2022) 42](#_Toc157340114)

[8.49 Supplementary Figure 49. Viral coinfection/RSV cases 0-2 years by low ROB 43](#_Toc157340115)

[8.50 Supplementary Figure 50. Viral coinfection/RSV ≥65 years 43](#_Toc157340116)

[8.51 Supplementary Figure 51. Bacterial coinfection/RSV cases 0-5 years by risk group (high and normal risk) 43](#_Toc157340117)

[8.52 Supplementary Figure 52. Bacterial coinfection/RSV cases 0-18 years 44](#_Toc157340118)

[References 45](#_Toc157340119)

# Supplementary Table 1. PRISMA checklist

| **Section and Topic** | **Item #** | **Checklist item** | **Location where item is reported** |
| --- | --- | --- | --- |
| **TITLE** | | |  |
| Title | 1 | Identify the report as a systematic review. | Title |
| **ABSTRACT** | | |  |
| Abstract | 2 | See the PRISMA 2020 for Abstracts checklist. | Abstract |
| **INTRODUCTION** | | |  |
| Rationale | 3 | Describe the rationale for the review in the context of existing knowledge. | Section 1 |
| Objectives | 4 | Provide an explicit statement of the objective(s) or question(s) the review addresses. | Section 1 |
| **METHODS** | | |  |
| Eligibility criteria | 5 | Specify the inclusion and exclusion criteria for the review and how studies were grouped for the syntheses. | Section 2.1 |
| Information sources | 6 | Specify all databases, registers, websites, organisations, reference lists and other sources searched or consulted to identify studies. Specify the date when each source was last searched or consulted. | Section 2.2 and Supplementary material: Section 3 |
| Search strategy | 7 | Present the full search strategies for all databases, registers and websites, including any filters and limits used. | Supplementary material: Section 2 |
| Selection process | 8 | Specify the methods used to decide whether a study met the inclusion criteria of the review, including how many reviewers screened each record and each report retrieved, whether they worked independently, and if applicable, details of automation tools used in the process. | Section 2.3 |
| Data collection process | 9 | Specify the methods used to collect data from reports, including how many reviewers collected data from each report, whether they worked independently, any processes for obtaining or confirming data from study investigators, and if applicable, details of automation tools used in the process. | Section 2.3 |
| Data items | 10a | List and define all outcomes for which data were sought. Specify whether all results that were compatible with each outcome domain in each study were sought (e.g. for all measures, time points, analyses), and if not, the methods used to decide which results to collect. | Section 2.3 |
|  | 10b | List and define all other variables for which data were sought (e.g. participant and intervention characteristics, funding sources). Describe any assumptions made about any missing or unclear information. | Section 2.2 and Section 2.3 |
| Study risk of bias assessment | 11 | Specify the methods used to assess risk of bias in the included studies, including details of the tool(s) used, how many reviewers assessed each study and whether they worked independently, and if applicable, details of automation tools used in the process. | Section 2.4 |
| Effect measures | 12 | Specify for each outcome the effect measure(s) (e.g. risk ratio, mean difference) used in the synthesis or presentation of results. | Section 2.5 |
| Synthesis methods | 13a | Describe the processes used to decide which studies were eligible for each synthesis (e.g. tabulating the study intervention characteristics and comparing against the planned groups for each synthesis (item #5)). | Not described |
|  | 13b | Describe any methods required to prepare the data for presentation or synthesis, such as handling of missing summary statistics, or data conversions. | Not described |
|  | 13c | Describe any methods used to tabulate or visually display results of individual studies and syntheses. | Not described |
|  | 13d | Describe any methods used to synthesize results and provide a rationale for the choice(s). If meta-analysis was performed, describe the model(s), method(s) to identify the presence and extent of statistical heterogeneity, and software package(s) used. | Section 2.5.1 |
|  | 13e | Describe any methods used to explore possible causes of heterogeneity among study results (e.g. subgroup analysis, meta-regression). | Section 2.5.2 |
|  | 13f | Describe any sensitivity analyses conducted to assess robustness of the synthesized results. | Section 2.5.2 |
| Reporting bias assessment | 14 | Describe any methods used to assess risk of bias due to missing results in a synthesis (arising from reporting biases). | Section 2.5.2 |
| Certainty assessment | 15 | Describe any methods used to assess certainty (or confidence) in the body of evidence for an outcome. | Not described |
| **RESULTS** | | |  |
| Study selection | 16a | Describe the results of the search and selection process, from the number of records identified in the search to the number of studies included in the review, ideally using a flow diagram. | Section 3 and Figure 1 |
|  | 16b | Cite studies that might appear to meet the inclusion criteria, but which were excluded, and explain why they were excluded. | Supplementary material: Table 2 |
| Study characteristics | 17 | Cite each included study and present its characteristics. | Section 3.1 and Table 1 |
| Risk of bias in studies | 18 | Present assessments of risk of bias for each included study. | Section 3.2 and Supplementary material: Table 3, 4 and 5 |
| Results of individual studies | 19 | For all outcomes, present, for each study: (a) summary statistics for each group (where appropriate) and (b) an effect estimate and its precision (e.g. confidence/credible interval), ideally using structured tables or plots. | Table 2 |
| Results of syntheses | 20a | For each synthesis, briefly summarise the characteristics and risk of bias among contributing studies. | Table 2 |
|  | 20b | Present results of all statistical syntheses conducted. If meta-analysis was done, present for each the summary estimate and its precision (e.g. confidence/credible interval) and measures of statistical heterogeneity. If comparing groups, describe the direction of the effect. | Table 2 |
|  | 20c | Present results of all investigations of possible causes of heterogeneity among study results. | Not described |
|  | 20d | Present results of all sensitivity analyses conducted to assess the robustness of the synthesized results. | Not described |
| Reporting biases | 21 | Present assessments of risk of bias due to missing results (arising from reporting biases) for each synthesis assessed. | NA |
| Certainty of evidence | 22 | Present assessments of certainty (or confidence) in the body of evidence for each outcome assessed. | Not described |
| **DISCUSSION** | | |  |
| Discussion | 23a | Provide a general interpretation of the results in the context of other evidence. | Section 4 |
|  | 23b | Discuss any limitations of the evidence included in the review. | Section 4 |
|  | 23c | Discuss any limitations of the review processes used. | Section 4 |
|  | 23d | Discuss implications of the results for practice, policy, and future research. | Section 4 |
| **OTHER INFORMATION** | | |  |
| Registration and protocol | 24a | Provide registration information for the review, including register name and registration number, or state that the review was not registered. | Section 2 |
|  | 24b | Indicate where the review protocol can be accessed, or state that a protocol was not prepared. | Section 2 |
|  | 24c | Describe and explain any amendments to information provided at registration or in the protocol. | NA |
| Support | 25 | Describe sources of financial or non-financial support for the review, and the role of the funders or sponsors in the review. | Funding |
| Competing interests | 26 | Declare any competing interests of review authors. | Declaration of competing interest |
| Availability of data, code and other materials | 27 | Report which of the following are publicly available and where they can be found: template data collection forms; data extracted from included studies; data used for all analyses; analytic code; any other materials used in the review. | Data availability |

# Search strategy and selection of studies

1. **MEDLINE PubMed**

Date of search: 04/01/2023

| Search | Query |
| --- | --- |
| #10 | #7 AND #8 Filters: from 2012/1/1-3000/12/12 |
| #9 | #7 AND #8 |
| #8 | (Americas[Majr] OR Latin America[Mesh] OR Latin America*[tiab] OR Latinamerica*[tiab] OR Latinoamerica*[tiab] OR Hispanoamerica*[tiab] OR Iberoamerica*[tiab] OR Ibero Americ*[tiab] OR Panamerican*[tiab] OR Central America[Mesh] OR Central America*[tiab] OR Centroamerica*[tiab] OR Mesoamerica*[tiab] OR Meso America*[tiab] OR Middle America*[tiab] OR South America[Mesh] OR South America*[tiab] OR Southamerica*[tiab] OR Sudamerica*[tiab] OR "America del Sur"[tiab] OR Caribbean Region[Mesh] OR Caribbean[tiab] OR Caribe*[tiab] OR West Indies[Mesh] OR West Indi*[tiab] OR Antill*[tiab] OR Indians, South American[Mesh] OR Indians, Central American[Mesh] OR Amerindian*[tiab] OR Indians[tiab] OR American Indian*[tiab] OR Native America*[tiab] OR Patagoni*[tiab] OR Andes[tiab] OR Andean*[tiab] OR Amazon*[tiab] OR Anguilla[ad] OR Anguill*[tiab] OR Anguilla[pl] OR "Antigua and Barbuda"[ad] OR "Antigua and Barbuda"[tiab] OR "Antigua and Barbuda"[pl] OR Argentin*[ad] OR Argentin*[tiab] OR Argentina[pl] OR Bahama*[ad] OR Baham*[tiab] OR Bahama*[pl] OR Bermud*[ad] OR Bermud*[tiab] OR Bermud*[pl] OR Bolivia*[ad] OR Bolivia*[tiab] OR Bolivia[pl] OR Brazil*[ad] OR Brasil*[ad] OR Brazil*[tiab] OR Brasil*[tiab] OR Brazil[pl] OR Cayman*[ad] OR Cayman*[tiab] OR Cayman*[pl] OR Curaçao[ad] OR Curaçao[tiab] OR Curaçao[pl] OR Colombia*[ad] OR Colombia*[tiab] OR Colombia[pl] OR Chile*[ad] OR Chile*[tiab] OR Chile[pl] OR Ecuador*[ad] OR Ecuator*[ad] OR Ecuador*[tiab] OR Ecuador[pl] OR Grenad*[ad] OR Grenad*[tiab] OR Grenad*[pl] OR Guadeloup*[ad] OR Guadeloup*[tiab] OR Guadeloup*[pl] OR Guiana*[ad] OR Guiana*[tiab] OR French Guiana[pl] OR Guyan*[ad] OR Guyan*[tiab] OR Guyana[pl] OR Paraguay*[ad] OR Paraguay*[tiab] OR Paraguay[pl] OR Peru*[ad] OR Peru*[tiab] OR Peru[pl] OR Surinam*[ad] OR Surinam*[tiab] OR Surinam*[pl] OR Uruguay*[ad] OR Uruguay*[tiab] OR Uruguay[pl] OR Venez*[ad] OR Venez*[tiab] OR Venezuela[pl] OR Belize*[ad] OR Belize*[tiab] OR Belize[pl] OR Costa Ric*[ad] OR Costarric*[ad] OR Costaric*[ad] OR Costa Ric*[tiab] OR Costarric*[tiab] OR Costaric*[tiab] OR Costa Rica[pl] OR Salvador*[ad] OR Salvador*[tiab] OR El Salvador[pl] OR Guatemal*[ad] OR Guatemal*[tiab] OR Guatemala[pl] OR Hondur*[ad] OR Hondur*[tiab] OR Honduras[pl] OR Martinique[ad] OR Martiniqu*[tiab] OR Martinique[pl] OR Nicaragu*[ad] OR Nicaragu*[tiab] OR Nicaragua[pl] OR Panam*[ad] OR Panam*[tiab] OR Panama[pl] OR Mexico[Mesh] OR Mexic*[ad] OR Mexic*[tiab] OR Mejic*[tiab] OR Mexico[pl] OR Montserrat[ad] OR Montserrat*[tiab] OR Montserrat[pl] OR Baham*[ad] OR Baham*[tiab] OR Bahamas[pl] OR Cuba*[ad] OR Cuba*[tiab] OR Cuba[pl] OR Dominic*[ad] OR Dominic*[tiab] OR Dominican Republic[pl] OR Haiti*[ad] OR Haiti*[tiab] OR Haiti[pl] OR Jamaic*[ad] OR Jamaic*[tiab] OR Jamaica[pl] OR Puerto Rico[Mesh] OR Puerto Ric*[tiab] OR Puertorric*[tiab] OR Puertoric*[tiab] OR Saint Kitts[ad] OR Saint Kitts[tiab] OR Saint Kitts[pl] OR "Trinidad and Tobago"[tiab] OR "Trinidad and Tobago"[ad] OR "Trinidad and Tobago"[pl]) |
| #7 | #1 OR #2 OR #3 OR #4 OR #5 OR #6 |
| #6 | Respiratory Syncytial[tiab] |
| #5 | RSV[tiab] |
| #4 | Syncytial Virus*[tiab] |
| #3 | Respiratory Syncytial Virus Vaccines[Mesh] |
| #2 | Respiratory Syncytial Virus Infections[Mesh] |
| #1 | Respiratory Syncytial Virus, Human[Mesh] |

1. **EMBase (OVID)**

Date of search: 04/01/2023

Embase Classic+Embase <1947 to 2022 December 30>

| # | Query |
| --- | --- |
| 1 | exp Human respiratory syncytial virus/ |
| 2 | exp respiratory syncytial virus infection/ |
| 3 | exp respiratory syncytial virus vaccine/ |
| 4 | (Syncytial adj3 Virus*).ti,ab. |
| 5 | RSV.ti,ab. |
| 6 | (Respiratory adj1 Syncytial).ti,ab. |
| 7 | or/1-6 30964 |
| 8 | exp Latin America/ or exp South America/ or exp Central America/ or (Latin adj1 America*).ti,ab. or Latinamerica*.ti,ab. or Latinoamerica*.ti,ab. or Hispanoamerica.ti,ab. or Iberoamerica*.ti,ab. or (Ibero adj1 Americ*).ti,ab. or Panamerica*.ti,ab. or (South adj1 America*).ti,ab. or Southamerica*.ti,ab. or Sudamerica*.ti,ab. or (America adj1 Sur).ti,ab. or (Central adj1 America*).ti,ab. or Centroamerica*.ti,ab. or Mesoamerica*.ti,ab. or (Meso adj1 America*).ti,ab. or (Middle adj1 America*).ti,ab. or exp Caribbean Islands/ or Caribbean*.ti,ab. or Caribe*.ti,ab. or (West adj1 Indi*).ti,ab. or Antill*.ti,ab. or exp American indian/ or Amerindian*.ti,ab. or Indians.ti,ab. or (Native adj1 America*).ti,ab. or Patagoni*.ti,ab. or Andes.ti,ab. or Andean*.ti,ab. or Amazon*.ti,ab. or exp Argentina/ or Argentin*.ti,ab. or exp Bolivia/ or Bolivia*.ti,ab. or exp Brazil/ or Brazil*.ti,ab. or Brasil*.ti,ab. or exp Colombia/ or Colombia*.ti,ab. or exp Chile/ or Chile*.ti,ab. or exp Ecuador/ or Ecuador*.ti,ab. or exp French Guiana/ or Guiana*.ti,ab. or exp Guyana/ or Guyan*.ti,ab. or exp Paraguay/ or Paraguay*.ti,ab. or exp Peru/ or Peru*.ti,ab. or exp Suriname/ or Surinam*.ti,ab. or exp Uruguay/ or Uruguay*.ti,ab. or exp Venezuela/ or Venez*.ti,ab. or exp Belize/ or Beliz*.ti,ab. or exp Costa Rica/ or (Costa adj1 Rica).ti,ab. or Costarric*.ti,ab. or Costaric*.ti,ab. or exp El salvador/ or Salvador*.ti,ab. or exp Guatemala/ or Guatemal*.ti,ab. or exp Honduras/ or Hondur*.ti,ab. or exp Nicaragua/ or Nicaragu*.ti,ab. or exp Panama/ or Panam*.ti,ab. or exp Mexico/ or Mexic*.ti,ab. or exp Cuba/ or Cuba*.ti,ab. or exp Dominican Republic/ or Dominica*.ti,ab. or exp Haiti/ or Haiti*.ti,ab. or exp Jamaica/ or Jamaic*.ti,ab. or exp Puerto Rico/ or (Puerto adj1 Ric*).ti,ab. or Puertoric*.ti,ab. or Puertorric*.ti,ab. |
| 9 | 7 and 8 |
| 10 | limit 9 to yr="2012 -Current" |

1. **LILACS (BVS Eng)**

Date of search: 04/01/2023

| Database: | LILACS |
| --- | --- |
| Search on: | MH Respiratory Syncytial Virus, Human OR MH Respiratory Syncytial Virus Infections OR MH Respiratory Syncytial Virus Vaccines OR Syncytial OR Sincicial OR Sincitial OR RSV OR VSR OR VRS [Words] and 2012 OR 2013 OR 2014 OR 2015 OR 2016 OR 2017 OR 2018 OR 2019 OR 2020 OR 2021 OR 2022 [Country, year publication] |

1. **CINAHL Complete (EBSCO)**

Date of search: 04/01/2023

| # | Query |
| --- | --- |
| S10 | S6 AND S9 Limiters-Publish Date: 20120101-20230131 |
| S9 | S7 OR S8 |
| S8 | AB (Latin N1 America*) OR Latinamerica* OR Latinoamerica* OR Latin* OR Hispanic Americans OR Iberoamerica* OR (Ibero N1 Americ*) OR Panamerican* OR (Central N1 America*) OR Centroamerica* OR Mesoamerica* OR (Meso N1 America*) OR (Middle N1 America*) OR (South N1 America*) OR Southamerica* OR Sudamerica* OR (America N1 Sur) OR Caribbean OR Caribe* OR (West N1 Indi*) OR Antill* OR Amerindian* OR Indians OR (American N1 Indian*) OR (Native N1 America*) OR Patagoni* OR Andes OR Andean* OR Amazon* OR Argentin* OR Bolivia* OR Brazil* OR Brasil* Colombia* OR Colombia* OR Colombia OR Chile* OR Ecuador* OR Guiana* OR Guyan* OR Guyan* OR Paraguay* OR Paraguay* OR Peru* OR Surinam* OR Surinam* OR Uruguay* OR Venez* OR Belize* OR (Costa N1 Ric*) OR Costarric* OR Costaric* OR Costa Ric* OR Costarric* OR Salvador* OR Salvador* OR Guatemal* OR Guatemal* OR Guatemala OR Hondur* OR Nicaragu* OR Panam* OR Mexic* OR Cuba* OR Dominic* OR Dominic* OR Haiti* OR Jamaic* OR (Puerto N1 Ric*) OR Puertorric* OR Puertoric* |
| S7 | TI (Latin N1 America*) OR Latinamerica* OR Latinoamerica* OR Latin* OR Hispanic Americans OR Iberoamerica* OR (Ibero N1 Americ*) OR Panamerican* OR (Central N1 America*) OR Centroamerica* OR Mesoamerica* OR (Meso N1 America*) OR (Middle N1 America*) OR (South N1 America*) OR Southamerica* OR Sudamerica* OR (America N1 Sur) OR Caribbean OR Caribe* OR (West N1 Indi*) OR Antill* OR Amerindian* OR Indians OR (American N1 Indian*) OR (Native N1 America*) OR Patagoni* OR Andes OR Andean* OR Amazon* OR Argentin* OR Bolivia* OR Brazil* OR Brasil* Colombia* OR Colombia* OR Colombia OR Chile* OR Ecuador* OR Guiana* OR Guyan* OR Guyan* OR Paraguay* OR Paraguay* OR Peru* OR Surinam* OR Surinam* OR Uruguay* OR Venez* OR Belize* OR (Costa N1 Ric*) OR Costarric* OR Costaric* OR Costa Ric* OR Costarric* OR Salvador* OR Salvador* OR Guatemal* OR Guatemal* OR Guatemala OR Hondur* OR Nicaragu* OR Panam* OR Mexic* OR Cuba* OR Dominic* OR Dominic* OR Haiti* OR Jamaic* OR (Puerto N1 Ric*) OR Puertorric* OR Puertoric* |
| S6 | S1 OR S2 OR S3 OR S4 OR S5 |
| S5 | TI (Respiratory N1 Syncytial) OR AB (Respiratory N1 Syncytial) |
| S4 | TI RSV OR AB RSV |
| S3 | TI (Syncytial N3 Virus*) OR AB (Syncytial N3 Virus*) |
| S2 | (MH "Respiratory Syncytial Virus Infections") |
| S1 | (MH "Respiratory Syncytial Viruses") |

1. **Cochrane Library**

Date of search: 04/01/2023

| ID | Search |
| --- | --- |
| #1 | MeSH descriptor: [Respiratory Syncytial Virus, Human] explode all trees |
| #2 | MeSH descriptor: [Respiratory Syncytial Virus Infections] explode all trees |
| #3 | MeSH descriptor: [Respiratory Syncytial Virus Vaccines] explode all trees |
| #4 | (Syncytial NEAR/3 Virus*):ti,ab,kw |
| #5 | RSV:ti,ab,kw |
| #6 | (Respiratory NEAR/1 Syncytial):ti,ab,kw |
| #7 | #1 OR #2 OR #3 OR #4 OR #5 OR #6 |
| #8 | MeSH descriptor: [Latin America] explode all trees |
| #9 | MeSH descriptor: [Central America] explode all trees |
| #10 | MeSH descriptor: [South America] explode all trees |
| #11 | MeSH descriptor: [Caribbean Region] explode all trees |
| #12 | MeSH descriptor: [West Indies] explode all trees |
| #13 | MeSH descriptor: [Indians, South American] explode all trees |
| #14 | MeSH descriptor: [Indians, Central American] explode all trees |
| #15 | MeSH descriptor: [Mexico] explode all trees |
| #16 | MeSH descriptor: [Puerto Rico] explode all trees |
| #17 | ((Latin NEAR/1 America*) OR Latinamerica* OR Latinoamerica* OR Latin* OR Hispanic Americans OR Iberoamerica* OR (Ibero NEAR/1 Americ*) OR Panamerican* OR (Central NEAR/1 America*) OR Centroamerica* OR Mesoamerica* OR (Meso NEAR/1 America*) OR (Middle NEAR/1 America*) OR (South NEAR/1 America*) OR Southamerica* OR Sudamerica* OR (America NEAR/1 Sur) OR Caribbean OR Caribe* OR (West NEAR/1 Indi*) OR Antill* OR Amerindian* OR Indians OR (American NEAR/1 Indian*) OR (Native NEAR/1 America*) OR Patagoni* OR Andes OR Andean* OR Amazon* OR Argentin* OR Bolivia* OR Brazil* OR Brasil* Colombia* OR Colombia* OR Colombia OR Chile* OR Ecuador* OR Guiana* OR Guyan* OR Guyan* OR Paraguay* OR Paraguay* OR Peru* OR Surinam* OR Surinam* OR Uruguay* OR Venez* OR Belize* OR (Costa NEAR/1 Ric*) OR Costarric* OR Costaric* OR Costa Ric* OR Costarric* OR Salvador* OR Salvador* OR Guatemal* OR Guatemal* OR Guatemala OR Hondur* OR Nicaragu* OR Panam* OR Mexic* OR Cuba* OR Dominic* OR Dominic* OR Haiti* OR Jamaic* OR (Puerto NEAR/1 Ric*) OR Puertorric* OR Puertoric*):ti,ab,kw |
| #18 | #8 OR #9 OR #10 OR #11 OR #12 OR #13 OR #14 OR #15 OR #16 OR #17 |
| #19 | #7 AND #18 with Publication Year from 2022 to 2023, with Cochrane Library publication date Between Nov 2022 and Jan 2023, in Trials |

1. **EconLIT (Ovid)**

Date of search: 04/01/2023

Econlit <1886 to December 22, 2022>

| # | Query |
| --- | --- |
| 1 | (Syncytial adj3 Virus*).ti,ab. |
| 2 | RSV.ti,ab. |
| 3 | (Respiratory adj1 Syncytial).ti,ab. |
| 4 | 1 or 2 or 3 |
| 5 | ((Latin adj1 America*) or Latinamerica* or Latinoamerica* or Hispanoamerica or Iberoamerica* or (Ibero adj1 Americ*) or Panamerica* or (South adj1 America*) or Southamerica* or Sudamerica* or (America adj1 Sur) or (Central adj1 America*) or Centroamerica* or Mesoamerica* or (Meso adj1 America*) or (Middle adj1 America*) or Caribbean* or Caribe* or (West adj1 Indi*) or Antill* or Amerindian* or Indians or (Native adj1 America*) or Patagoni* or Andes or Andean* or Amazon* or Argentin* or Bolivia* or Brazil* or Brasil* or Colombia* or Chile* or Ecuador* or Guiana* or Guyan* or Paraguay* or Peru* or Surinam* or Uruguay* or Venez* or Beliz* or (Costa adj1 Rica) or Costarric* or Costaric* or Salvador* or Guatemal* or Hondur* or Nicaragu* or Panam* or Mexic* or Cuba* or Dominica* or Haiti* or Jamaic* or (Puerto adj1 Ric*) or Puertoric* or Puertorric*).ti,ab. |
| 6 | 4 and 5 |

1. **Web of Science**

Date of search: 04/01/2023

| Web of Science Core Collection for: |
| --- |
| (TS=Respiratory Syncytial Virus, Human OR TS=Respiratory Syncytial Virus Infections OR TS=Respiratory Syncytial Virus Vaccines OR TI=(Syncytial NEAR/3 Virus*) OR AB=(Syncytial NEAR/3 Virus*) OR TI=RSV OR AB=RSV OR TI=(Respiratory NEAR/1 Syncytial) OR AB=(Respiratory NEAR/1 Syncytial)) AND (TS=Latin America OR TI=(Latin NEAR/1 America*) OR AB=(Latin NEAR/1 America*) OR ALL=Latinamerica* OR ALL=Latinoamerica* OR ALL=Hispanoamerica* OR ALL=Iberoamerica* OR TI=(Ibero NEAR/1 America*) OR AB=(Ibero NEAR/1 America*) OR ALL=Panamerican* OR TS=Central America OR TI=(Central NEAR/1 America*) OR AB=(Central NEAR/1 America*) OR ALL=Centroamerica* OR ALL=Mesoamerica* OR TI=(Meso NEAR/1 America*) OR AB=(Meso NEAR/1 America*) OR TI=(Middle NEAR/1 America*) OR AB=(Middle NEAR/1 America*) OR TS=South America OR TI=(South NEAR/1 America*) OR AB=(South NEAR/1 America*) OR ALL=Southamerica* OR ALL=Sudamerica* OR TI=(America NEAR/1 Sur) OR AB=(America NEAR/1 Sur) OR TS=Caribbean Region OR ALL=Caribbean OR ALL=Caribe* OR TS=West Indies OR TI=(West NEAR/1 Indi*) OR AB=(West NEAR/1 Indi*) OR ALL=Antill* OR TS=Indians, South American OR TS=Indians, Central American OR ALL=Amerindian* OR TI=(America* NEAR/3 Indian*) OR AB=(America* NEAR/3 Indian*) OR TI=(Native NEAR/1 America*) OR AB=(Native NEAR/1 America*) OR ALL=Patagoni* OR ALL=Andes OR ALL=Andean* OR ALL=Amazon* OR ALL=Anguill* OR TI=(Antigua NEAR/1 Barbuda) OR AB=(Antigua NEAR/1 Barbuda) OR ALL=Argentin* OR ALL=Baham* OR ALL=Bermud* OR ALL=Bolivia* OR ALL=Brazil* OR ALL=Brasil* OR ALL=Cayman* OR ALL=Curaçao OR ALL=Colombia* OR ALL=Chile* OR ALL= Ecuador* OR ALL=Grenad* OR ALL=Guadeloup* OR ALL=Guiana* OR ALL=Guyan* OR ALL=Paraguay* OR ALL=Peru* OR ALL=Surinam* OR ALL=Uruguay* OR ALL=Venez* OR ALL=Belize* OR TI=(Costa NEAR/1 Ric*) OR AB=(Costa NEAR/1 Ric*) OR ALL=Costarric* OR ALL=Costaric* OR ALL=Salvador* OR ALL=Guatemal* OR ALL=Hondur* OR ALL=Martiniqu* OR ALL=Nicaragu* OR ALL=Panam* OR TS=Mexico OR ALL=Mexic* OR ALL=Montserrat* OR ALL=Cuba* OR ALL=Dominic* OR ALL=Haiti* OR ALL=Jamaic* OR TS=Puerto Rico OR TI=(Puerto NEAR/1 Ric*) OR AB=(Puerto NEAR/1 Ric*) OR ALL=Puertorric* OR TI=(Saint NEAR/1 Kitts) OR AB=(Saint NEAR/1 Kitts) OR TI=(Trinidad NEAR/1 Tobago) OR AB=(Trinidad NEAR/1 Tobago)) AND (PY=2022 OR PY=2023) |

# Grey literature sources

IDWEEK (<https://idweek.org/>)

ISPPD (<https://isppd.kenes.com/>)

ISRIV (<https://isirv.org/site/>)

RESVINET (<https://www.resvinet.org/>)

SLIPE ([https://slipe.org/web/previous-events/](https://slipe.org/web/eventos-anteriores/))

API (<https://www.apiinfectologia.org/>)

BVSalud ([https://bvsalud.org/es/)](https://bvsalud.org/es/)%203)

# Supplementary Table 1. Characteristics of the included studies

| **Author, year** | **Country** | **Study**  **start-end** | **Study design** | **Age group** | **Population**  **risk** | **Sample**  **size** |
| --- | --- | --- | --- | --- | --- | --- |
| Acuña 2022* (1) | Argentina | 01/18-12/12 | Case series | Pediatric | NR | 584 |
| Andino 2017* (2) | Argentina | 01/16-10/16 | Cross-sectional^S^ | NR | NR | 1,672 |
| Atwell 2016* (3) | Argentina | 05/13-10/13 | Case series | Pediatric | Normal | 846 |
| Barbaro 2020* (4) | Argentina | 06/15-09/18 | Case series | Pediatric | NR | 938 |
| Baumeister 2019* (5) | Argentina | 01/12-12/16 | Cross-sectional^S^ | All | NR | 346,962 |
| Caballero 2019* (6) | Argentina | 04/14-12/16 | Cross-sectional | Pediatric | Normal | 114 |
| Castelo 2017* (7) | Argentina | 06/14-07/15 | Cross-sectional | Pediatric | NR | 1,296 |
| Cook 2019* (8) | Argentina | 04/18-02/19 | Case series | Adults | High | 124 |
| Ferolla 2019* (9) | Argentina | 01/12-12/13 | Case series | Pediatric | NR | 622 |
| Ferolla 2022* (10) | Argentina | 01/17-12/19 | Case series | Pediatric | Normal | 125 |
| Ferrero 2022* (11) | Argentina | 01/20-12/21 | Case series | Pediatric | NR | NR |
| Holgado 2020* (12) | Argentina | 04/17-12/19 | Case series | Pediatric | Normal | 182 |
| Leone 2022* (13) | Argentina | 08/21-09/21 | Cross-sectional | Pediatric | Normal | 344 |
| López 2020* (14) | Argentina | 04/17-08/18 | Case series | Pediatric | Normal | 75 |
| Lucion 2014* (15) | Argentina | 03/00-11/13# | Cross-sectional | Pediatric | NR | 560 |
| Orqueda 2022* (16) | Argentina | 08/21-10/21 | Cross-sectional | Pediatric | Normal | 619 |
| Pérez 2014* (17) | Argentina | 04/12-03/13 | Cross-sectional | Pediatric | Both | 305 |
| Rojo 2017* (18) | Argentina | 06/14-07/15 | Cross-sectional | Pediatric | NR | 1,256 |
| Russo 2018** (19) | Argentina | 01/17-10/17 | Cross-sectional^S^ | All | NR | 520 |
| Viegas 2016* (20) | Argentina | 01/12-12/14 | Case series | Pediatric | NR | 97 |
| Zuázaga 2020* (21) | Argentina | 08/17-07/19 | Cross-sectional | Pediatric | Normal | 145 |
| Chavez 2019* (22) | Bolivia | 01/12-12/17 | Cross-sectional^S^ | All | NR | 2,606 |
| Gareca Perales 2020* (23) | Bolivia | 03/16-03/17 | Cross-sectional | Pediatric | NR | 274 |
| Alvarez 2018* (24) | Brazil | 01/13-12/14 | Cross-sectional | Pediatric | Normal | 181 |
| Araujo Castro 2020* (25) | Brazil | 09/12-08/13 | Cross-sectional | Pediatric | NR | 164 |
| Azevedo 2022** (26) | Brazil | 05/20-11/20 | Cross-sectional | Adults | NR | 156 |
| Bamberg 2021* (27) | Brazil | 01/19-12/20 | Cross-sectional | Pediatric | NR | 226 |
| Bastos 2022* (28) | Brazil | 03/13-09/14 | Cross-sectional | Pediatric | Normal | 173 |
| Boschiero 2022* (29) | Brazil | NR | Cross-sectional | All | NR | 6,235 |
| Camargo Costa 2018* (30) | Brazil | 06/12-08/13 | Cross-sectional | Pediatric | Normal | 153 |
| Cesar 2018** (31) | Brazil | 09/16-07/17 | Case series | Pediatric | NR | 62 |
| De Paulis 2021* (32) | Brazil | 01/13-11/15 | Cross-sectional | Pediatric | NR | 70 |
| Faggion 2019* (33) | Brazil | 01/12-12/13 | Cross-sectional | All | NR | 775 |
| Ferlini 2016* (34) | Brazil | 01/12-09/14 | Cross-sectional | Pediatric | Normal | 66 |
| Freitas 2019** (35) | Brazil | 01/13-06/17 | Cross-sectional^S^ | Pediatric | High | 69 |
| Furlan 2016*** (36) | Brazil | 05/12-12/13 | Cross-sectional | Adults | High | 151 |
| Giamberardino 2022* (37) | Brazil | 03/17-09/18 | Cross-sectional | Pediatric | NR | 468 |
| Gurgel 2016* (38) | Brazil | 04/13-03/13 | Cross-sectional | Pediatric | Both | 507 |
| Holtman Ferreira 2022* (39) | Brazil | 04/19-09/19 | Cross-sectional | Pediatric | High | 279 |
| Lomar 2017** (40) | Brazil | 01/15-12/15 | Cross-sectional | NR | NR | 949 |
| Medeiros 2022* (41) | Brazil | 02/15-08/16 | Case series | Pediatric | NR | 399 |
| Mendes 2021* (42) | Brazil | 01/13-12/18 | Case series | Pediatric | Normal | 220 |
| Moreira 2017* (43) | Brazil | 07/11-05/13# | Case series | Pediatric | Normal | 147 |
| Neves 2020* (44) | Brazil | 11/12-10/14 | Cross-sectional | Pediatric | Normal | 120 |
| Ocadaque 2015*** (45) | Brazil | 01/13-12/14 | Cross-sectional | Pediatric | Normal | 542 |
| Pinheiro Lopes 2020* (46) | Brazil | 04/18-03/19 | Cross-sectional | Pediatric | High | 151 |
| Piva 2014** (47) | Brazil | 01/12-12/13 | Case series | Pediatric | NR | 36 |
| Pucarelli-Lebreiro 2022* (48) | Brazil | 04/20-04/21 | Cross-sectional | NR | NR | 136 |
| Rodrigues de Araujo 2020* (49) | Brazil | 01/13-12/18 | Cross-sectional^S^ | All | NR | 4,832 |
| Santos 2021* (50) | Brazil | 01/14-06/17 | Cross-sectional | Pediatric | NR | 387 |
| Serra Campelo 2022* (51) | Brazil | 01/13-12/14 | Cross-sectional | Pediatric | Normal | 545 |
| Silva 2017** (52) | Brazil | 01/16-12/16 | Cross-sectional | Adults | High | 312 |
| Sobrinho 2021* (53) | Brazil | 04/17-08/18 | Cross-sectional | Pediatric | Normal | 146 |
| Stein 202* (54) | Brazil | 06/20-09/20 | Cross-sectional | All | NR | 1,435 |
| Tabor 2021* (55) | Brazil | 11/17-10/18 | Case series | Pediatric | NR | 64 |
| Varela 2022* (56) | Brazil | 05/20-11/20 | Cross-sectional | Pediatric | Normal | 436 |
| Vianna 2021* (57) | Brazil | 03/16-12/18 | Cross-sectional^S^ | Pediatric | NR | 632 |
| Vieira 2017* (58) | Brazil | 01/13-12/15 | Cross-sectional | Pediatric | NR | 94 |
| Watanabe 2022* (59) | Brazil | 03/19-08/19 | Case series | Pediatric | Normal | 38 |
| Wollmeister 2018* (60) | Brazil | 04/14-09/14 | Cross-sectional | Pediatric | Normal | 172 |
| Yamaguto 2022* (61) | Brazil | 01/13-12/19 | Cross-sectional | All | Both | 161 |
| Arias-Bravo 2020* (62) | Chile | 05/16-08/17 | Cross-sectional | Pediatric | Normal | 124 |
| Bertrand 2022* (63) | Chile | 05/15-09/15 | Cross-sectional | Pediatric | Normal | 98 |
| Corvalán 2019* (64) | Chile | 06/17-07/17 | Cross-sectional | Pediatric | NR | 47 |
| Espinosa 2017* (65) | Chile | 01/13-12/14 | Cross-sectional | Pediatric | Normal | 90 |
| Diaz 2015b** (66) | Chile | 01/12-12/15 | Cross-sectional | Pediatric | NR | 76 |
| Fica 2019* (67) | Chile | 01/15-12/15 | Cross-sectional^S^ | Adults | High | 132 |
| Le Corre 2021* (68) | Chile | 06/14-10/14 | Cross-sectional | Pediatric | NR | 57 |
| Leiva 2021* (69) | Chile | 01/14-12/16 | Case series | Pediatric | High | 94 |
| Puchi 2014* (70) | Chile | 01/12-11/13 | Cross-sectional | Pediatric | NR | 5,126 |
| Riquelme 2014* (71) | Chile | 05/12-09/12 | Cross-sectional | Adults | NR | 128 |
| Saldías Peñafiel 2016* (72) | Chile | 06/14-12/15 | Cross-sectional | Adults | High | 240 |
| Santolaya 2017* (73) | Chile | 07/12-12/15 | Cross-sectional | Pediatric | High | 176 |
| Valencia Sapunar 2021* (74) | Chile | 01/15-12/20 | Cross-sectional | All | NR | 269,174 |
| Yañez 2017* (75) | Chile | 01/16-12/16 | Cross-sectional^S^ | Pediatric | Normal | 1,856 |
| Zepeda 2018* (76) | Chile | 07/15-12/15 | Case series | Pediatric | Normal | 260 |
| Acuña Cordero 2018** (77) | Colombia | 01/13-02/14 | Cross-sectional | NR | Normal | 240 |
| Bettin-Martinez 2019* (78) | Colombia | 04/16-07/17 | Case series | Pediatric | NR | 304 |
| Buendía 2021a* (79) | Colombia | 01/19-12/19 | Case series | Pediatric | Normal | 417 |
| Buendía 2021b* (80) | Colombia | 01/15-12/16 | Case series | Pediatric | Normal | 417 |
| Buendía 2022* (81) | Colombia | 12/16-12/18 | Cross-sectional | Pediatric | Normal | 200 |
| Camacho-Cruz 2021* (82) | Colombia | 11/17-05/18 | Cross-sectional | Pediatric | NR | 17 |
| Casallas Vega 2021* (83) | Colombia | 07/16-06/17 | Cross-sectional | Pediatric | Normal | 42 |
| Fernandez-Sarmiento 2022* (84) | Colombia | 10/15-12/19 | Cross-sectional | Pediatric | NR | 645 |
| García-Corzo 2017* (85) | Colombia | 12/12-11/13 | Cross-sectional | Pediatric | Normal | 215 |
| Londono-Avendano 2021* (86) | Colombia | 01/14-12/18 | Cross-sectional^S^ | Pediatric | NR | 2,148 |
| Lozano-Espinosa 2020* (87) | Colombia | 08/17-06/18 | Case series | Pediatric | High | 217 |
| Pedraza-Bernal 2016* (88) | Colombia | 01/14-01/15 | Cross-sectional | Pediatric | Normal | 416 |
| Piña-Hincapie 2020* (89) | Colombia | 03/14-08/15 | Cross-sectional | Pediatric | High | 1,365 |
| Preciado 2015* (90) | Colombia | 04/13-05/14 | Cross-sectional | Pediatric | Normal | 127 |
| Rodriguez-Martinez 2018* (91) | Colombia | 03/15-04/16 | Cross-sectional | Pediatric | Normal | 303 |
| Rodriguez-Martinez 2020* (92) | Colombia | 01/16-12/17 | Case series | Pediatric | NR | 89 |
| Rodriguez-Martinez 2022* (93) | Colombia | 01/18-12/18 | Case series | Pediatric | Normal | 1,215 |
| Ruiz-Ponce de León2021* (94) | Colombia | 03/20-11/20 | Cross-sectional | Pediatric | NR | 113 |
| Suescún-Carnero 2021* (95) | Colombia | 01/16-12/17 | Cross-sectional | All | NR | 614 |
| Vela Avendaño 2018*** (96) | Colombia | 01/16-12/16 | Cross-sectional | Pediatric | NR | 322 |
| Villamil 2019** (97) | Colombia | 09/17-03/18 | Cross-sectional | Pediatric | NR | 213 |
| Zarur-Torralvo 2022* (98) | Colombia | 01/16-12/21 | Cross-sectional^S^ | Pediatric | Normal | 1,480 |
| Fonseca 2019** (99) | Costa Rica | 08/14-09/14 | Cross-sectional | Pediatric | NR | 59 |
| Gordillo Hernández 2018* (100) | Cuba | 05/12-06/13 | Cross-sectional^S^ | All | NR | 1,604 |
| Macia Quintosa 2021* (101) | Cuba | 01/14-12/16 | Cross-sectional | Pediatric | Normal | 171 |
| Martínez Muné 2018* (102) | Cuba | 09/13-04/14 | Cross-sectional | All | NR | 2,241 |
| Azziz-Baumgartner 2022a**** (103) | Ecuador | 01/12-12/17 | Cross-sectional^S^ | All | NR | 30,574 |
| Castro Ortega 2019* (104) | Ecuador | 04/16-04/17 | Cross-sectional | Pediatric | High | 186 |
| Suryadevara 2022* (105) | Ecuador | 07/18-03/20 | Cross-sectional | Pediatric | NR | 820 |
| Aragón Montenegro 2018*** (106) | El Salvador | 01/14-12/15 | Cross-sectional | Pediatric | Both | 81 |
| López de Orellana 2017*** (107) | El Salvador | 01/14-12/15 | Case series | Pediatric | Normal | 132 |
| Rivera Macías 2016*** (108) | El Salvador | 01/12-12/13 | Cross-sectional^S^ | Pediatric | Both | 115 |
| Canesson 2021* (109) | French Guiana | 01/15-12/17 | Cross-sectional | Pediatric | Normal | 415 |
| Gatica 2014** (110) | Guatemala | 01/13-09/13 | Case series | Pediatric | NR | 157 |
| Milucky 2020* (111) | Guatemala | 10/13-09/15 | Cross-sectional | Adults | High | 304 |
| Gassant 2022** (112) | Haiti | 12/19-01/22 | Cross-sectional | Pediatric | NR | 143 |
| Ampuero 2019** (113) | LAC(113) | 07/16-03/19 | Cross-sectional | NR | NR | 2,562 |
| Azziz-Baumgartner 2022b* (114) | LAC | 12/14-11/17 | Prospective cohort | Pediatric | Normal | 1,567 |
| Chadha 2020* (115) | LAC | 01/17-04/19 | Cross-sectional^S^ | All | NR | 3,550 |
| Dawood 2015* (116) | LAC | 09/12-10/13 | Cross-sectional | Pediatric | NR | 703 |
| Langley 2022* (117) | LAC | 12/13-10/17 | Prospective cohort | Pediatric | NR | 430 |
| Obando-Pacheco 2018* (118) | LAC | 01/12-12/17 | Cross-sectional^S^ | All | NR | NR |
| Staadegaard 2021* (119) | LAC | 01/12-12/18 | Cross-sectional^S^ | All | NR | NR |
| Vasquez-Hoyos 2021a* (120) | LAC | 05/17-10/19 | Cross-sectional | Pediatric | Both | 5,297 |
| Vasquez-Hoyos 2021b** (121) | LAC | 01/20-08/20 | Cross-sectional | Pediatric | NR | 234 |
| Benítez-Guerra 2020* (122) | Mexico | 01/14-12/19 | Prospective cohort | Pediatric | High | 294 |
| Diaz 2015a* (123) | Mexico | 12/12-04/13 | Cross-sectional | Pediatric | NR | 162 |
| Díaz-Chiguer 2019* (124) | Mexico | 08/16-03/17 | Cross-sectional | Adults | NR | 22 |
| Fernandes-Matano 2017* (125) | Mexico | 10/14-09/15 | Cross-sectional | All | Normal | 872 |
| González-Ortiz 2019* (126) | Mexico | 11/12-12/15 | Cross-sectional | Pediatric | Normal | 1,252 |
| McCarthy 2019* (127) | Mexico | 03/14-02/17 | Case series | All | Normal | 257 |
| Quezada 2020* (128) | Mexico | 01/18-12/19 | Cross-sectional | Pediatric | NR | 297 |
| Robledo-Aceves 2018* (129) | Mexico | 09/12-01/15 | Cross-sectional | Pediatric | NR | 134 |
| Núñez-Samudio 2021* (130) | Panama | 01/16-12/16 | Cross-sectional | Pediatric | Normal | 85 |
| Arias 2019* (131) | Paraguay | 01/17-10/18 | Cross-sectional | Pediatric | High | 65 |
| Becerra 2019* (132) | Peru | 04/12-03/14 | Cross-sectional | Pediatric | High | 117 |
| Chirinos-Saire 2021* (133) | Peru | 01/17-12/18 | Cross-sectional | Pediatric | High | 707 |
| Gómez de la Torre Pretell 2022* (134) | Peru | 03/22-04/22 | Cross-sectional | All | Normal | 342 |
| Huayta Meza 2022* (135) | Peru | 01/21-06/21 | Cross-sectional | Pediatric | NR | 2,341 |
| Pérez-Lazo 2021* (136) | Peru | 07/20-11/20 | Cross-sectional | Adults | Both | 295 |
| Ramírez-Soto 2022* (137) | Peru | 05/15-10/21 | Cross-sectional | All | NR | NR |
| Romero 2013** (138) | Peru | 08/12-03/13 | Cross-sectional | NR | NR | 356 |
| Romero 2019** (139) | Peru | 08/17-07/18 | Cross-sectional | Pediatric | NR | 489 |
| Tinoco 2019* (140) | Peru | 02/14-03/15 | Cross-sectional | Pediatric | NR | 258 |
| Hernández-Marquez 2021** (141) | Puerto Rico | NR | Case series | Pediatric | NR | 128 |
| Paz Bailey 2018** (142) | Puerto Rico | 05/12-12/17 | Cross-sectional | All | NR | 19,228 |
| Quandelacy 2022** (143) | Puerto Rico | 09/19-09/20 | Cross-sectional | NR | NR | 2,439 |
| Rivera-Sepulveda 2017* (144) | Puerto Rico | 01/10-09/14# | Cross-sectional | Pediatric | NR | 4,201 |
| Rodríguez-Santiago 2020** (145) | Puerto Rico | 01/16-12/19 | Cross-sectional | NR | NR | 20,270 |
| Grunberg 2021* (146) | Suriname | 05/16-04/18 | Cross-sectional | All | NR | 1,096 |
| Juliana 2021* (147) | Suriname | 04/12-03/14 | Cross-sectional | Pediatric | Normal | 316 |
| Machado 2016* (148) | Uruguay | 06/12-09/12 | Case series | Pediatric | Normal | 887 |
| Machado 2018* (149) | Uruguay | 06/14-09/14 | Case series | Pediatric | Normal | 742 |
| Morosini 2018* (150) | Uruguay | 01/13-12/16 | Cross-sectional | Pediatric | Normal | 650 |
| Pinchak 2019* (151) | Uruguay | 06/15-08/15 | Case series | Pediatric | Normal | 125 |
| Sánchez 2021* (152) | Uruguay | 01/20-05/20 | Cross-sectional | Pediatric | NR | 30 |

# Supplementary Table 2. Studies excluded at full text screening stage

| **Author, year** | **Reason for exclusion** |
| --- | --- |
| Astudillo-Molina 2018 (153) | Wrong patient population |
| Azziz-Baumgartner 2022 (103) | Wrong setting |
| Billard 2022 (154) | Not enough information |
| da Silva 2020 (155) | Wrong patient population |
| CONITEC 2012 (156) | Not enough information |
| Buendia 2021 (157) | Duplicate |
| Caballero 2021 (158) | Wrong patient population |
| Cardoso 2019 (159) | Wrong patient population |
| Cintra 2014 (160) | Duplicate |
| dos Santos Ferreira 2019 (161) | Wrong patient population |
| Fernandes 2014 (162) | Not enough information |
| Galvis 2022 (163) | Wrong patient population |
| García Corzo 2016 (164) | Wrong patient population |
| Gaytán-Morales 2021 (165) | Wrong patient population |
| Gentile 2019 (166) | Wrong setting |
| Sanchez 2021 (167) | Wrong patient population |
| Gonzalez Dambrauskas 2018 (168) | Duplicate |
| Hirve 2020 (115) | Not enough information |
| Jimenez 2020 (169) | Not enough information |
| Jurado 2014 (170) | Not enough information |
| Lucion 2014 (15) | Duplicate |
| Luisi 2020 (171) | Wrong setting |
| Machado 2018 (149) | Duplicate |
| Majer 2015 (172) | Duplicate |
| Meyer 2020 (173) | Wrong patient population |
| Morosini 2016 (150) | Duplicate |
| Moubarak 2019 (174) | Duplicate |
| Mould-Quevedo 2012 (175) | Duplicate |
| Myckietiuk 2019 (176) | Duplicate |
| Nader 2016 (177) | Wrong patient population |
| Oliveira 2019 (178) | Not enough information |
| Orqueda 2022 (16) | Duplicate |
| Paladio 2020 (179) | Not enough information |
| Perez-Lazo 2021 (136) | Duplicate |
| Piñeros 2021 (180) | Wrong patient population |
| Porfirio de Souza 2019 (181) | Wrong patient population |
| Romero-Espinoza 2018 (182) | Wrong outcome |
| Rosselli 2013 (183) | Not enough information |
| Rueda 2013 (184) | Not enough information |
| Seliem 2018 (185) | Wrong setting |
| Sinisterra 2017 (186) | Duplicate |
| Solis-Esquilin 2020 (187) | Wrong patient population |
| Tomashek 2013 (188) | Not enough information |
| Tobolowsky 2019 (189) | Wrong patient population |
| Villamil 2020 (190) | Wrong outcome |

# Risk of bias assessment instruments

## Supplementary Table 3. Risk of bias assessment for cohort and cross-sectional studies

| **Author, year** | **Evaluation *** | | | | | | | | | | | | | | |
| --- | --- | --- | --- | --- | --- | --- | --- | --- | --- | --- | --- | --- | --- | --- | --- |
|  | **1** | **2** | **3** | **4** | **5** | **6** | **7** | **8** | **9** | **10** | **11** | **12** | **13** | **14** | **Final** |
| Acuña Cordero 2018 (77) | Yes | Yes | CD | Yes | No | NA | NA | NA | Yes | No | Yes | NA | NA | NA | FAIR |
| Alvarez 2018 (24) | Yes | Yes | Yes | Yes | No | NA | NA | Yes | Yes | No | Yes | NA | NA | NA | GOOD |
| Ampuero 2019 (113) | Yes | Yes | CD | Yes | No | NA | NA | No | Yes | No | Yes | NA | NA | NA | FAIR |
| Andino 2017 (2) | Yes | Yes | Yes | Yes | No | NA | NA | No | Yes | No | Yes | NA | NA | NA | GOOD |
| Aragón Montenegro 2018 (106) | Yes | Yes | Yes | Yes | No | NA | NA | No | Yes | No | Yes | NA | NA | NA | GOOD |
| Araujo Castro 2020 (25) | Yes | Yes | CD | Yes | No | NA | NA | Yes | Yes | No | Yes | NA | NA | NA | FAIR |
| Arias 2019 (131) | Yes | Yes | Yes | Yes | No | NA | NA | No | Yes | No | Yes | NA | NA | NA | GOOD |
| Arias-Bravo 2020 (62) | Yes | Yes | Yes | Yes | No | NA | NA | Yes | Yes | No | Yes | NA | NA | NA | GOOD |
| Azevedo 2022 (26) | Yes | Yes | CD | Yes | No | NA | NA | No | Yes | No | Yes | NA | NA | NA | FAIR |
| Azziz-Baumgartner 2022a (103) | Yes | Yes | Yes | Yes | No | NA | NA | NA | Yes | Yes | Yes | NA | NA | NA | GOOD |
| Azziz-Baumgartner 2022b (114) | Yes | Yes | Yes | Yes | No | Yes | Yes | NA | Yes | Yes | Yes | NA | No | No | FAIR |
| Bamberg 2021 (27) | Yes | Yes | Yes | Yes | No | NA | NA | NA | CD | No | Yes | NA | NA | NA | FAIR |
| Bastos 2021 (28) | Yes | Yes | CD | Yes | No | NA | NA | Yes | Yes | No | Yes | NA | NA | NA | FAIR |
| Baumeister 2019 (5) | Yes | Yes | Yes | Yes | No | NA | NA | NA | Yes | No | Yes | NA | NA | NA | GOOD |
| Becerra 2019 (132) | Yes | Yes | Yes | Yes | No | NA | NA | No | Yes | No | Yes | NA | NA | NA | GOOD |
| Benítez-Guerra 2019 (122) | Yes | Yes | CD | Yes | No | Yes | Yes | NA | Yes | No | Yes | NA | Yes | Yes | FAIR |
| Bertrand 2022 (63) | Yes | Yes | CD | Yes | No | NA | NA | Yes | Yes | No | Yes | NA | NA | NA | FAIR |
| Boschiero 2022 (29) | Yes | No | CD | Yes | No | NA | NA | NA | Yes | No | Yes | NA | NA | NA | POOR |
| Buendia 2022 (81) | Yes | Yes | Yes | Yes | Yes | NA | NA | NA | Yes | No | Yes | NA | NA | NA | GOOD |
| Caballero 2019 (6) | Yes | Yes | Yes | Yes | No | NA | NA | Yes | Yes | No | Yes | NA | NA | NA | GOOD |
| Camacho-Cruz 2021 (82) | Yes | Yes | CD | Yes | Yes | NA | NA | No | Yes | No | Yes | NA | NA | NA | FAIR |
| Camargo Costa 2018 (30) | Yes | Yes | CD | Yes | No | NA | NA | NA | Yes | No | Yes | NA | NA | NA | FAIR |
| Canesson 2021 (109) | Yes | Yes | Yes | Yes | No | NA | NA | Yes | Yes | No | Yes | NA | NA | NA | GOOD |
| Casallas Vega 2021 (83) | Yes | Yes | CD | Yes | Yes | NA | NA | No | Yes | No | Yes | NA | NA | NA | FAIR |
| Castello 2017 (7) | Yes | Yes | CD | Yes | No | NA | NA | No | Yes | No | Yes | NA | NA | NA | FAIR |
| Castro Ortega 2019 (104) | Yes | Yes | Yes | Yes | No | NA | NA | NA | Yes | No | Yes | NA | NA | NA | GOOD |
| Chada 2020 (115) | Yes | Yes | Yes | Yes | No | NA | NA | NA | Yes | No | Yes | NA | NA | NA | GOOD |
| Chavez 2019 (22) | Yes | Yes | Yes | Yes | No | NA | NA | No | Yes | No | Yes | NA | NA | NA | GOOD |
| Chirinos-Saire 2019 (133) | Yes | Yes | Yes | Yes | No | NA | NA | NA | Yes | Yes | Yes | NA | NA | NA | GOOD |
| Corvalán 2019 (64) | Yes | Yes | CD | Yes | No | NA | NA | No | Yes | No | Yes | NA | NA | NA | FAIR |
| Dawood 2015 (116) | Yes | Yes | CD | Yes | No | NA | NA | No | Yes | No | Yes | NA | NA | NA | FAIR |
| De Paulis 2021 (32) | Yes | Yes | Yes | Yes | Yes | NA | NA | No | Yes | No | Yes | NA | NA | NA | GOOD |
| Diaz 2015a (123) | Yes | Yes | CD | Yes | No | NA | NA | No | Yes | No | Yes | NA | NA | NA | FAIR |
| Diaz 2015b (66) | Yes | Yes | CD | Yes | No | NA | NA | No | Yes | No | Yes | NA | NA | NA | FAIR |
| Díaz-Chiguer 2019 (124) | Yes | Yes | No | Yes | No | NA | NA | No | Yes | No | Yes | NA | NA | NA | FAIR |
| Espinosa 2017 (65) | Yes | Yes | CD | Yes | No | NA | NA | Yes | Yes | No | Yes | NA | NA | NA | FAIR |
| Faggion 2019 (33) | Yes | Yes | CD | Yes | No | NA | NA | No | Yes | No | Yes | NA | NA | NA | FAIR |
| Ferlini 2016 (34) | Yes | Yes | Yes | Yes | No | NA | NA | Yes | Yes | No | Yes | NA | NA | NA | GOOD |
| Fernandes-Matano 2017 (125) | Yes | No | Yes | Yes | No | NA | NA | NA | Yes | No | Yes | NA | NA | NA | FAIR |
| Fernandez-Sarmiento 2022 (84) | Yes | Yes | Yes | Yes | Yes | NA | NA | Yes | Yes | No | Yes | NA | NA | NA | GOOD |
| Fica 2019 (67) | Yes | Yes | Yes | Yes | No | NA | NA | No | Yes | No | Yes | NA | NA | NA | GOOD |
| Fonseca 2019 (99) | Yes | Yes | Yes | Yes | No | NA | NA | No | Yes | No | Yes | NA | NA | NA | GOOD |
| Freitas 2019 (35) | Yes | Yes | No | Yes | No | NA | NA | No | Yes | No | Yes | NA | NA | NA | FAIR |
| Furlan 2016 (36) | Yes | Yes | CD | Yes | Yes | NA | NA | No | Yes | No | Yes | NA | NA | NA | FAIR |
| García-Corzo 2017 (85) | Yes | Yes | CD | Yes | No | NA | NA | NA | Yes | No | Yes | NA | NA | NA | FAIR |
| Gareca Perales 2020 (23) | Yes | Yes | Yes | Yes | No | NA | NA | Yes | Yes | No | Yes | NA | NA | NA | GOOD |
| Gassant 2022 (112) | Yes | Yes | CD | Yes | No | NA | NA | Na | Yes | No | Yes | NA | NA | NA | FAIR |
| Giamberardino 2022 (37) | Yes | Yes | Yes | Yes | Yes | NA | NA | No | Yes | No | Yes | NA | NA | NA | GOOD |
| Gómez de la Torre Pretell 2022 (134) | Yes | Yes | Yes | Yes | No | NA | NA | No | Yes | No | Yes | NA | NA | NA | GOOD |
| González-Ortiz 2019 (126) | Yes | Yes | CD | Yes | No | NA | NA | Yes | Yes | No | Yes | NA | NA | NA | FAIR |
| Gordillo Hernández 2018 (100) | Yes | Yes | Yes | Yes | No | NA | NA | No | Yes | No | Yes | NA | NA | NA | GOOD |
| Grunberg 2021 (146) | Yes | Yes | Yes | Yes | No | NA | NA | No | Yes | No | Yes | NA | NA | NA | GOOD |
| Gurgel 2016 (38) | Yes | Yes | CD | Yes | Yes | NA | NA | NA | Yes | No | Yes | NA | NA | NA | FAIR |
| Holtman Ferreira 2022 (39) | Yes | Yes | CD | Yes | No | NA | NA | Yes | Yes | No | Yes | NA | NA | NA | FAIR |
| Huayta Meza 2022 (135) | Yes | No | CD | Yes | No | NA | NA | No | Yes | No | Yes | NA | NA | NA | POOR |
| Juliana 2021 (147) | Yes | Yes | Yes | Yes | No | NA | NA | Yes | Yes | No | Yes | NA | NA | NA | GOOD |
| Langley 2022 (117) | Yes | Yes | CD | Yes | Yes | Yes | Yes | NA | Yes | Yes | Yes | NA | Yes | No | POOR |
| Le Corre 2021 (68) | Yes | Yes | Yes | Yes | No | NA | NA | Yes | Yes | No | Yes | NA | NA | NA | GOOD |
| Leone 2022 (13) | Yes | Yes | CD | Yes | Yes | NA | NA | Yes | Yes | No | Yes | NA | NA | NA | FAIR |
| Lomar 2017 (40) | Yes | Yes | CD | Yes | No | NA | NA | No | Yes | No | Yes | NA | NA | NA | FAIR |
| Londono-Avendano 2021 (86) | Yes | Yes | Yes | Yes | No | NA | NA | No | Yes | No | Yes | NA | NA | NA | GOOD |
| Lucion 2014 (15) | Yes | Yes | Yes | Yes | No | NA | NA | No | Yes | No | Yes | NA | NA | NA | GOOD |
| Macia Quintosa 2021 (101) | Yes | Yes | CD | Yes | No | NA | NA | Yes | Yes | No | Yes | NA | NA | NA | FAIR |
| Martínez Muné 2018 (102) | Yes | No | CD | CD | No | NA | NA | No | Yes | No | Yes | NA | NA | NA | POOR |
| Milucky 2020 (111) | Yes | Yes | CD | Yes | Yes | NA | NA | No | Yes | No | Yes | NA | NA | NA | FAIR |
| Morosini 2018 (150) | Yes | Yes | Yes | Yes | No | NA | NA | NA | Yes | No | Yes | NA | NA | NA | GOOD |
| Neves 2020 (44) | Yes | Yes | Yes | Yes | No | NA | NA | No | Yes | No | Yes | NA | NA | NA | GOOD |
| Nuñez-Samudio 2021 (130) | Yes | Yes | CD | Yes | No | NA | NA | NA | Yes | No | Yes | NA | NA | NA | FAIR |
| Obando-Pacheco 2018 (118) | Yes | No | Yes | CD | No | NA | NA | NA | Yes | No | Yes | NA | NA | NA | FAIR |
| Ocadaque 2015 (45) | Yes | Yes | CD | Yes | No | NA | NA | No | Yes | No | Yes | NA | NA | NA | FAIR |
| Orqueda 2022 (16) | Yes | Yes | No | Yes | Yes | NA | NA | Yes | Yes | No | Yes | NA | NA | NA | FAIR |
| Paz-Bailey 2018 (142) | Yes | Yes | Yes | Yes | No | NA | NA | No | Yes | No | Yes | NA | NA | NA | GOOD |
| Pedraza-Bernal 2016 (88) | Yes | Yes | Yes | Yes | No | NA | NA | No | Yes | No | Yes | NA | NA | NA | GOOD |
| Pérez 2014 (17) | Yes | Yes | Yes | Yes | No | NA | NA | No | Yes | No | Yes | NA | NA | NA | GOOD |
| Pérez-Lazo 2021 (136) | Yes | Yes | No | Yes | No | NA | NA | No | Yes | No | Yes | NA | NA | NA | FAIR |
| Pinheiro Lopes 2020 (46) | Yes | Yes | CD | Yes | Yes | NA | NA | NA | Yes | No | Yes | NA | NA | NA | FAIR |
| Piña-Hincapie 2020 (89) | Yes | Yes | CD | Yes | No | NA | NA | No | Yes | No | Yes | NA | NA | NA | FAIR |
| Preciado 2015 (90) | Yes | Yes | CD | Yes | Yes | NA | NA | No | Yes | No | Yes | NA | NA | NA | FAIR |
| Pucarelli-Lebreiro 2022 (48) | Yes | Yes | CD | Yes | No | NA | NA | No | Yes | No | Yes | NA | NA | NA | FAIR |
| Puchi 2014 (70) | Yes | Yes | Yes | Yes | No | NA | NA | No | Yes | No | Yes | NA | NA | NA | GOOD |
| Quandelacy 2022 (143) | Yes | Yes | CD | Yes | No | NA | NA | No | Yes | No | Yes | NA | NA | NA | FAIR |
| Quezada 2020 (128) | Yes | No | Yes | Yes | No | NA | NA | No | Yes | No | Yes | NA | NA | NA | FAIR |
| Ramírez-Soto 2022 (137) | Yes | Yes | Yes | Yes | No | NA | NA | No | Yes | No | Yes | NA | NA | NA | GOOD |
| Riquelme 2014 (71) | Yes | Yes | Yes | Yes | No | NA | NA | No | Yes | No | Yes | NA | NA | NA | GOOD |
| Rivera Macías 2016 (108) | Yes | Yes | Yes | Yes | No | NA | NA | NA | Yes | No | Yes | NA | NA | NA | GOOD |
| Rivera-Sepulveda 2014 (144) | Yes | Yes | Yes | Yes | No | NA | NA | No | Yes | No | Yes | NA | NA | NA | GOOD |
| Robledo-Aceves 2018 (129) | Yes | Yes | Yes | Yes | No | NA | NA | Yes | Yes | No | Yes | NA | NA | NA | GOOD |
| Rodrigues de Araujo 2020 (49) | Yes | Yes | Yes | Yes | No | NA | NA | No | Yes | NA | Yes | NA | NA | NA | GOOD |
| Rodríguez-Martínez 2018 (91) | Yes | Yes | CD | Yes | No | NA | NA | No | Yes | No | Yes | NA | NA | NA | FAIR |
| Rodríguez-Santiago 2020 (145) | Yes | No | CD | Yes | No | NA | NA | No | Yes | No | Yes | NA | NA | NA | POOR |
| Rojo 2017 (18) | Yes | Yes | CD | Yes | No | NA | NA | NA | Yes | No | Yes | NA | NA | NA | FAIR |
| Romero 2013 (138) | Yes | No | CD | CD | No | NA | NA | NA | Yes | No | Yes | NA | NA | NA | POOR |
| Romero 2019 (139) | Yes | Yes | CD | Yes | No | NA | NA | NA | Yes | No | Yes | NA | NA | NA | FAIR |
| Ruiz-Ponce de León 2021 (94) | Yes | Yes | Yes | Yes | No | NA | NA | No | Yes | No | Yes | NA | NA | NA | GOOD |
| Russo 2018 (19) | Yes | Yes | Yes | Yes | No | NA | NA | NA | Yes | No | Yes | NA | NA | NA | GOOD |
| Sánchez 2021 (152) | Yes | Yes | Yes | Yes | No | NA | NA | NA | Yes | No | Yes | NA | NA | NA | GOOD |
| Saldías-Peñafiel 2016 (72) | Yes | Yes | Yes | Yes | No | NA | NA | Yes | Yes | No | Yes | NA | NA | NA | GOOD |
| Santolaya 2017 (73) | Yes | Yes | Yes | Yes | Yes | NA | NA | NA | Yes | No | Yes | NA | NA | NA | GOOD |
| Santos 2021 (50) | Yes | Yes | CD | Yes | No | NA | NA | NA | Yes | No | Yes | NA | NA | NA | FAIR |
| Serra Campelo 2022 (51) | Yes | Yes | CD | Yes | No | NA | NA | No | Yes | No | Yes | NA | NA | NA | FAIR |
| Silva 2017 (52) | Yes | Yes | CD | Yes | No | NA | NA | NA | Yes | No | Yes | NA | NA | NA | FAIR |
| Sobrinho 2021 (53) | Yes | Yes | Yes | Yes | Yes | NA | NA | NA | Yes | No | Yes | NA | NA | NA | GOOD |
| Staadegaard 2021 (119) | Yes | Yes | Yes | Yes | No | NA | NA | NA | Yes | No | Yes | NA | NA | NA | GOOD |
| Stein 2021 (54) | Yes | No | CD | Yes | No | NA | NA | NA | Yes | No | Yes | NA | NA | NA | POOR |
| Suescún-Carrero 2021 (95) | Yes | Yes | CD | Yes | No | NA | NA | NA | Yes | No | Yes | NA | NA | NA | FAIR |
| Suryadevara 2022 (105) | Yes | Yes | Yes | CD | No | NA | NA | No | Yes | No | Yes | NA | NA | NA | GOOD |
| Tinoco 2019 (140) | Yes | Yes | CD | Yes | No | NA | NA | No | Yes | No | Yes | NA | NA | NA | FAIR |
| Valencia Sapunar 2021 (74) | Yes | Yes | Yes | Yes | No | NA | NA | No | Yes | No | Yes | NA | NA | NA | GOOD |
| Varela 2022 (56) | Yes | CD | Yes | Yes | No | NA | NA | NA | Yes | No | Yes | NA | NA | NA | FAIR |
| Vasquez-Hoyos 2021a (120) | Yes | Yes | Yes | CD | No | NA | NA | NA | Yes | No | Yes | NA | NA | NA | GOOD |
| Vasquez-Hoyos 2021b (121) | Yes | Yes | Yes | CD | No | NA | NA | NA | Yes | No | Yes | NA | NA | NA | GOOD |
| Vela Avendaño 2018 (96) | Yes | Yes | CD | Yes | No | NA | NA | No | Yes | No | Yes | NA | NA | NA | FAIR |
| Vianna 2021 (57) | Yes | Yes | Yes | Yes | NA | NA | NA | NA | Yes | No | Yes | NA | NA | NA | GOOD |
| Vieira 2017 (58) | Yes | Yes | Yes | Yes | No | NA | NA | NA | Yes | No | Yes | NA | NA | NA | FAIR |
| Villamil 2019 (97) | Yes | Yes | CD | Yes | No | NA | NA | NA | Yes | No | Yes | NA | NA | NA | FAIR |
| Wollmeister 2018 (60) | Yes | Yes | CD | Yes | No | NA | NA | No | Yes | No | Yes | NA | NA | NA | FAIR |
| Yamaguto 2022 (61) | Yes | Yes | Yes | Yes | No | NA | NA | No | Yes | No | Yes | NA | NA | NA | GOOD |
| Yañez 2017 (75) | Yes | Yes | Yes | Yes | No | NA | NA | NA | Yes | No | Yes | NA | NA | NA | GOOD |
| Zarur-Torralvo 2022 (98) | Yes | Yes | Yes | Yes | NA | NA | NA | NA | Yes | No | Yes | NA | NA | NA | GOOD |
| Zuázaga 2020 (21) | Yes | Yes | Yes | Yes | No | NA | NA | NA | Yes | No | Yes | NA | NA | NA | GOOD |

* **NA**: Not applicable, **CD**: Cannot be determined

1. Was the question or research objective clearly stated in this study?
2. Was the study population clearly specified and defined?
3. Was the participation of eligible people at least 50%?
4. Were all subjects selected or recruited from the same population or from similar populations (including the same time period)? Were the inclusion and exclusion criteria for participation in the study uniformly pre-specified and applied to all participants?
5. Was a sample size justification, power description, or variance and effect estimates provided?
6. For the analysis in this study, were the exposure(s) of interest measured before the outcome(s) were measured?
7. Was the follow-up period sufficient for one to reasonably expect to see an association between exposure and outcome if it existed?
8. For exposures that may vary in quantity or level, did the study examine different levels of exposure in relation to the outcome (e.g., exposure categories or exposure measured as a continuous variable)?
9. Were exposure measures (independent variables) clearly defined, valid, reliable, and consistently implemented across all study participants?
10. Were exposures evaluated more than once over time?
11. Were outcome measures (dependent variables) clearly defined, valid, reliable, and consistently implemented across all study participants?
12. Were outcome evaluators blinded to participants' exposure status?
13. Was the loss of follow-up after study initiation 20% or less?
14. Were potential key confounding variables measured and adjusted statistically for their impact on the relationship between exposure(s) and outcome(s)?

## Supplementary Table 4. Risk of bias assessment for case series studies

| **Author, year** | **Evaluation *** | | | | | | | | | |
| --- | --- | --- | --- | --- | --- | --- | --- | --- | --- | --- |
|  | **1** | **2** | **3** | **4** | **5** | **6** | **7** | **8** | **9** | **Final** |
| Acuña 2022 (1) | Yes | Yes | CD | Yes | Yes | Yes | Yes | No | Yes | GOOD |
| Atwell 2016 (3) | Yes | Yes | CD | Yes | Yes | Yes | Yes | Yes | Yes | GOOD |
| Barbaro 2020 (4) | Yes | Yes | Yes | Yes | Yes | Yes | Yes | Yes | Yes | GOOD |
| Bettin-Martinez 2019 (78) | Yes | Yes | CD | Yes | Yes | Yes | Yes | Yes | Yes | GOOD |
| Buendía 2021a (79) | Yes | Yes | Yes | Yes | Yes | Yes | Yes | Yes | Yes | GOOD |
| Buendía 2021b (80) | Yes | Yes | CD | Yes | Yes | Yes | Yes | Yes | Yes | GOOD |
| Cesar 2018 (31) | Yes | No | CD | Yes | Yes | Yes | Yes | No | Yes | FAIR |
| Cook 2019 (8) | Yes | Yes | CD | Yes | Yes | Yes | Yes | No | Yes | GOOD |
| Ferolla 2019 (9) | Yes | Yes | CD | Yes | Yes | Yes | Yes | Yes | Yes | GOOD |
| Ferolla 2022 (10) | Yes | Yes | CD | Yes | Yes | Yes | Yes | Yes | Yes | GOOD |
| Ferrero 2022 (11) | No | No | CD | Yes | Yes | No | Yes | NA | No | POOR |
| Gatica 2014 (110) | Yes | Yes | Yes | Yes | Yes | Yes | Yes | No | Yes | GOOD |
| Hernández Marquez 2021 (141) | Yes | No | CD | Yes | Yes | Yes | CD | No | No | POOR |
| Holgado 2020 (12) | Yes | No | CD | Yes | Yes | Yes | Yes | Yes | Yes | FAIR |
| Leiva 2021 (69) | Yes | No | Yes | Yes | Yes | Yes | Yes | Yes | Yes | FAIR |
| López 2020 (14) | Yes | Yes | CD | Yes | Yes | Yes | Yes | No | Yes | GOOD |
| Lozano-Espinosa 2020 (87) | Yes | Yes | CD | Yes | Yes | Yes | Yes | Yes | Yes | GOOD |
| Machado 2016 (148) | Yes | Yes | Yes | Yes | Yes | Yes | Yes | No | Yes | GOOD |
| Machado 2018 (149) | Yes | Yes | Yes | Yes | Yes | Yes | Yes | No | Yes | GOOD |
| McCarthy 2019 (127) | Yes | Yes | CD | Yes | Yes | Yes | Yes | Yes | Yes | GOOD |
| Medeiros 2022 (41) | Yes | Yes | Yes | Yes | Yes | Yes | Yes | Yes | Yes | GOOD |
| Mendes 2021 (42) | Yes | Yes | Yes | Yes | Yes | Yes | Yes | Yes | Yes | GOOD |
| Moreira 2017 (43) | Yes | Yes | CD | Yes | Yes | Yes | Yes | Yes | Yes | GOOD |
| Pinchak 2019 (151) | Yes | Yes | CD | Yes | Yes | Yes | Yes | Yes | Yes | GOOD |
| Piva 2014 (47) | Yes | No | Yes | Yes | Yes | Yes | Yes | No | No | FAIR |
| Rodriguez-Martinez 2020 (92) | Yes | Yes | CD | Yes | Yes | Yes | Yes | Yes | Yes | GOOD |
| Rodriguez-Martinez 2022 (93) | Yes | Yes | CD | Yes | Yes | Yes | Yes | Yes | Yes | GOOD |
| Tabor 2021 (55) | Yes | No | CD | CD | Yes | Yes | NA | Yes | Yes | FAIR |
| Viegas 2016 (20) | Yes | Yes | Yes | CD | Yes | No | NA | Yes | Yes | FAIR |
| Watanabe 2022 (59) | Yes | Yes | Yes | Yes | Yes | Yes | Yes | Yes | Yes | GOOD |
| Zepeda 2018 (76) | Yes | Yes | Yes | Yes | Yes | Yes | Yes | Yes | Yes | GOOD |

* **NA**: Not applicable, **CD**: Cannot be determined

1. Was the study question or objective clearly specified?
2. Was the study population clearly and fully described, including case definition?
3. Were cases consecutive?
4. Were subjects comparable?
5. Was exposure clearly described?
6. Were measures of results clearly defined, valid, reliable and consistently implemented for all study participants?
7. Was the length of follow-up appropriate?
8. Were statistical methods properly described?
9. Were results properly described?

## Supplementary Table 5. Risk of bias assessment for case-control studies

| **Author, year** | **Evaluation *** | | | | | | | | | | | | |
| --- | --- | --- | --- | --- | --- | --- | --- | --- | --- | --- | --- | --- | --- |
|  | **1** | **2** | **3** | **4** | **5** | **6** | **7** | **8** | **9** | **10** | **11** | **12** | **Final** |
| López de Orellana 2017 (107) | Yes | Yes | Yes | Yes | Yes | Yes | CD | CD | Yes | Yes | CD | No | FAIR |

* **NA**: Not applicable, **CD**: Cannot be determined

1. Was the research question or objective in this paper clearly stated and appropriate?
2. Was the study population clearly specified and defined?
3. Did the authors include a sample size justification?
4. Were controls selected or recruited from the same or similar population that gave rise to the cases (including the same timeframe)?
5. Were the definitions, inclusion and exclusion criteria, algorithms or processes used to identify or select cases and controls valid, reliable, and implemented consistently across all study participants?
6. Were the cases clearly defined and differentiated from controls?
7. If less than 100 percent of eligible cases and/or controls were selected for the study, were the cases and/or controls randomly selected from those eligible?
8. Was there use of concurrent controls?
9. Were the investigators able to confirm that the exposure/risk occurred prior to the development of the condition or event that defined a participant as a case?
10. Were the measures of exposure/risk clearly defined, valid, reliable, and implemented consistently (including the same time period) across all study participants?
11. Were the assessors of exposure/risk blinded to the case or control status of participants?
12. Were key potential confounding variables measured and adjusted statistically in the analyses? If matching was used, did the investigators account for matching during study analysis?

# Supplementary Table 6. AGREE-ll assessment for guidelines

| **Author, year** | **Domain 1: scope and purpose** | **Domain 2: stakeholder involvement** | **Domain 3: rigor of development** | **Domain 4: clarity of presentation** | **Domain 5: applicability** | **Domain 6: editorial independence** | **Overall** |
| --- | --- | --- | --- | --- | --- | --- | --- |
| **Fuentes 2016**  (180) | **Score: 17%** | **Score: 6%** | **Score: 2%** | **Score: 36%** | **Score: 0%** | **Score: 0%** | **Score^a^: A: 1; B: N** |
|  | The recommendations are based on general bronchiolitis therapy. Objective is not clearly described and it does not include a specific health question (PICO). The target population and its specific risk is ambiguously discussed. | Pediatricians are defined as target users. No description of how guidelines may be used is provided. No information about the role of authors is presented. No focus groups were conducted and no information about input from patients/target groups was provided. | Document is purely based on literature review. No search strategy, selection criteria, information of the strengths and limitation of evidence are provided. No information about external review is provided. No information is provided for future updates. | Identification of the relevant populations is given but is not specific, e.g. referring to children with acute bronchiolitis but does not provide age-specific recommendations. It includes a range of therapy alternatives that are linked to clinical evidence. Key recommendations are easily identifiable. | No implementation barriers and facilitators are described and no implementation tools are provided. The guideline does not include cost-benefit data. Monitoring and audit criteria are not provided. | No funding source and /or conflict of interest have been declared. No competing interests have been declared. | This is a narrative review of treatment for bronchitis of any origin. It includes little information on the use of Palivizumab. |
| **Ribeiro 2016** (179) | **Score: 25%** | **Score: 36%** | **Score: 1%** | **Score: 67%** | **Score: 0%** | **Score: 0%** | **Score^a^: A: 3; B: N** |
|  | Objective includes a health intent (prevention) and ambiguously the target population. It is not easy to find (it is only included in the abstract). A specific health question (PICO) is not included. | The target users of the guideline are clearly defined. The guideline development group does not include all relevant professional groups. No information about input from patients/target group is provided. Some information about the procedure of prescribing/receiving Palivizumab is provided. | Document is a narrative review of existing guidelines. No search strategy, selection criteria, information of the strengths and limitation of evidence are provided. No information about external review is provided. No information is provided for future updates. | Provided recommendations are specific and unambiguous. Age specific recommendation included. No clinical evidence is presented although resource links to evidence are described. Recommendations are listed in bullet points for high-risk populations. | No implementation barriers and facilitators are described, and no implementation tools are provided. The guideline does not include cost-benefit data. Monitoring and audit criteria are not provided. | No funding source and /or conflict of interest have been declared. No competing interests have been declared. | It provides an overview of recommendations and includes the source for studies that provide evidence. Due to the type of study design (narrative review) the inclusion of all the available evidence is not assured. |
| **Martínez 2019**  (178) | **Score: 25%** | **Score: 3%** | **Score: 5%** | **Score: 36%** | **Score: 6%** | **Score: 100%** | **Score^a^: A: 2; B: N** |
|  | No specific health intent is provided. The document does not include a specific health question (PICO). The guideline discusses target population (premature infants) and specific population with risk factors. | Revision does not include a clear description of the target audience and description of how the guide can be used. No information about the role of authors is presented. No focus groups were conducted and no information about input from patients/target groups was provided. | Document is a narrative review of existing guidelines. No search strategy is used, or selection criteria is mentioned. Information of the strengths and limitations of evidence is provided. No information about external review or future update guidelines are provided. | Detailed recommendations for different risk and age groups are provided. No alternative preventive options are presented. Clinical evidence is presented. Recommendations are listed in bullet points. | Barriers are mentioned regarding cost-effectiveness and the lack of existing local studies. Specific country data is provided, i.e. clinical evidence. No implementation section or additional material are provided. The guideline does not include cost-benefit data. | Funding source and /or conflict of interest have been declared. Competing interests have been declared. | It provides an overview of recommendations and includes the source for studies that provide evidence. Due to the type of study design (narrative review) the inclusion of all the available evidence is not assured. |
| **Fernández Jonusas 2016**  (177) | **Score: 17%** | **Score: 31%** | **Score: 18%** | **Score: 86%** | **Score: 6%** | **Score: 0%** | **Score^a^: A: 4; B: N** |
|  | Objective is not clearly described. Target population is ambiguously mentioned. The document does not include a specific PICO question. | The guideline development group includes individuals from all relevant professional groups. No focus groups were conducted and no information about input from patients/target groups was provided. The target users of the guideline are not clearly defined. | No search strategy, inclusion or exclusion criteria, information of the strengths and limitation of evidence are provided. No information about external review and future update is provided. Clinical evidence supports recommendations. No side effects or risk factors discussed. No information about external review is provided. | Detailed recommendations for different risk and age groups are provided. Clinical evidence is presented to highlight its benefits. Recommendations are listed in bullet points for high-risk populations. | No implementation section or additional tools are provided. No cost implications are provided. No economic evaluation has been conducted. The guideline does not include cost-benefit data. Monitoring and audit criteria are provided. No criteria for assessing the impact of the implementation are provided. | No funding source and /or conflict of interest have been declared. No competing interests have been declared. | Good review of available evidence, providing specific information and recommendations. Lacks specifications for the applicability of the intervention. |

^a^A: Rate the overall quality of this guideline: scoring: 1 (least quality) to 7 (highest quality); B: I would recommend this guideline for use, Y: Yes; M: Yes, with modification; N: No

# Burden of RSV disease: meta-analysis

## Supplementary Figure 1. RSV/suspected cases 0-2 years


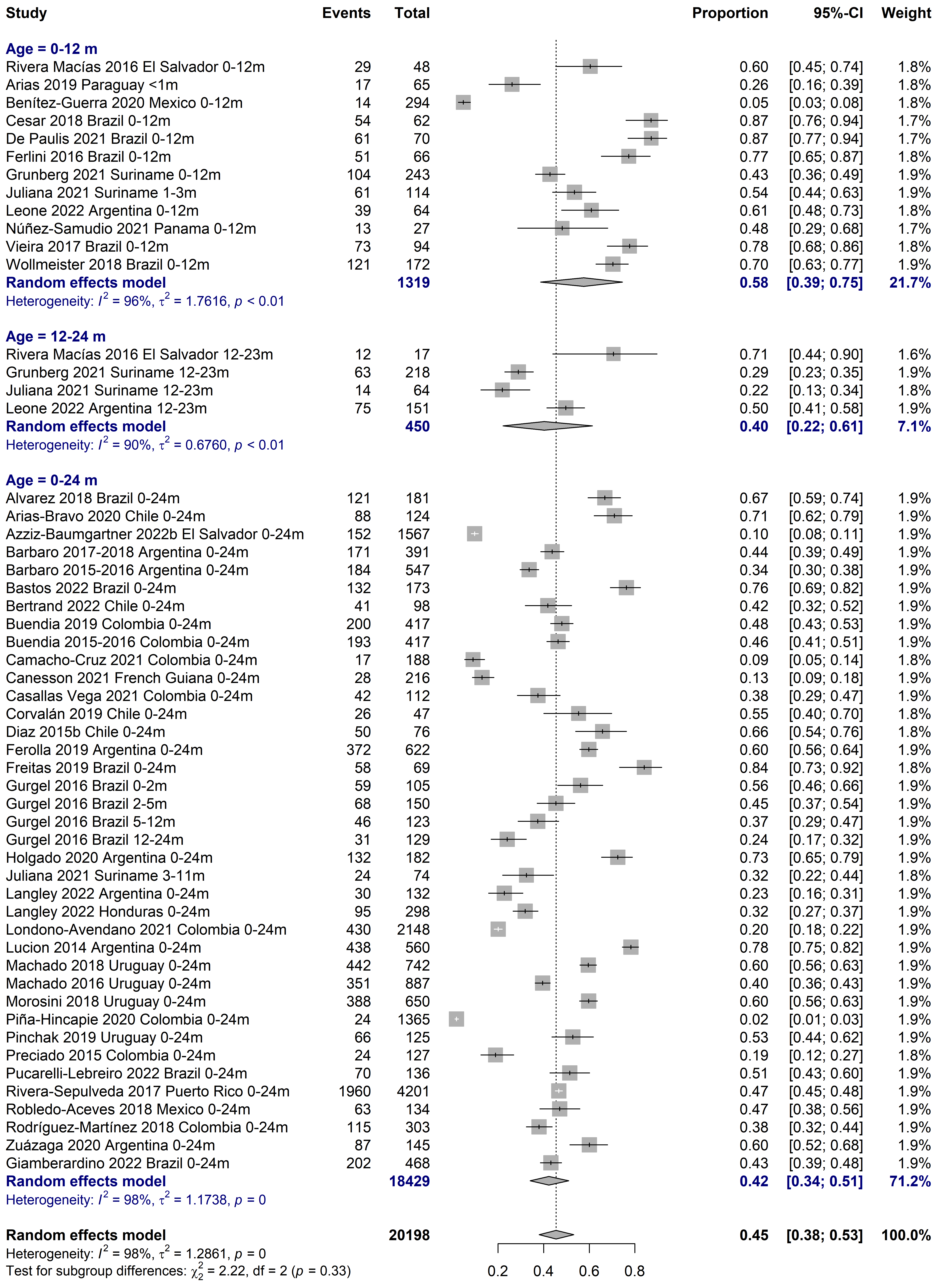


## Supplementary Figure 2. RSV/suspected cases 0-2 years by five year calendar period (2012-2016 and 2017-2022)

**
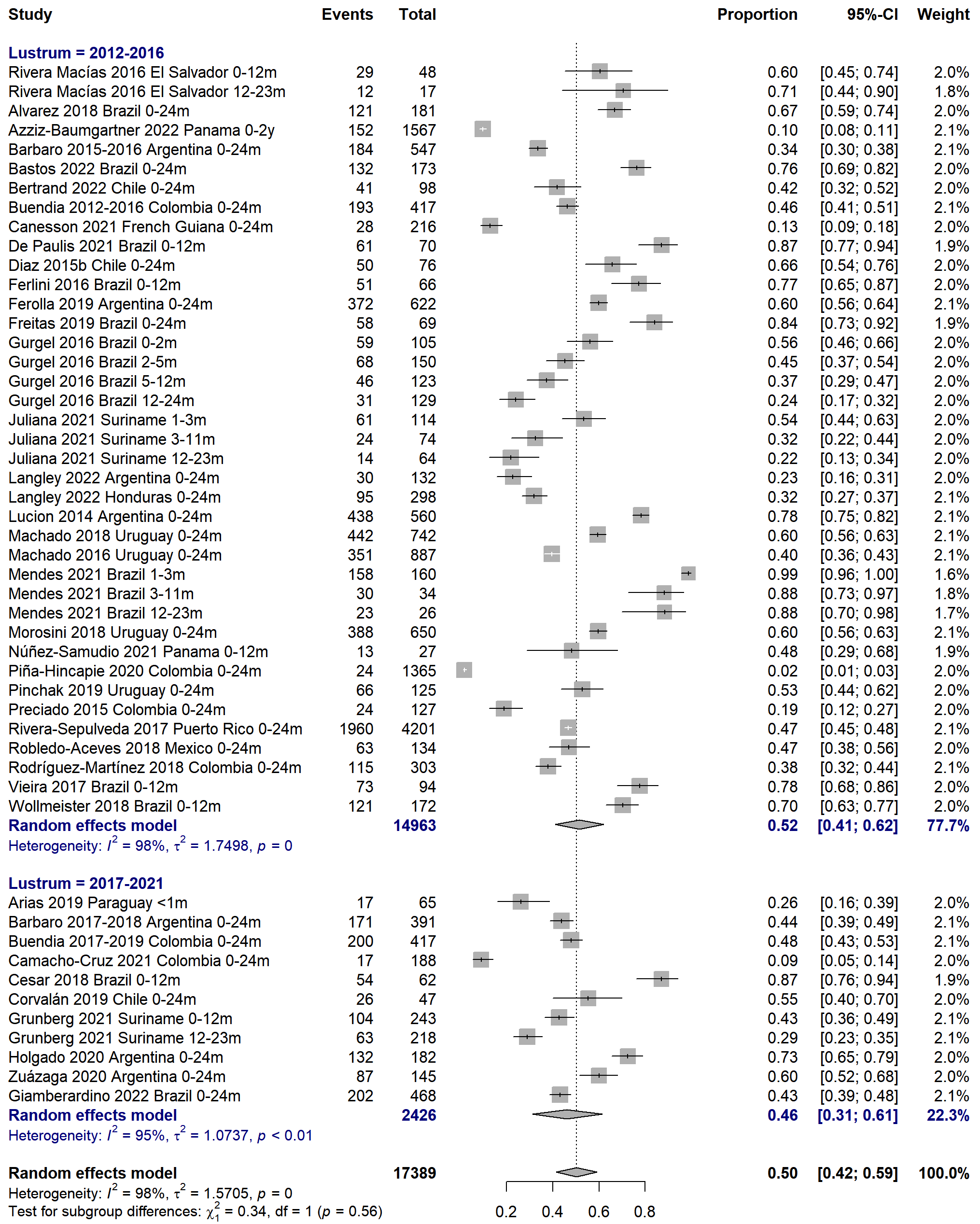
**

## Supplementary Figure 3. RSV/suspected cases 0-2 years by risk group (high and normal risk)

**
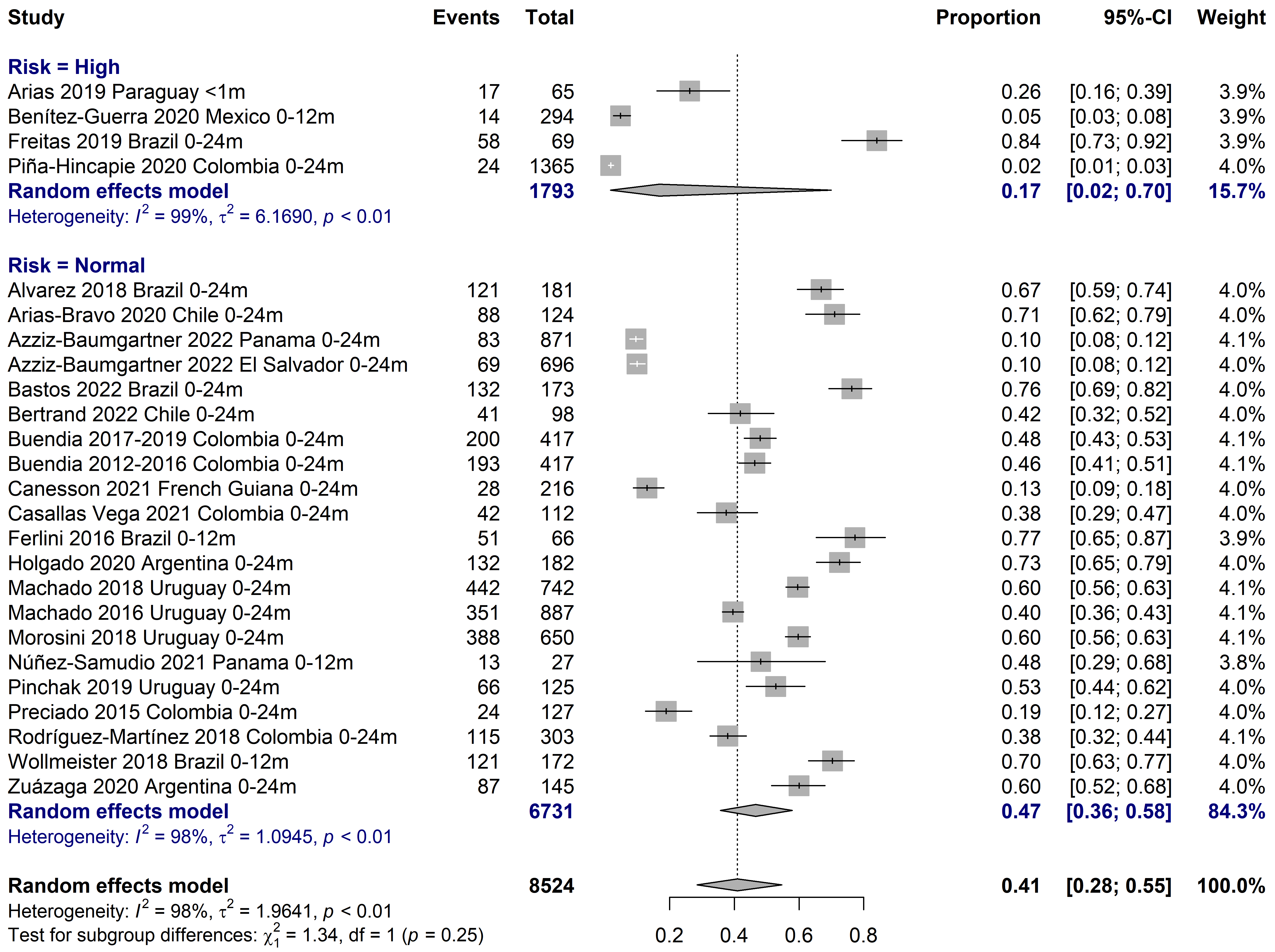
**

## Supplementary Figure 4. RSV/suspected cases 0-2 years by low ROB

**
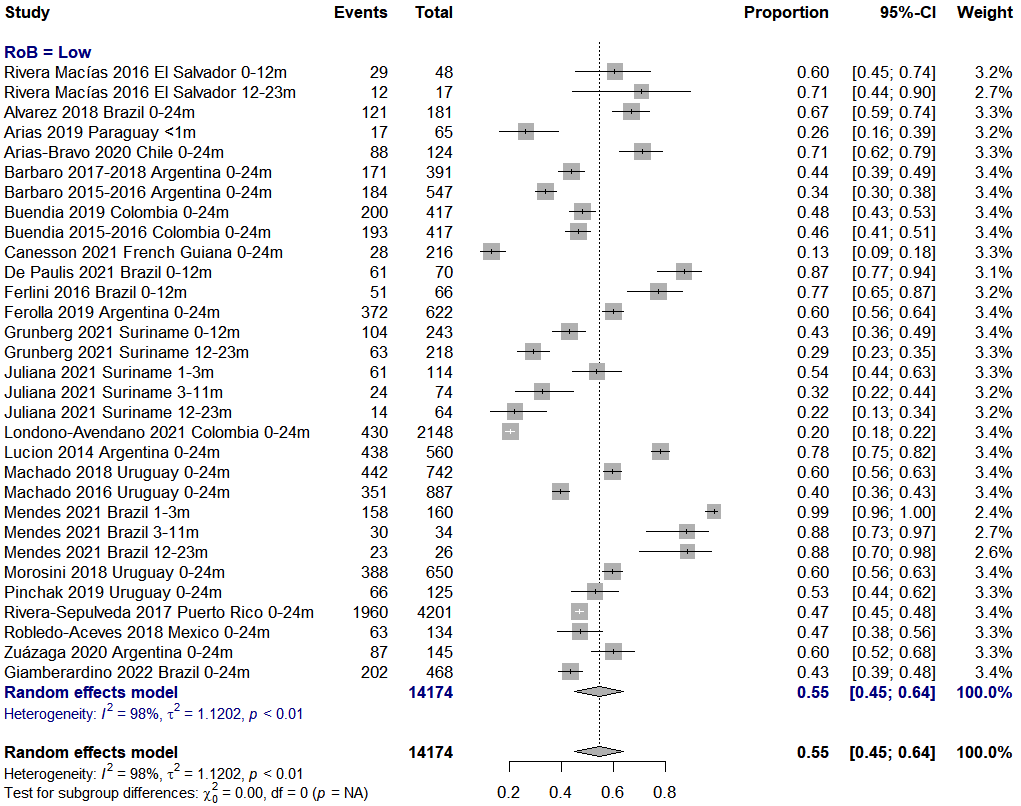
**

## Supplementary Figure 5. RSV/suspected cases 5-14 years by five year calendar period (2012-2016 and 2017-2022)


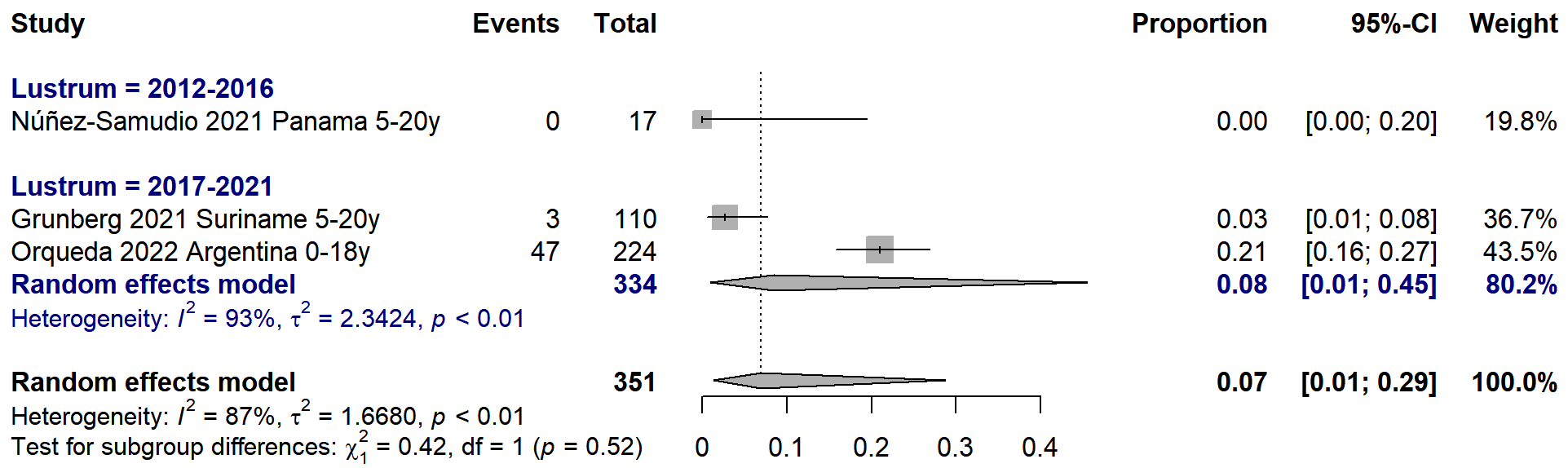


## Supplementary Figure 6. RSV/suspected cases ≥65 years by five year calendar period (2012-2016 and 2017-2022)


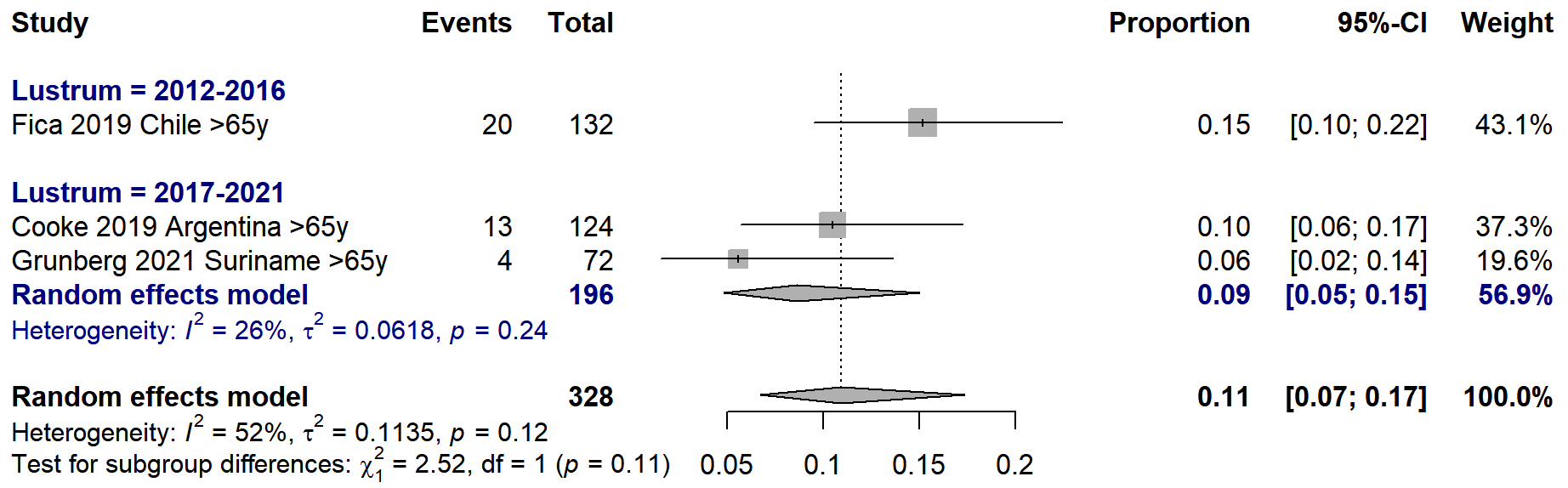


## Supplementary Figure 7. RSV/suspected cases ≥65 years by risk group (high and normal risk)

**
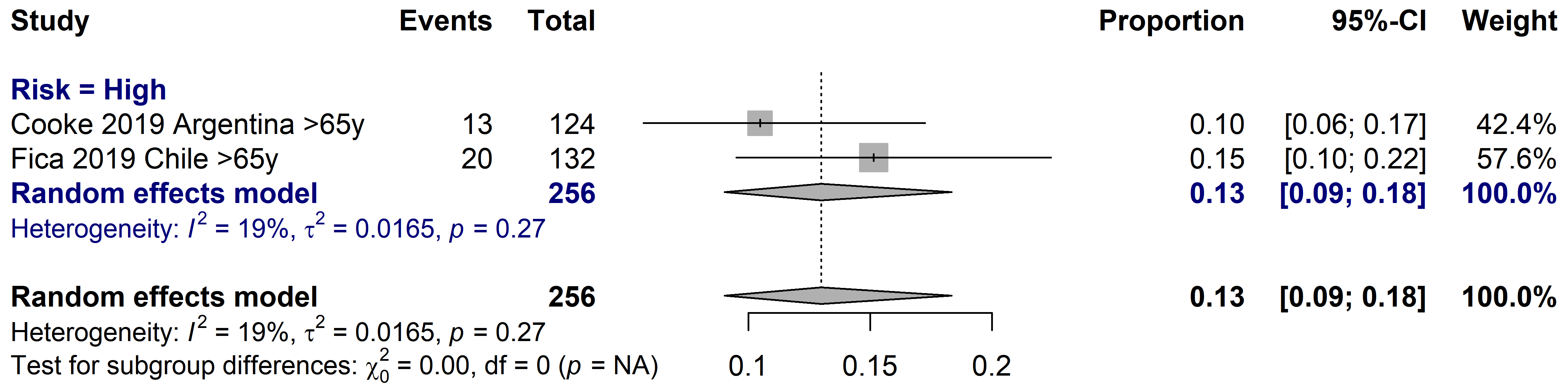
**

## Supplementary Figure 8. RSV LRTI/100 symptomatic p-y (mean) 0-2 years

**
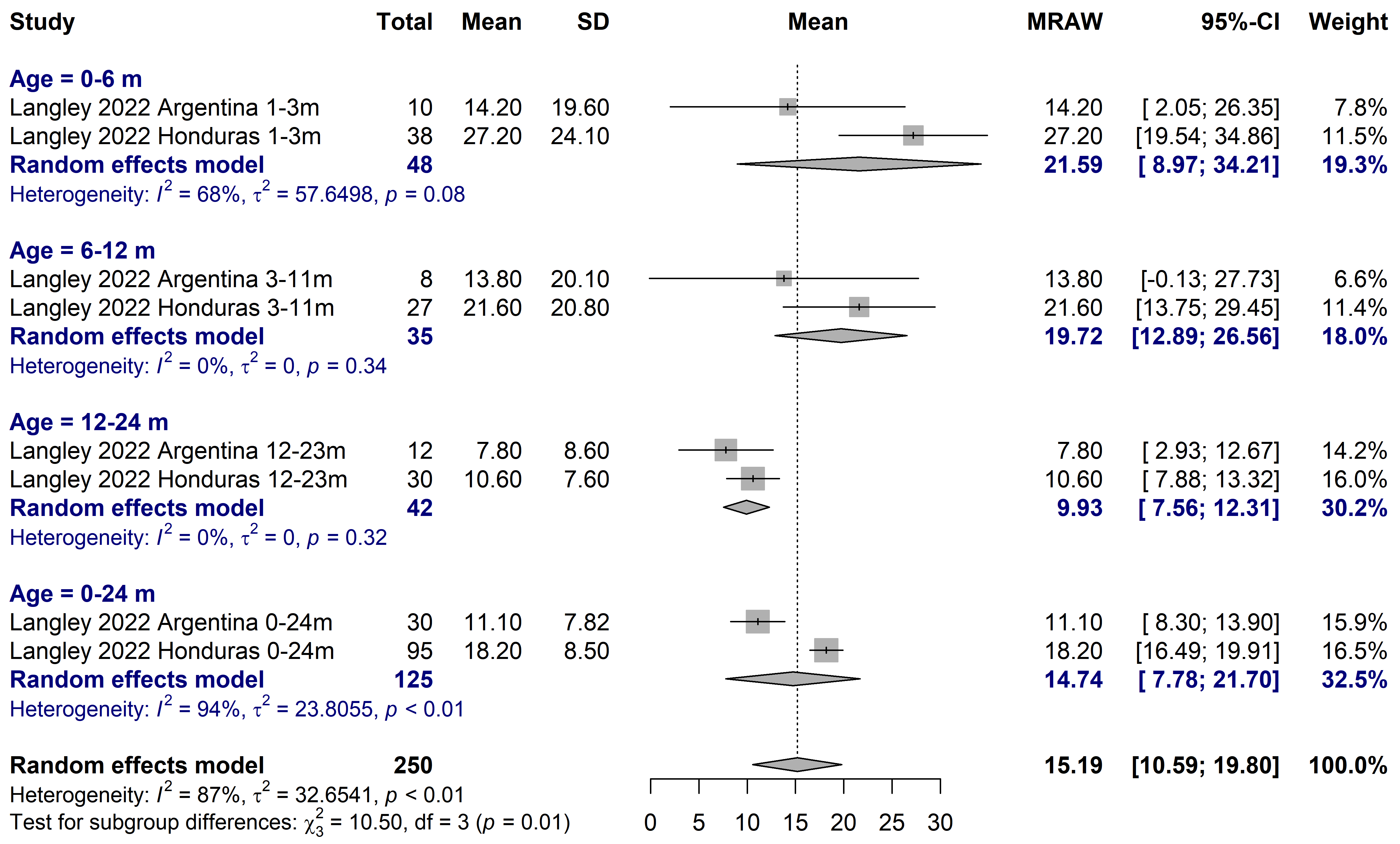
**

## Supplementary Figure 9. LRTI/RSV cases 0-2 years


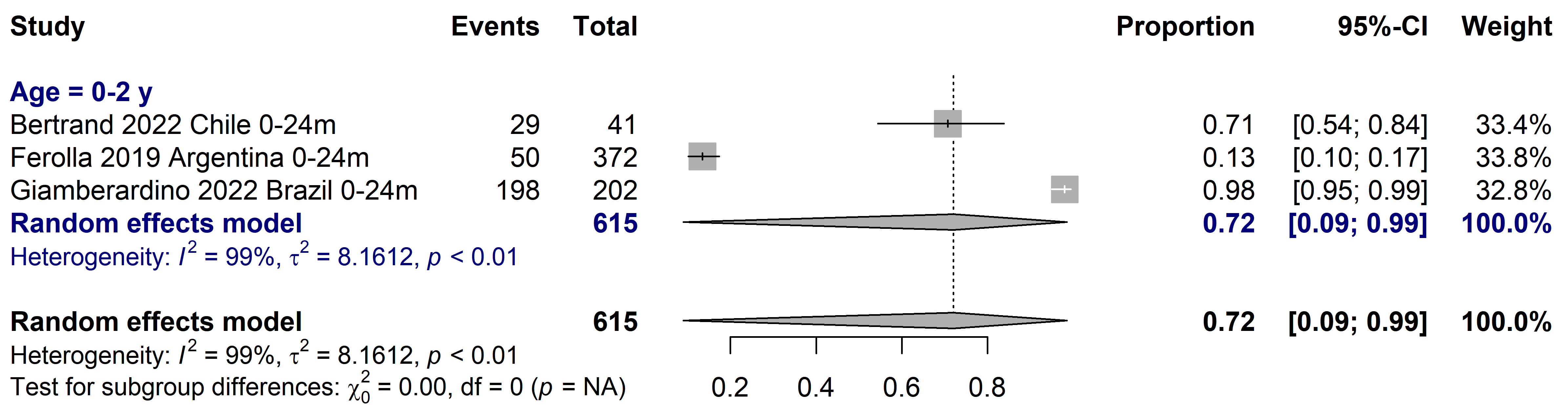


## Supplementary Figure 10. LRTI/RSV cases 0-2 years by five year calendar period (2012-2016 and 2017-2022)


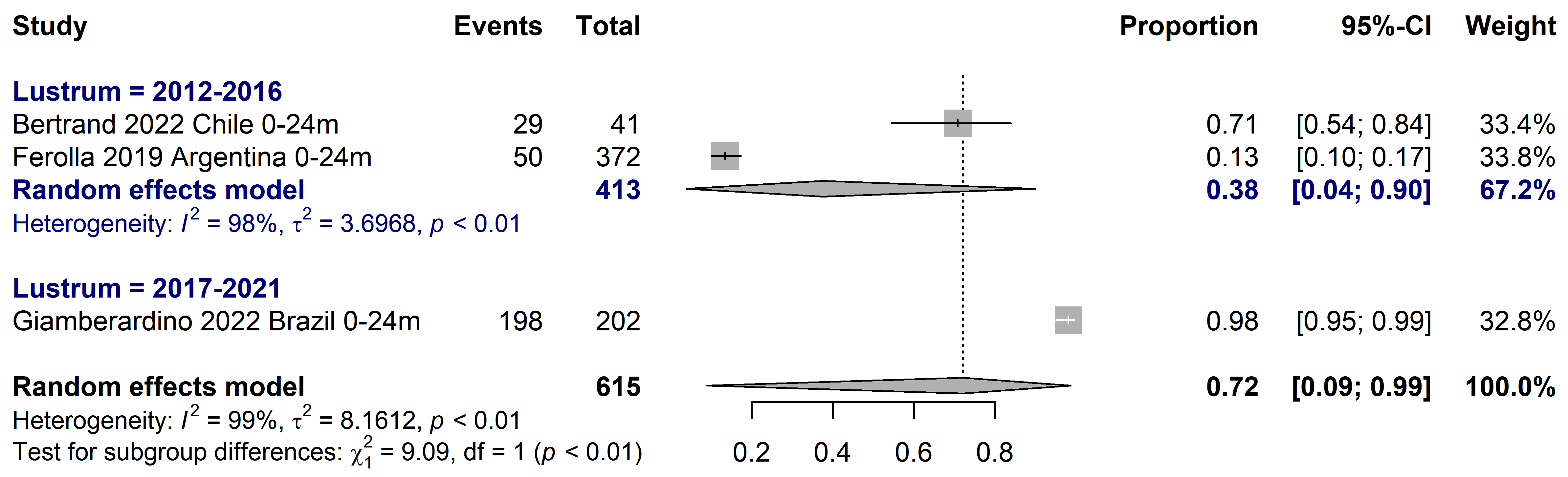


## Supplementary Figure 11. LRTI/RSV cases 0-2 years by low ROB


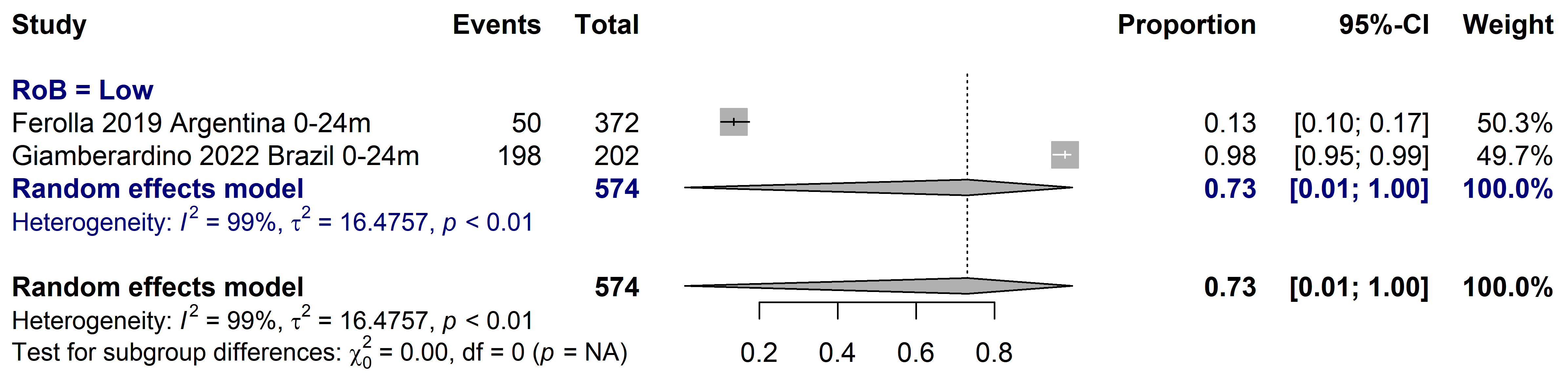


## Supplementary Figure 12. LRTI/RSV cases 0-5 years by five year calendar period (2012-2016 and 2017-2022)


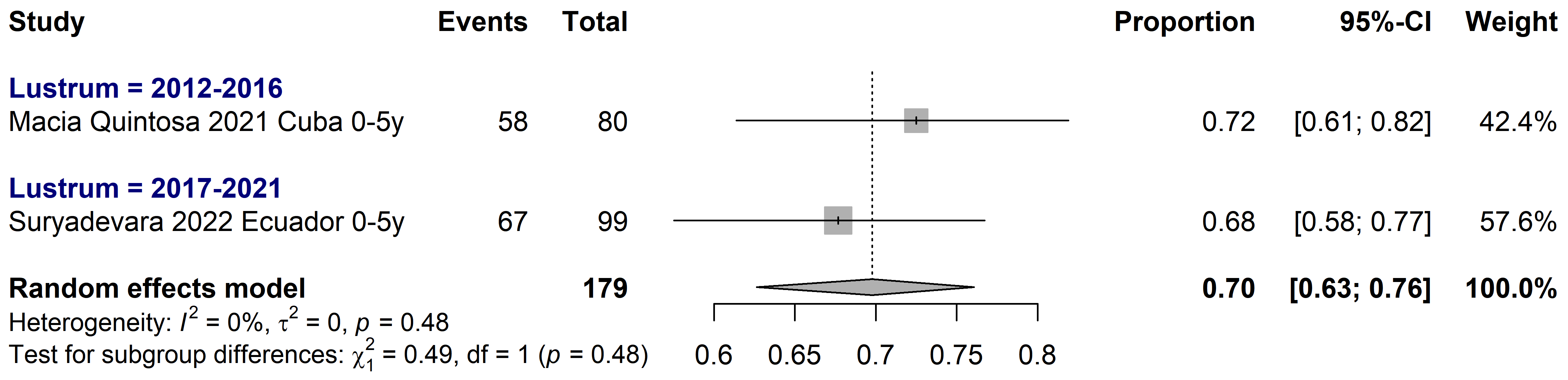


## Supplementary Figure 13. LRTI/RSV cases 0-18 years by five year calendar period (2012-2016 and 2017-2022)


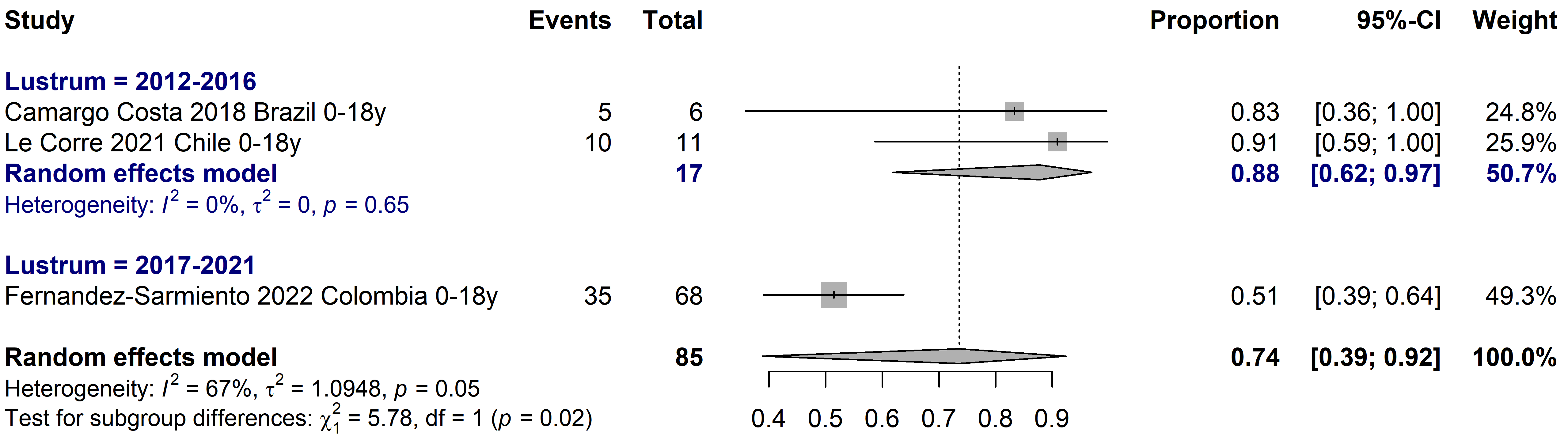


## Supplementary Figure 14. Lethality of RSV LRTI 0-2 years by five year calendar period (2012-2016 and 2017-2022)

**
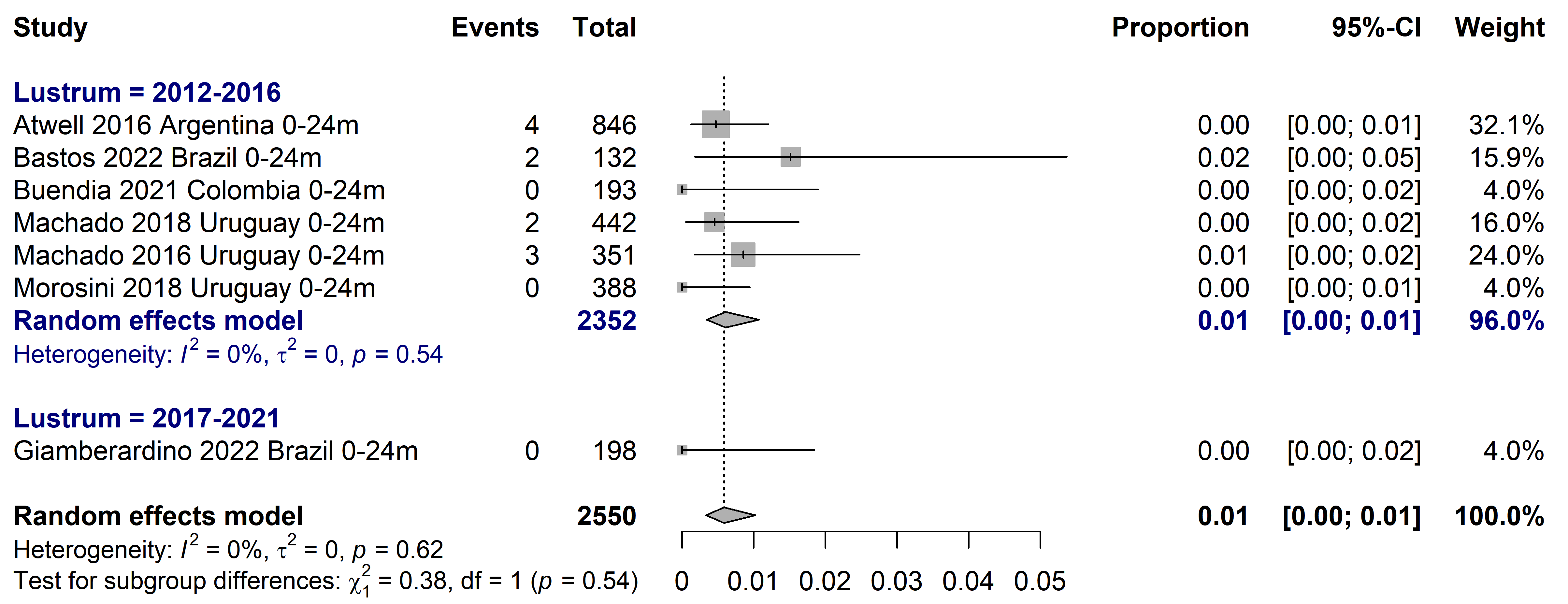
**

## Supplementary Figure 15. Lethality of RSV LRTI 0-2 years by low ROB


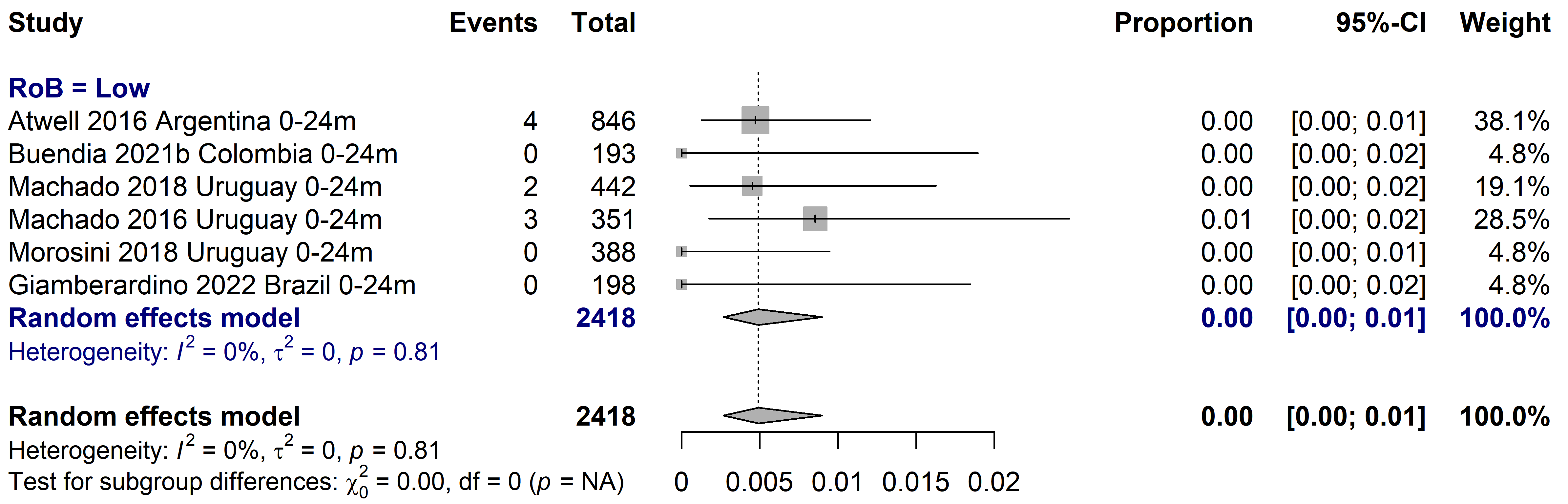


## Supplementary Figure 16. Lethality of RSV LRTI 0-5 years


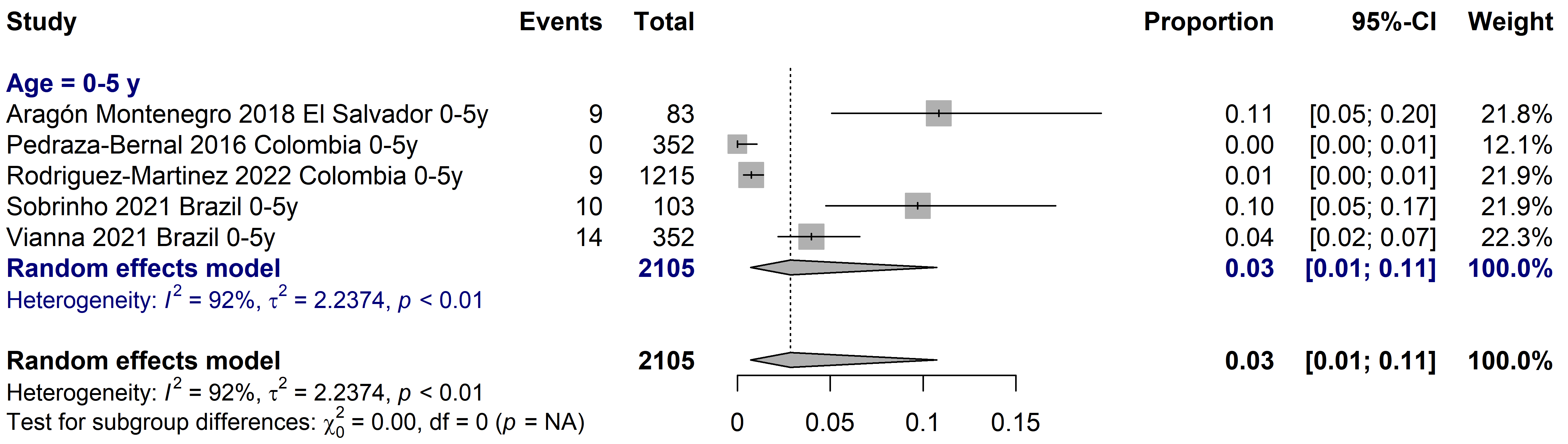


## Supplementary Figure 17. Lethality of RSV LRTI 0-5 years by five year calendar period (2012-2016 and 2017-2022)


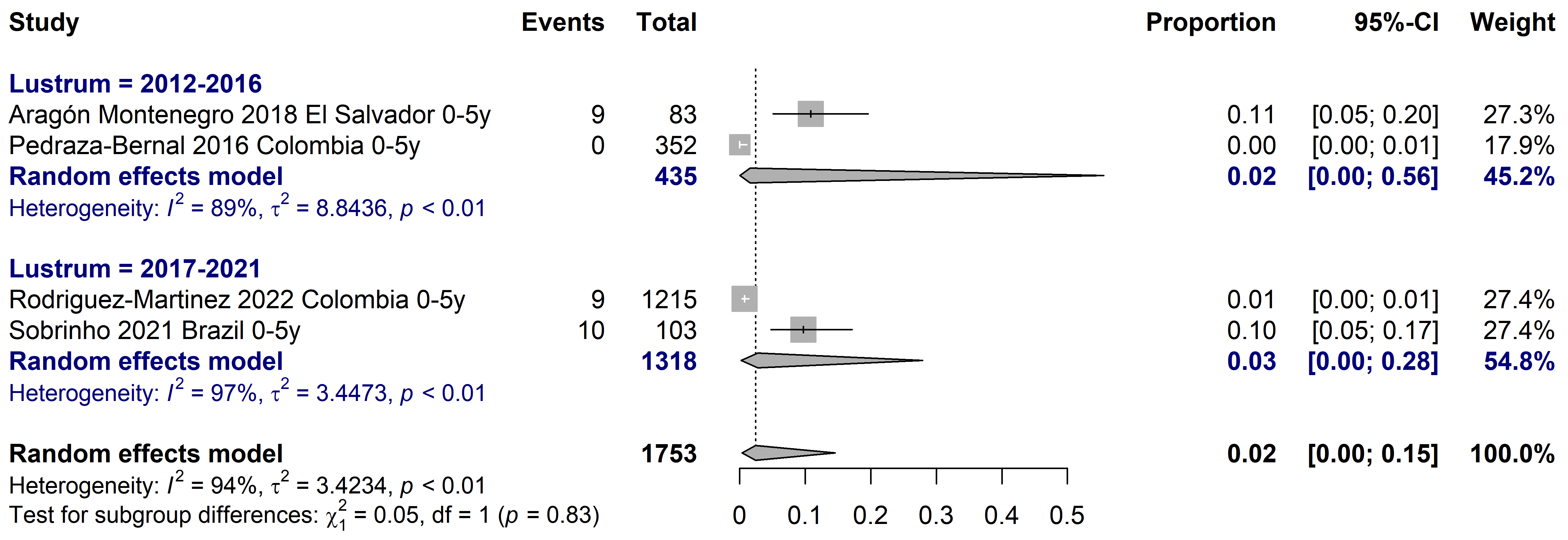


## Supplementary Figure 18. Bronchiolitis/RSV cases 0-2 years


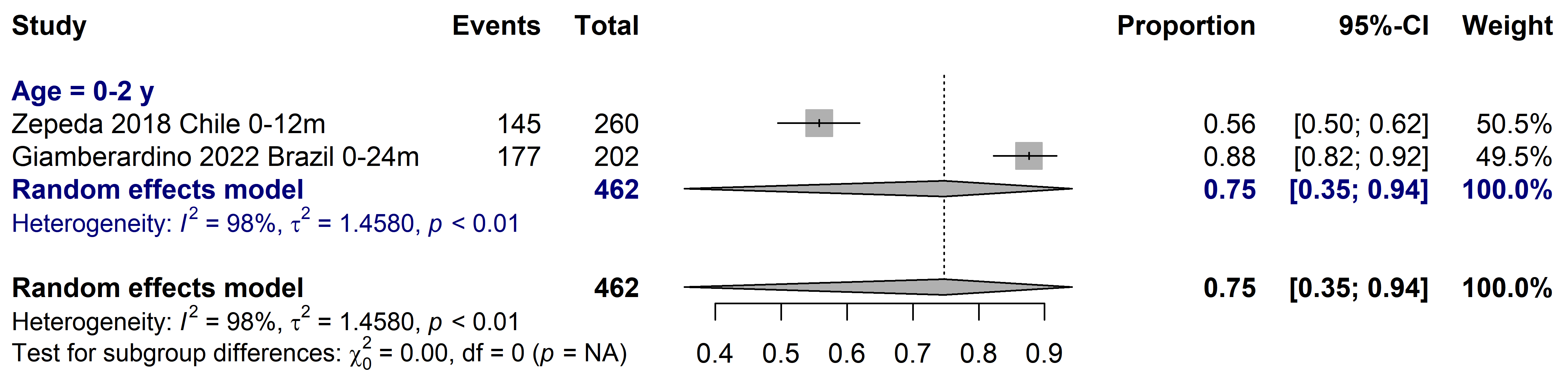


## Supplementary Figure 19. Bronchiolitis/RSV cases 0-5 years by five year calendar period (2012-2016 and 2017-2022)

**
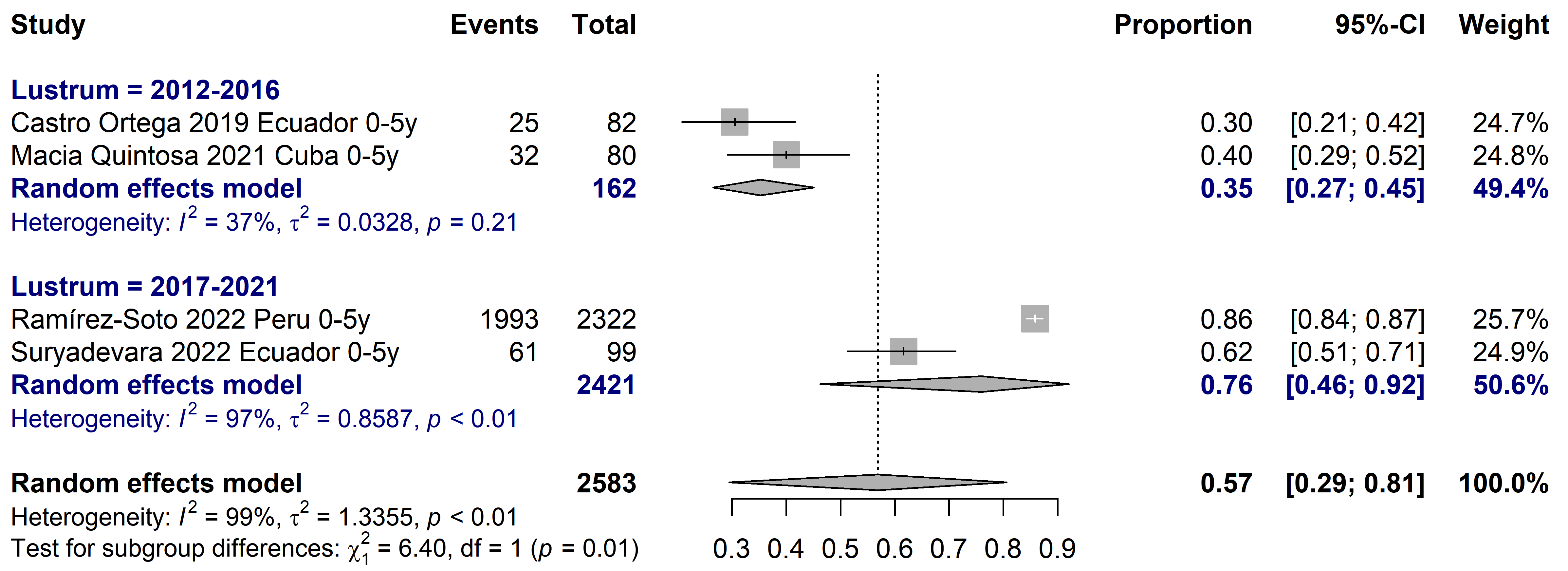
**

## Supplementary Figure 20. Bronchiolitis/RSV cases 0-5 years by risk group (high and normal risk)

**
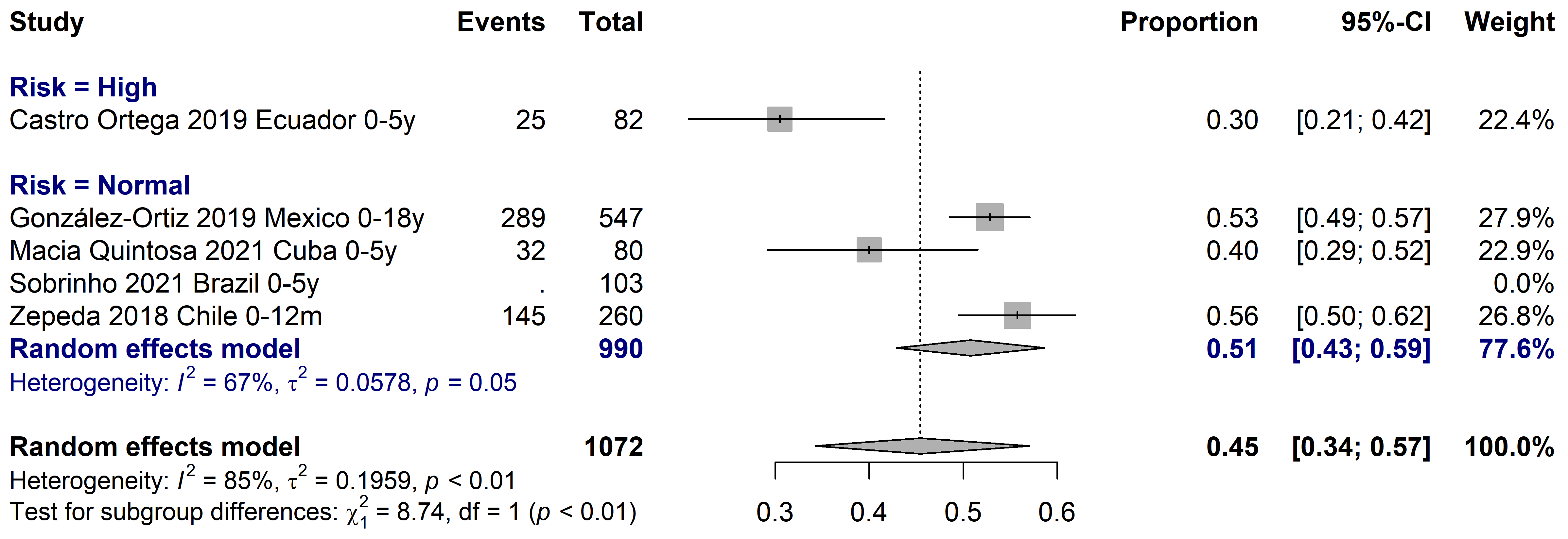
**

## Supplementary Figure 21. Bronchiolitis/RSV cases 0-5 years by low ROB

**
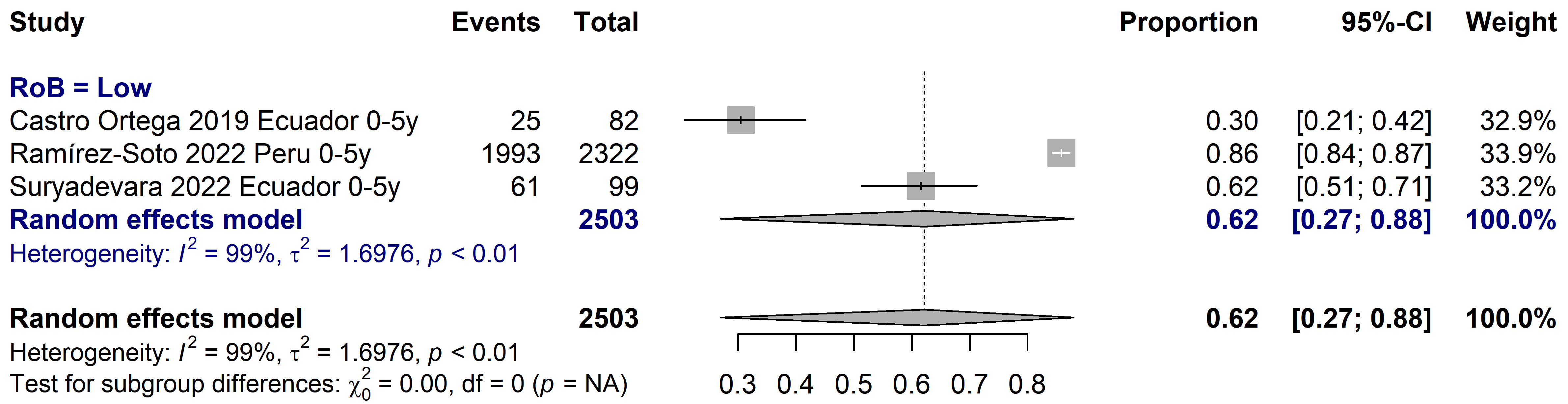
**

## Supplementary Figure 22. Bronchiolitis/LRTI cases 0-2 years

**
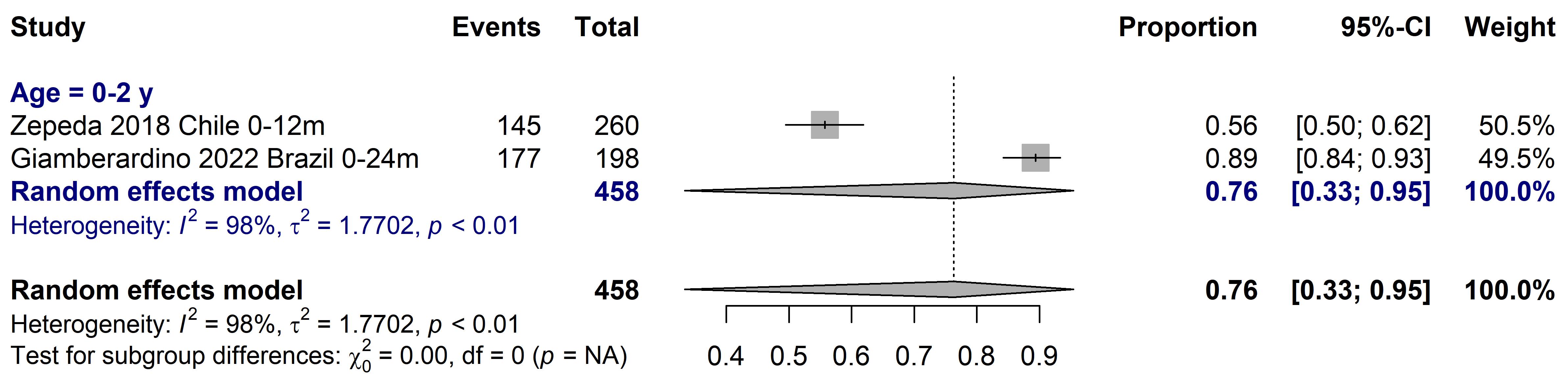
**

## Supplementary Figure 23. Bronchiolitis/LRTI cases 0-5 years

**
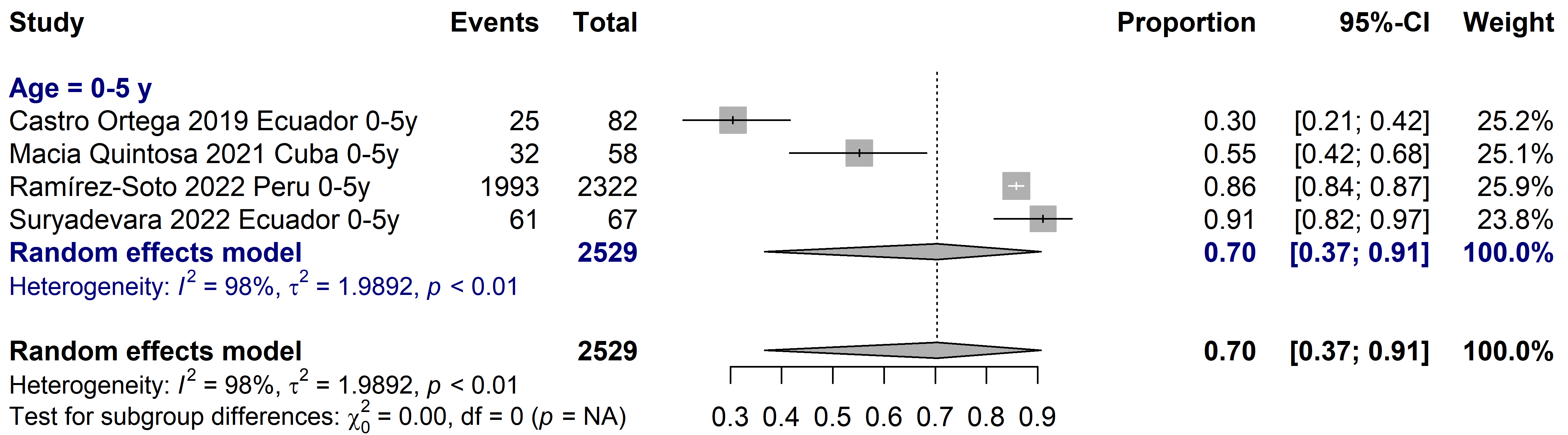
**

## Supplementary Figure 24. Bronchiolitis/LRTI cases 0-5 years by five year calendar period (2012-2016 and 2017-2022)

**
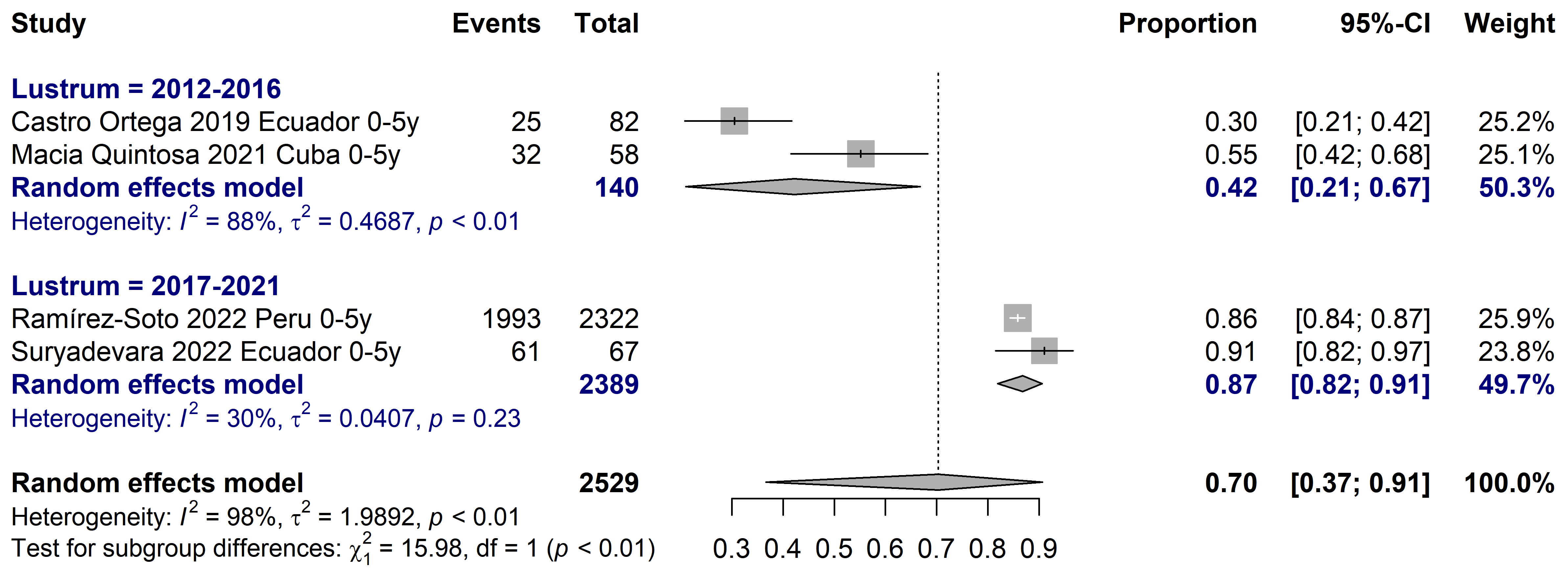
**

## Supplementary Figure 25. Bronchiolitis/LRTI cases 0-5 years by risk group (high and normal risk)

**
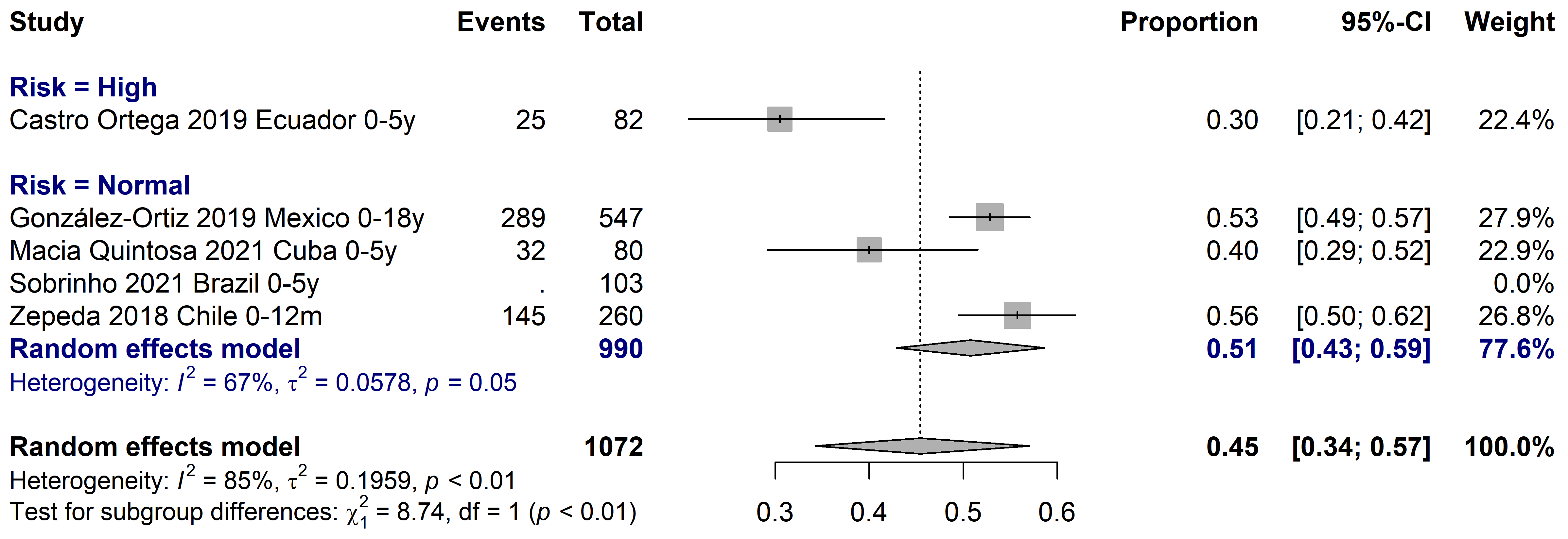
**

## Supplementary Figure 26. Bronchiolitis/LRTI cases 0-5 years by low ROB

**
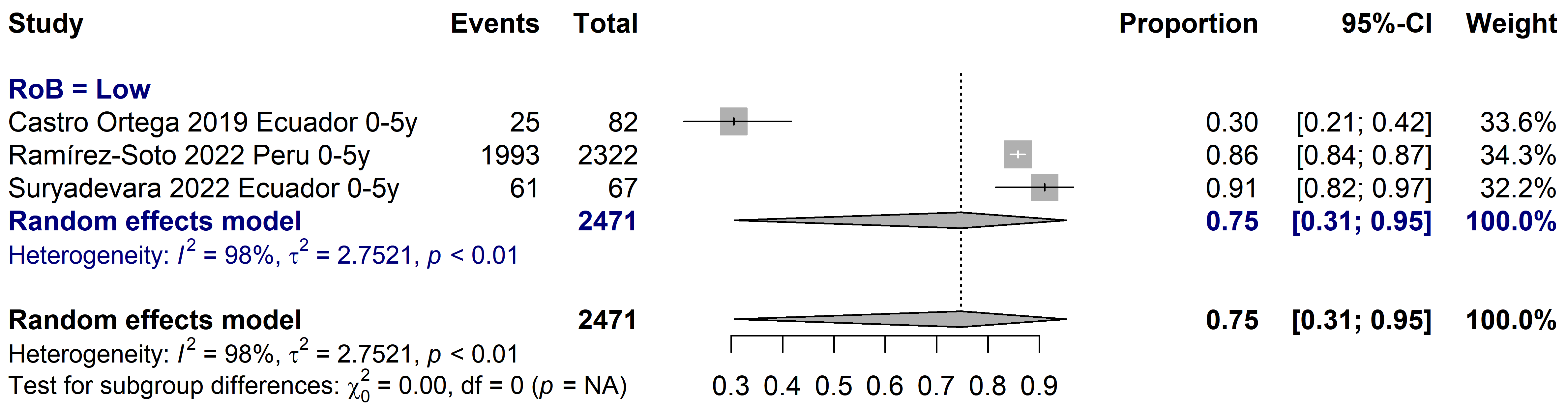
**

## Supplementary Figure 27. ICU admissions /RSV LRTI 0-2 years


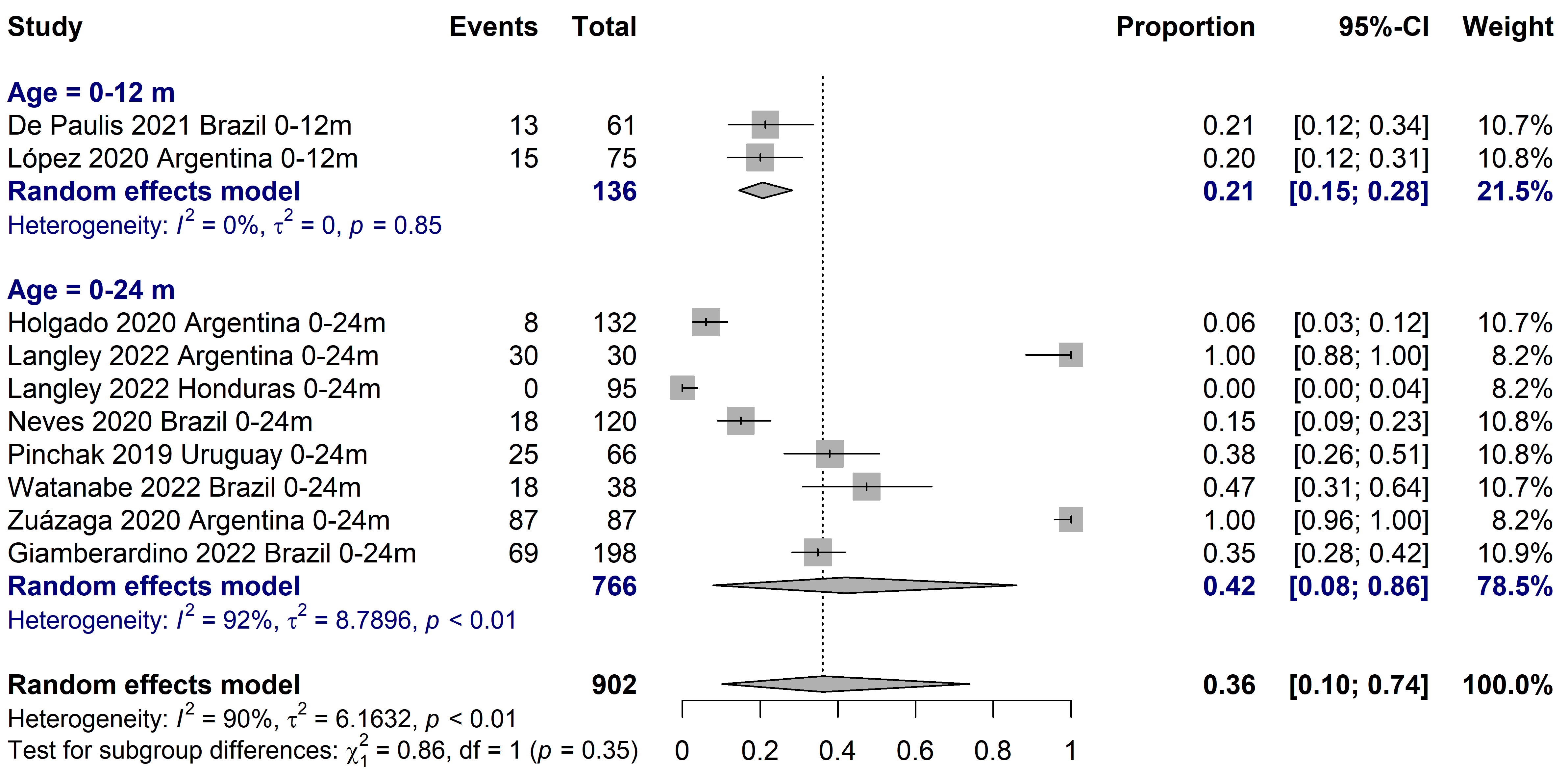


## Supplementary Figure 28. ICU admissions /RSV LRTI 0-2 years by five year calendar period (2012-2016 and 2017-2022)

**
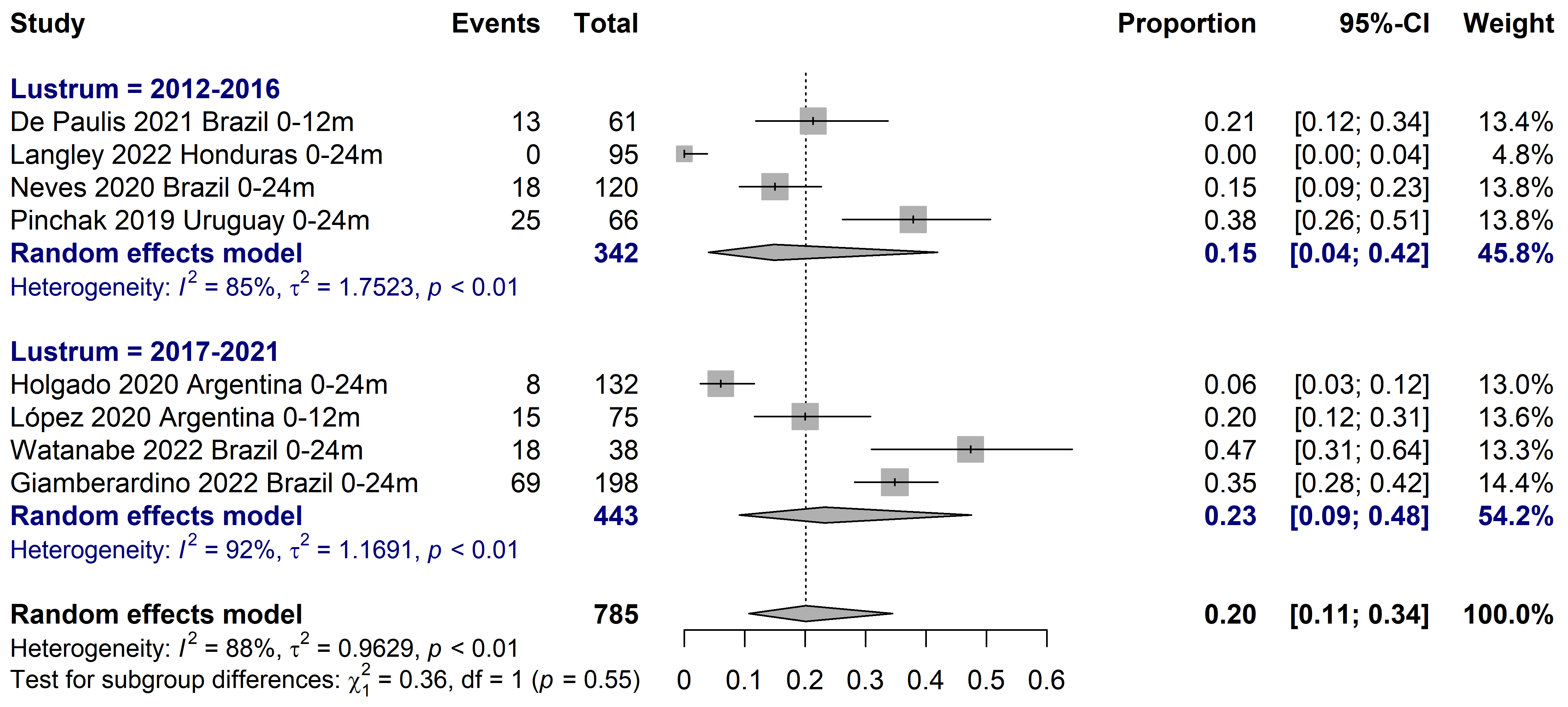
**

## Supplementary Figure 29. Length of stay in ICU (days) 0-2 years

**
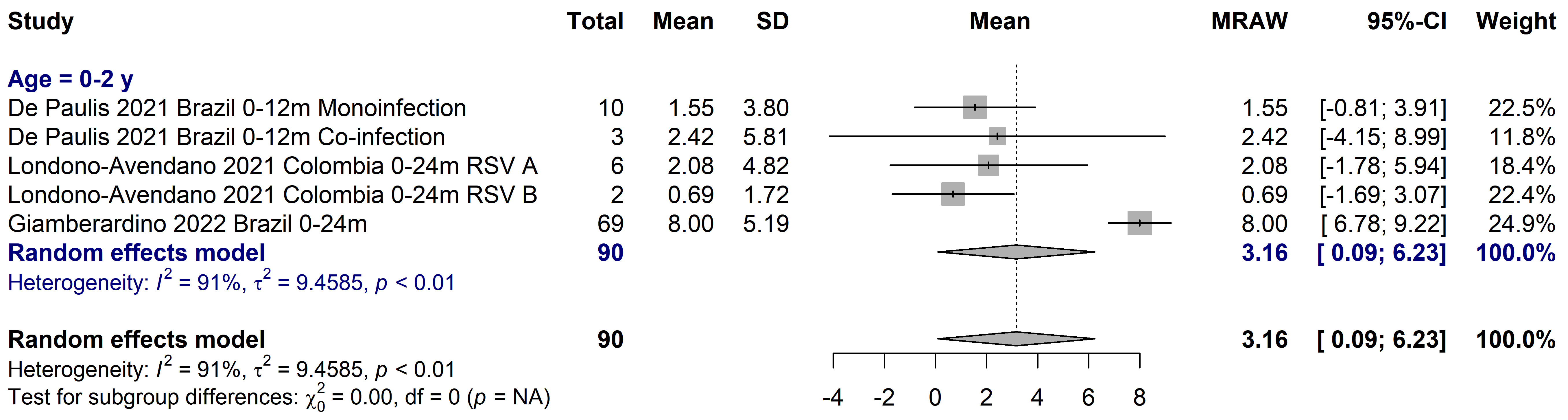
**

## Supplementary Figure 30. Length of stay in ICU (days) 0-2 years by five year calendar period (2012-2016 and 2017-2022)

**
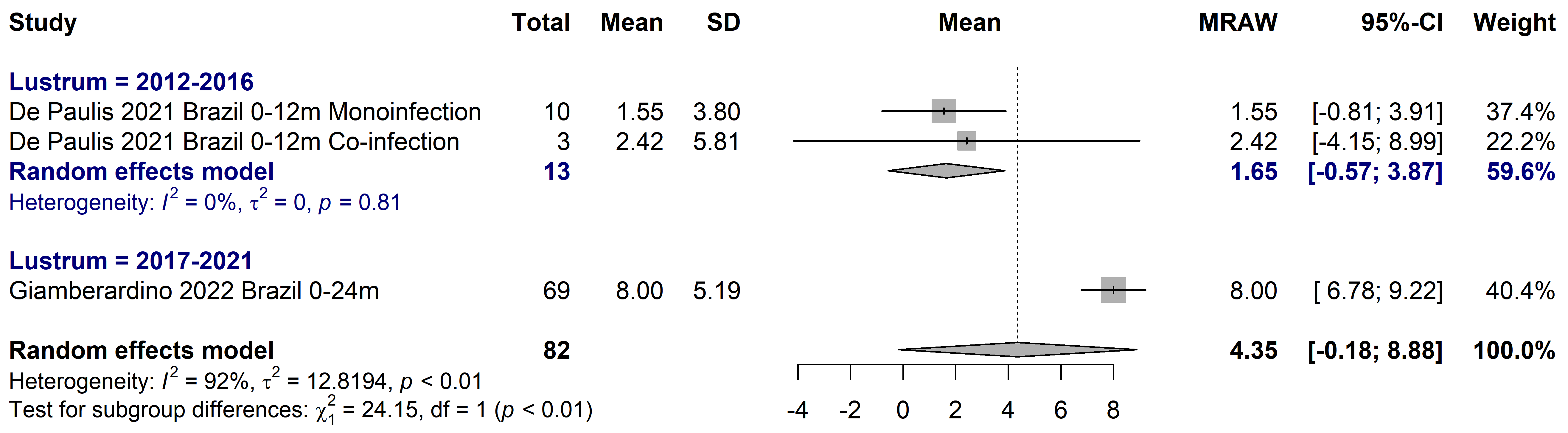
**

## Supplementary Figure 31. Length of stay in general ward (days) 0-2 years

**
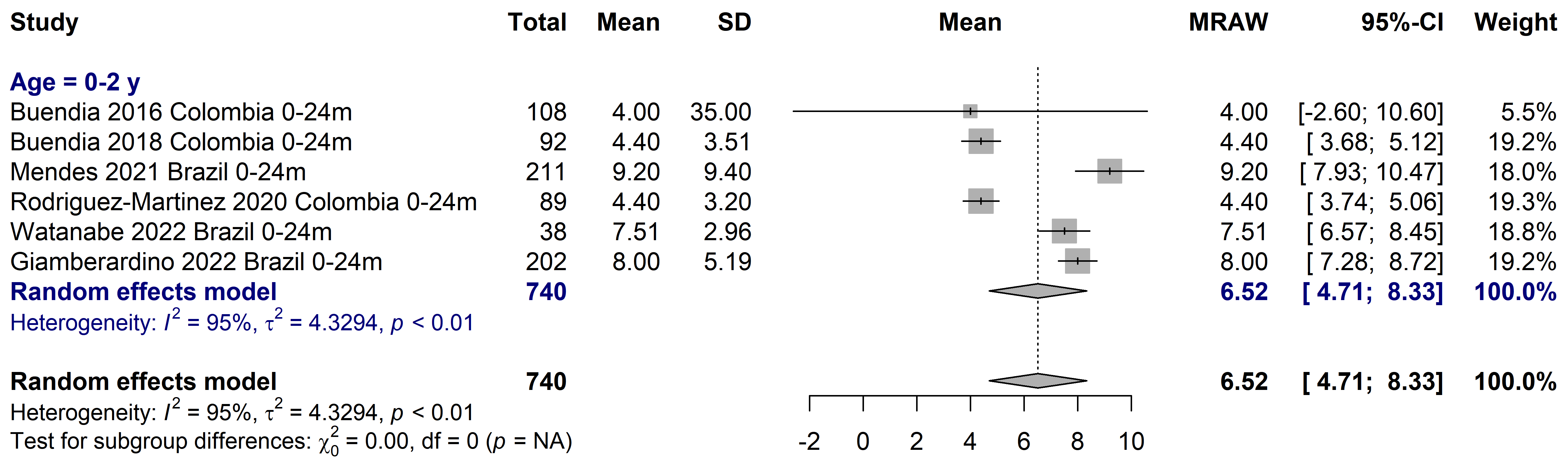
**

## Supplementary Figure 32. Length of stay in general ward (days) 0-2 years by five year calendar period (2012-2016 and 2017-2022)

**
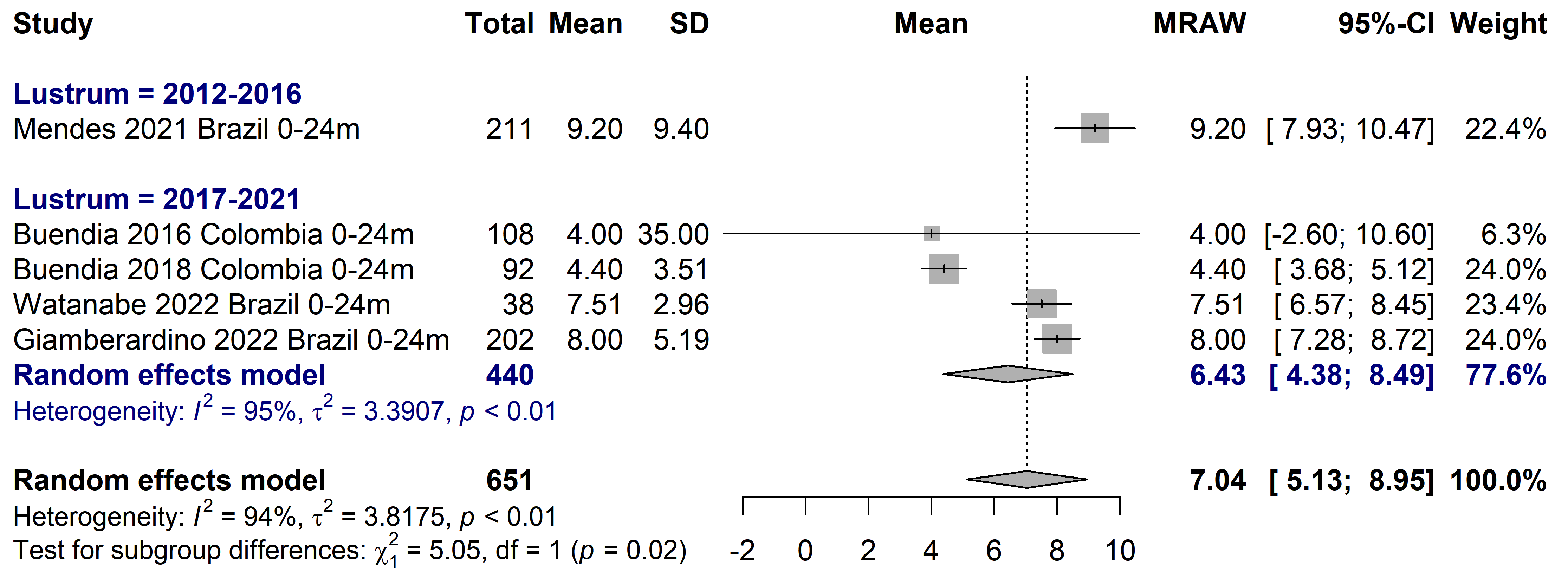
**

## Supplementary Figure 33. Antibiotic use/inpatients RSV cases 0-2 years

**
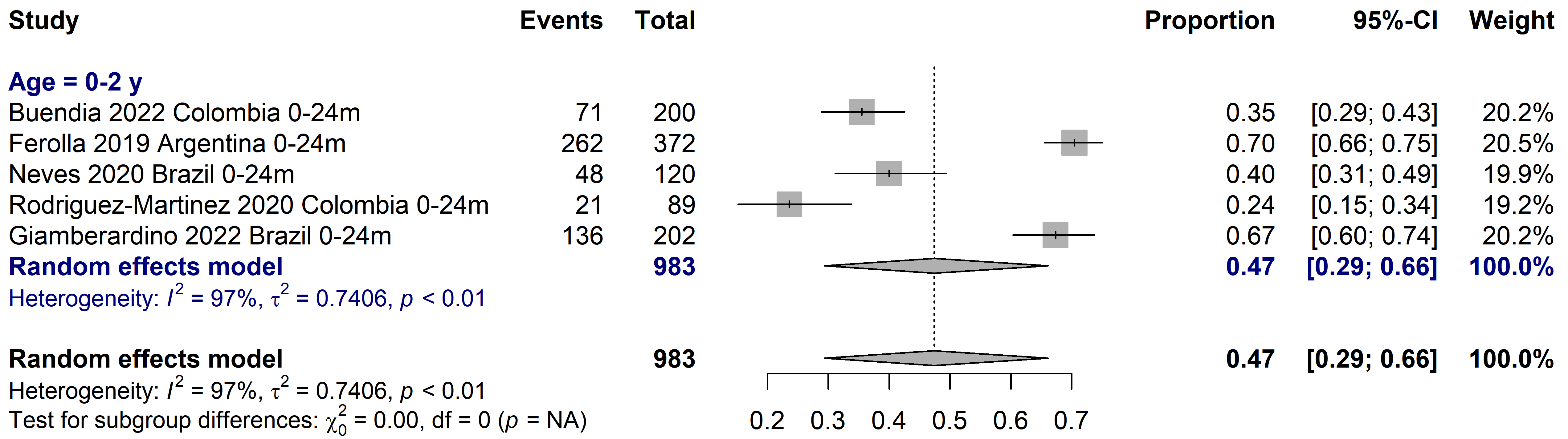
**

## Supplementary Figure 34. Antibiotic use/inpatients RSV cases 0-2 years by five year calendar period (2012-2016 and 2017-2022)

**
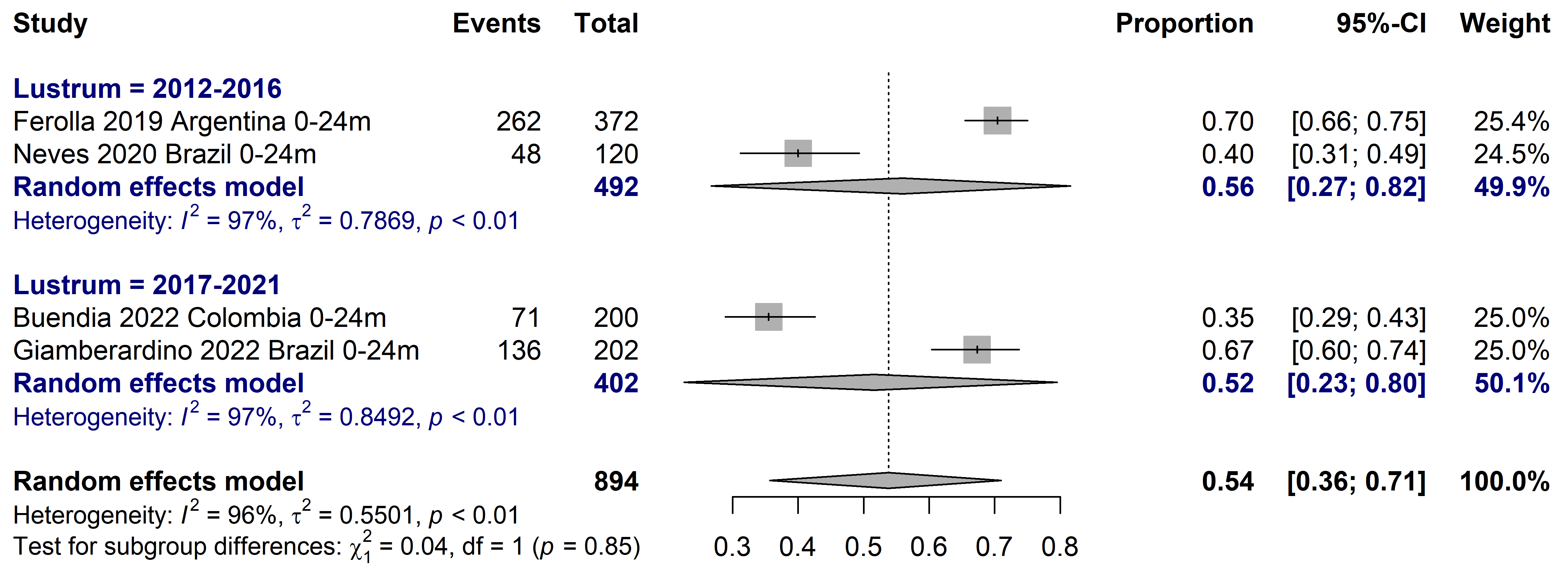
**

## Supplementary Figure 35. Invasive ventilation/RSV LRTI cases 0-2 years

**
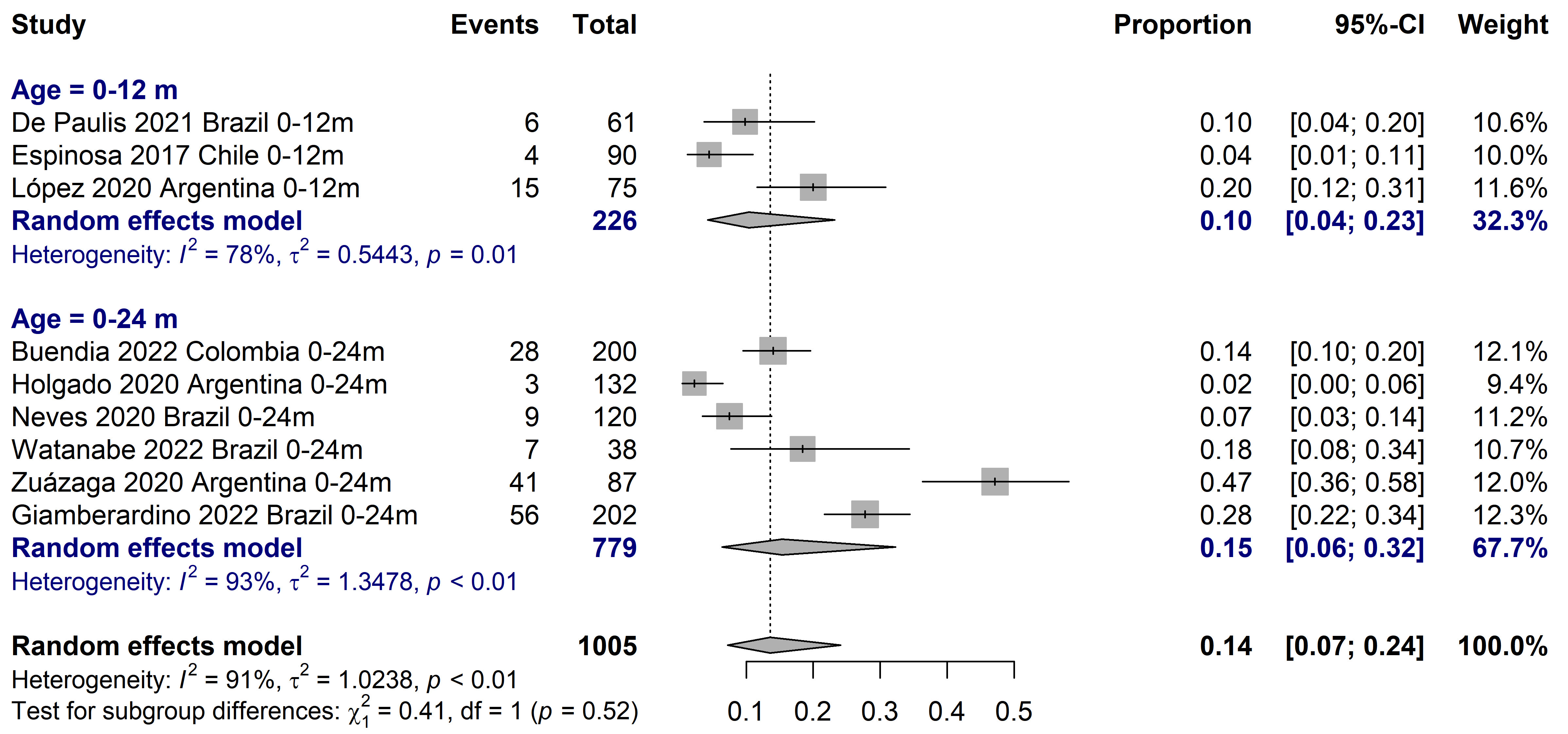
**

## Supplementary Figure 36. Invasive ventilation/RSV LRTI cases 0-2 years by five year calendar period (2012-2016 and 2017-2022)

**
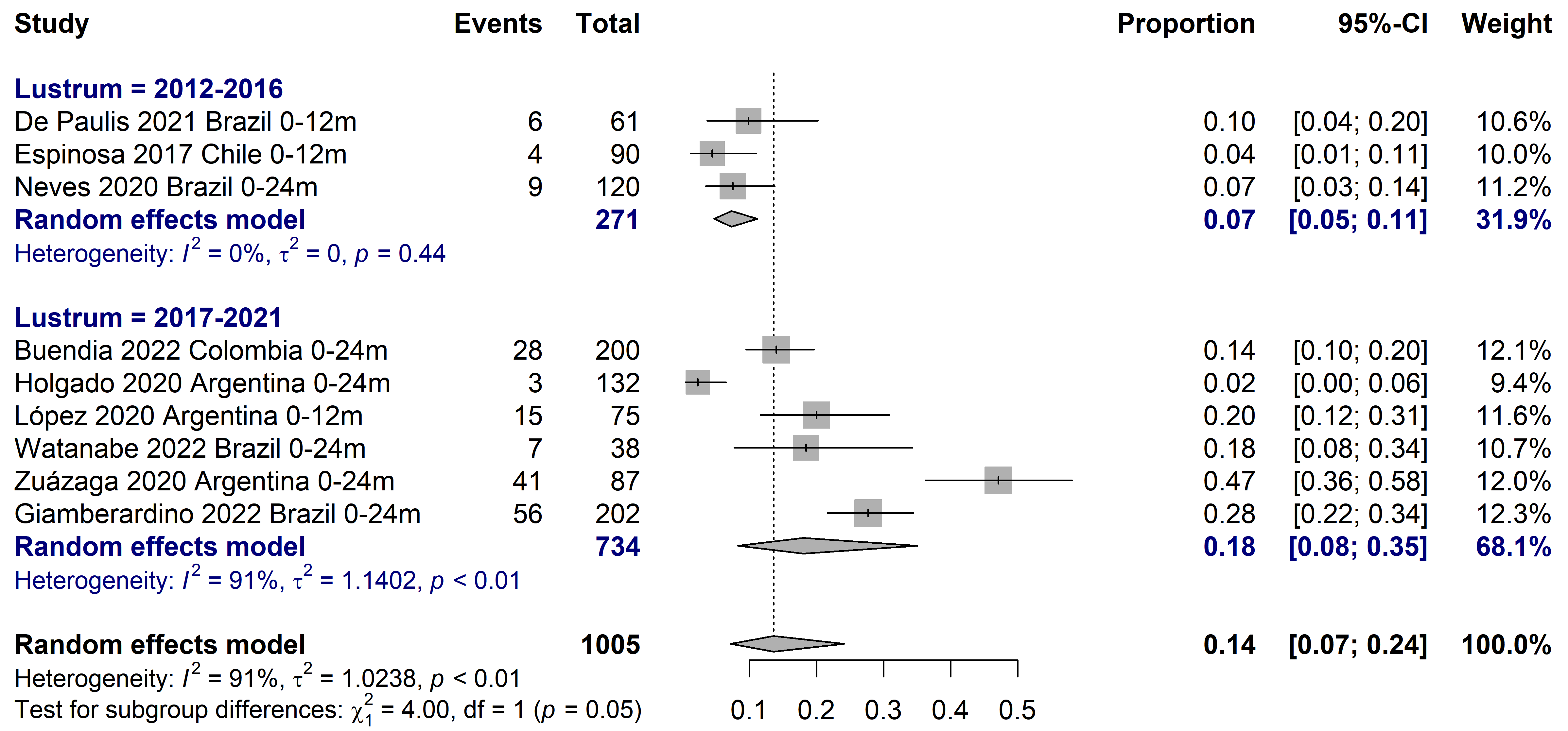
**

## Supplementary Figure 37. Invasive ventilation/RSV LRTI cases 0-2 years by low ROB

**
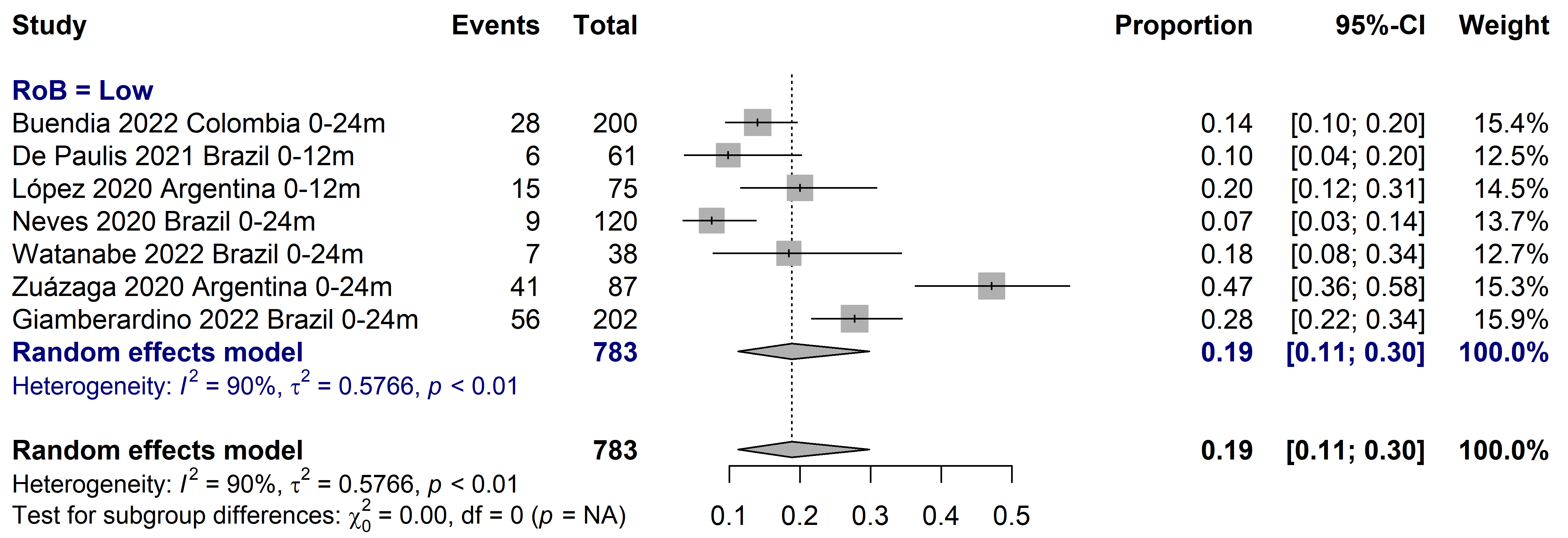
**

## Supplementary Figure 38. Invasive ventilation/RSV LRTI cases 0-5 years by five year calendar period (2012-2016 and 2017-2022)

**
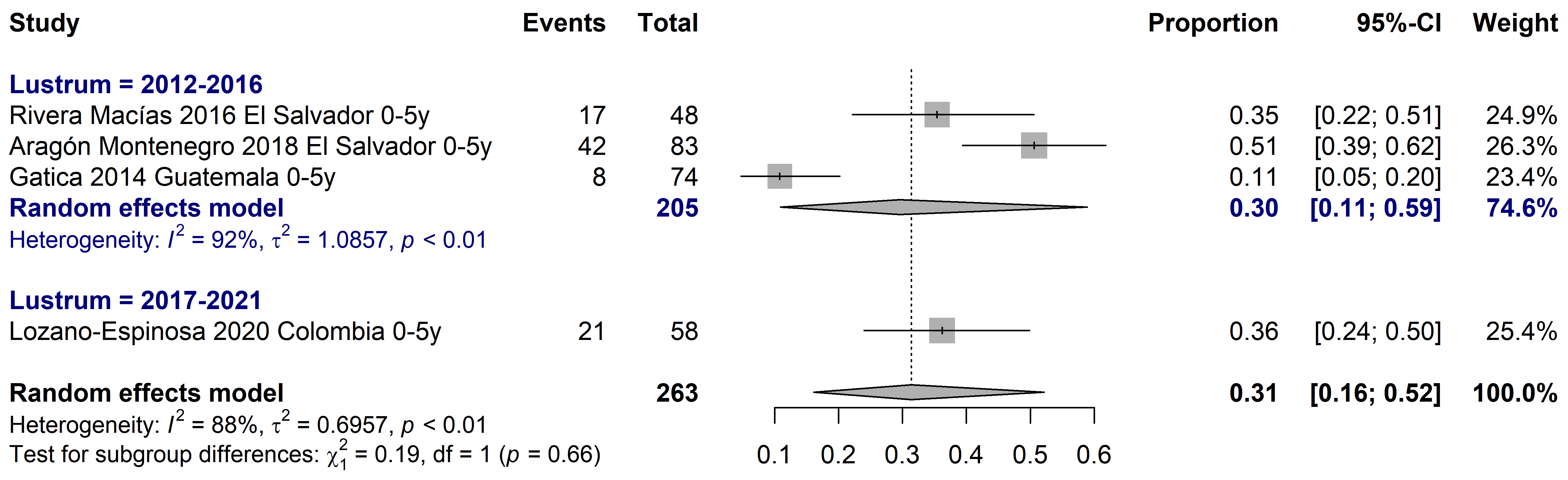
**

## Supplementary Figure 39. Invasive ventilation/RSV LRTI cases 0-5 years by low ROB

**
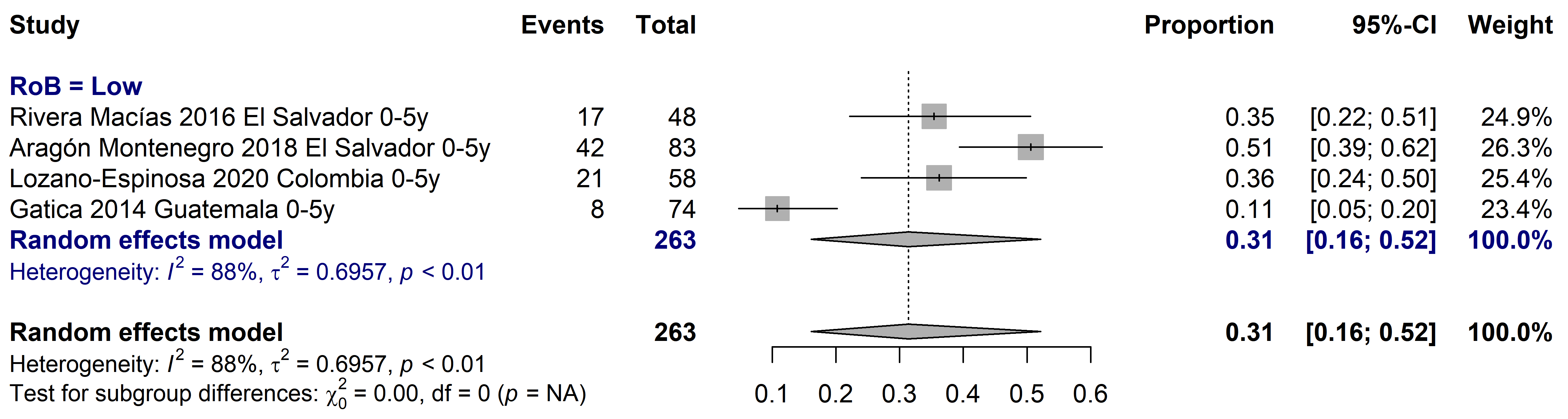
**

## Supplementary Figure 40. RSV A/viral isolates 0-2 years

**
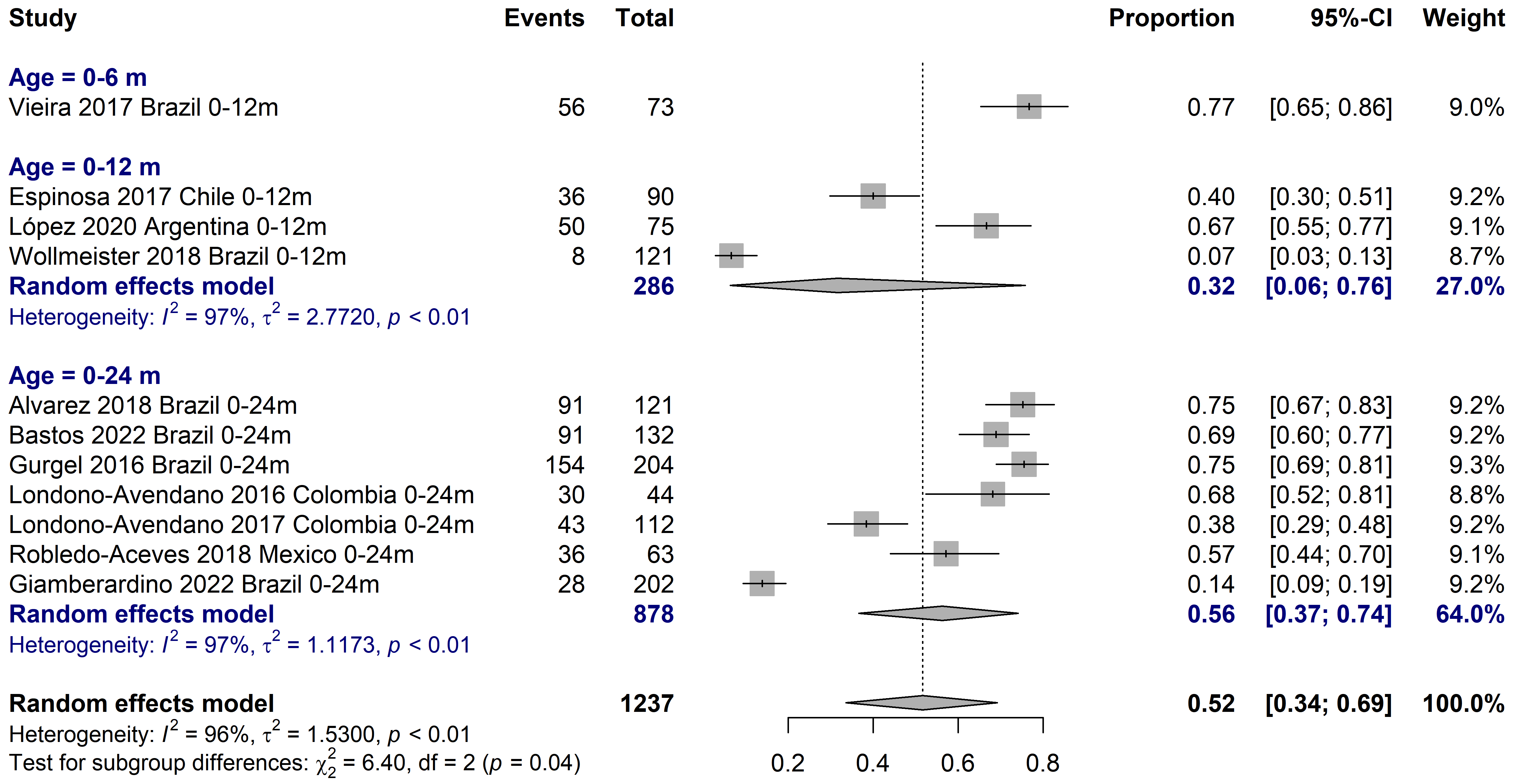
**

## Supplementary Figure 41. RSV A/viral isolates 0-2 years by five year calendar period (2012-2016 and 2017-2022)

**
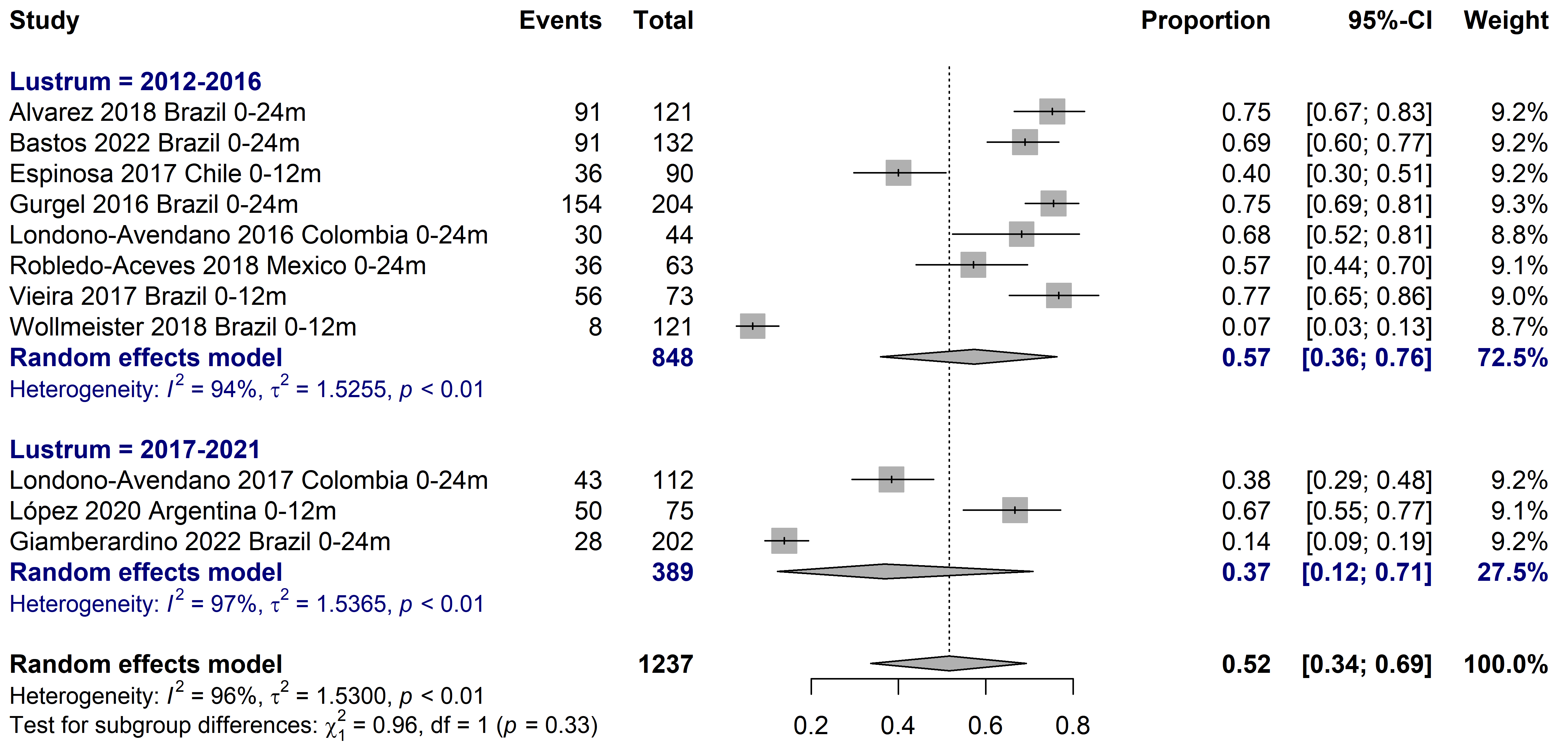
**

## Supplementary Figure 42. RSV A/viral isolates 0-2 years by low ROB

**
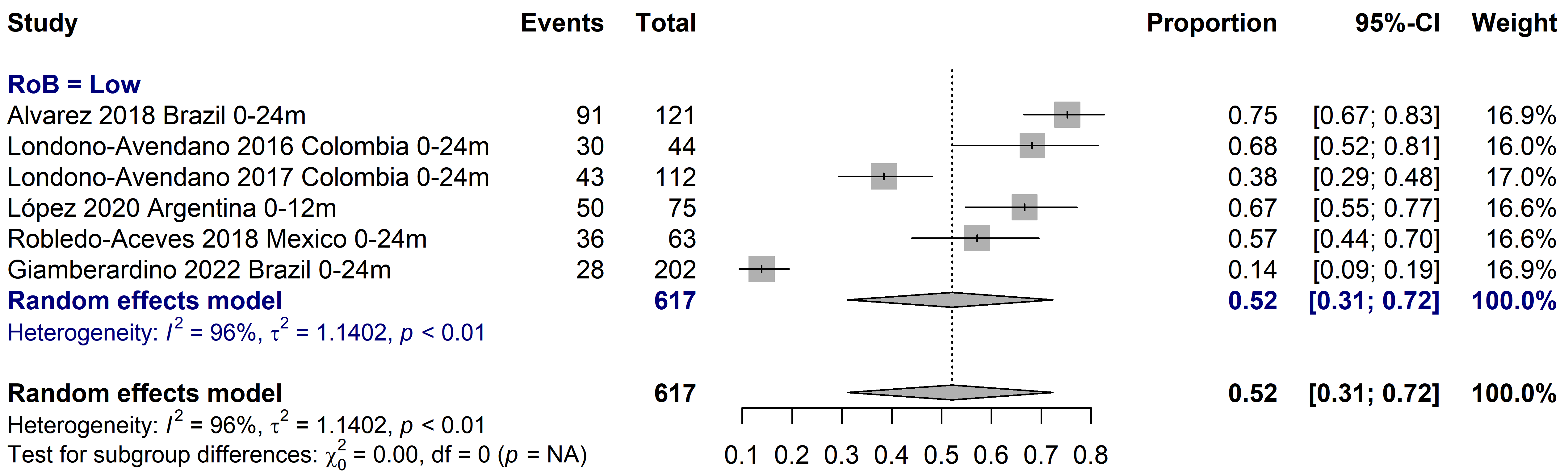
**

## Supplementary Figure 43. RSV A/viral isolates ≥65 years


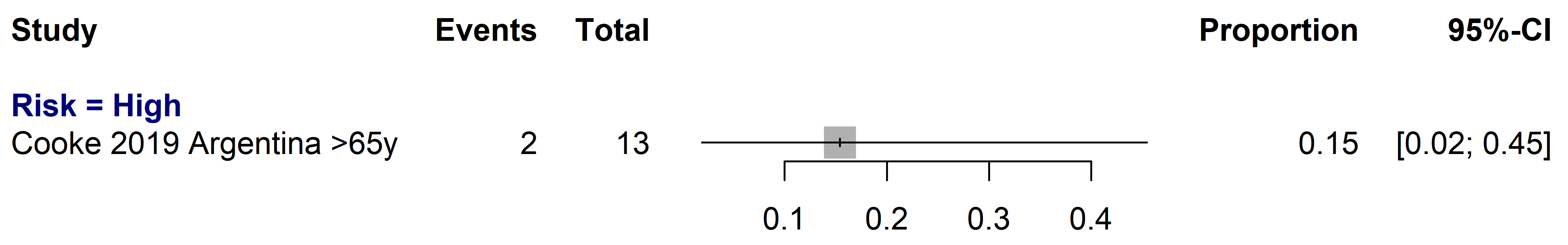


## Supplementary Figure 44. RSV B/viral isolates 0-2 years


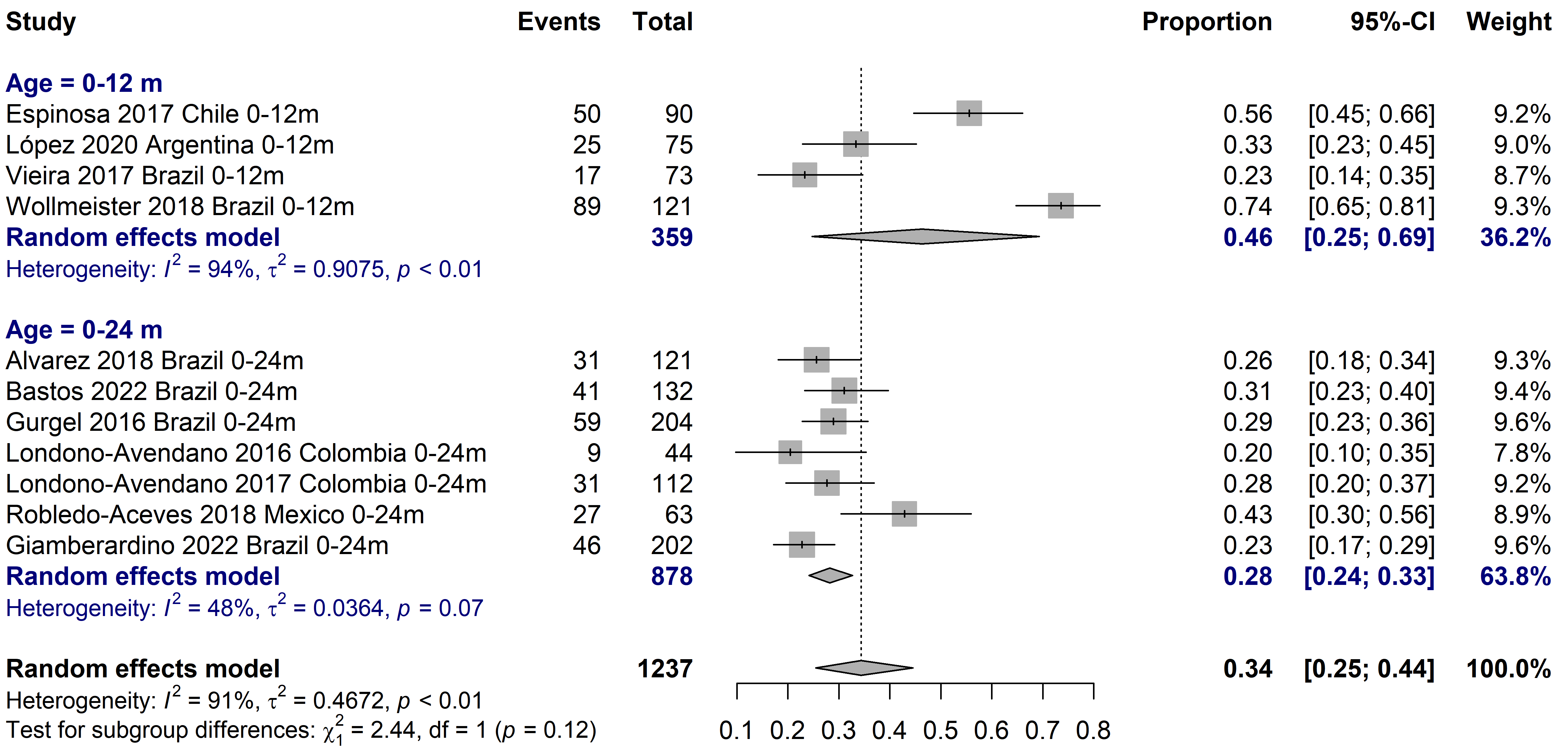


## Supplementary Figure 45. RSV B/viral isolates 0-2 years by five year calendar period (2012-2016 and 2017-2022)

**
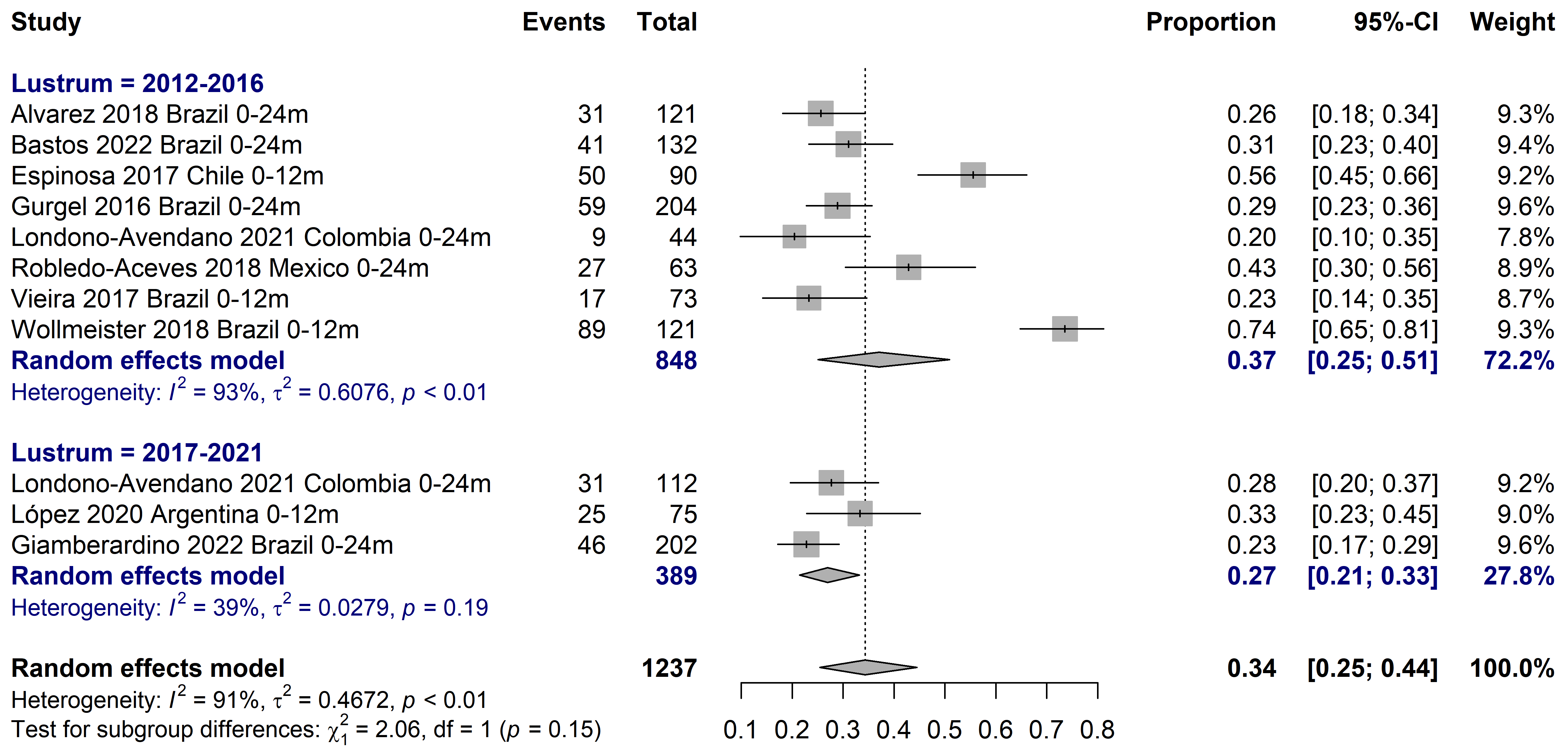
**

## Supplementary Figure 46. RSV B/viral isolates 0-2 years by low ROB

**
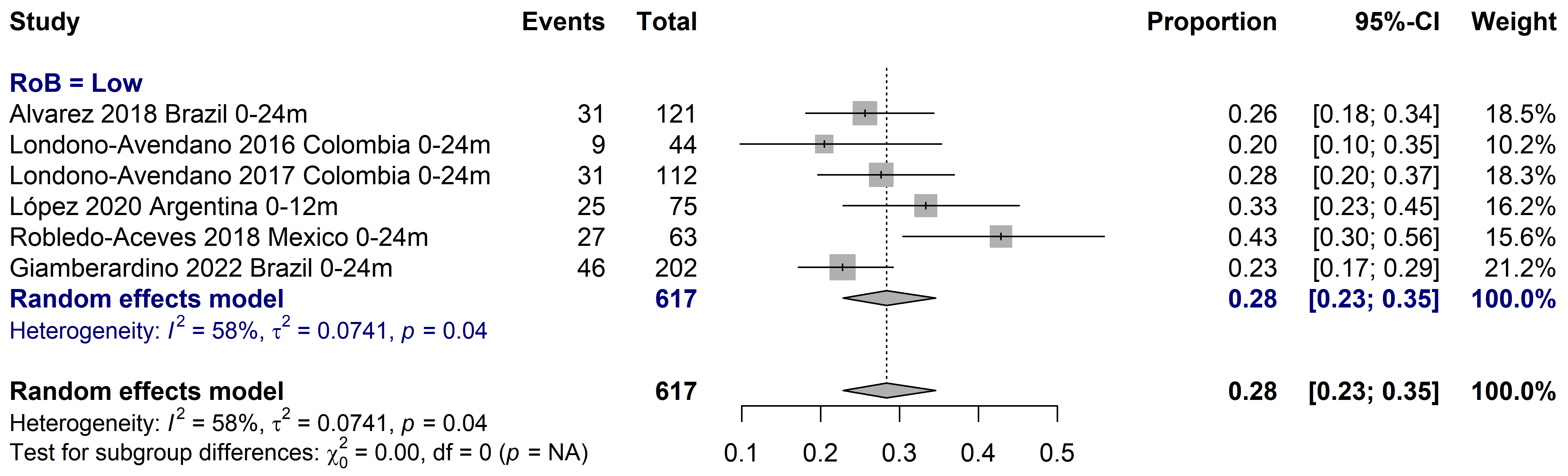
**

## Supplementary Figure 47. Viral coinfection/RSV cases 0-2 years

**
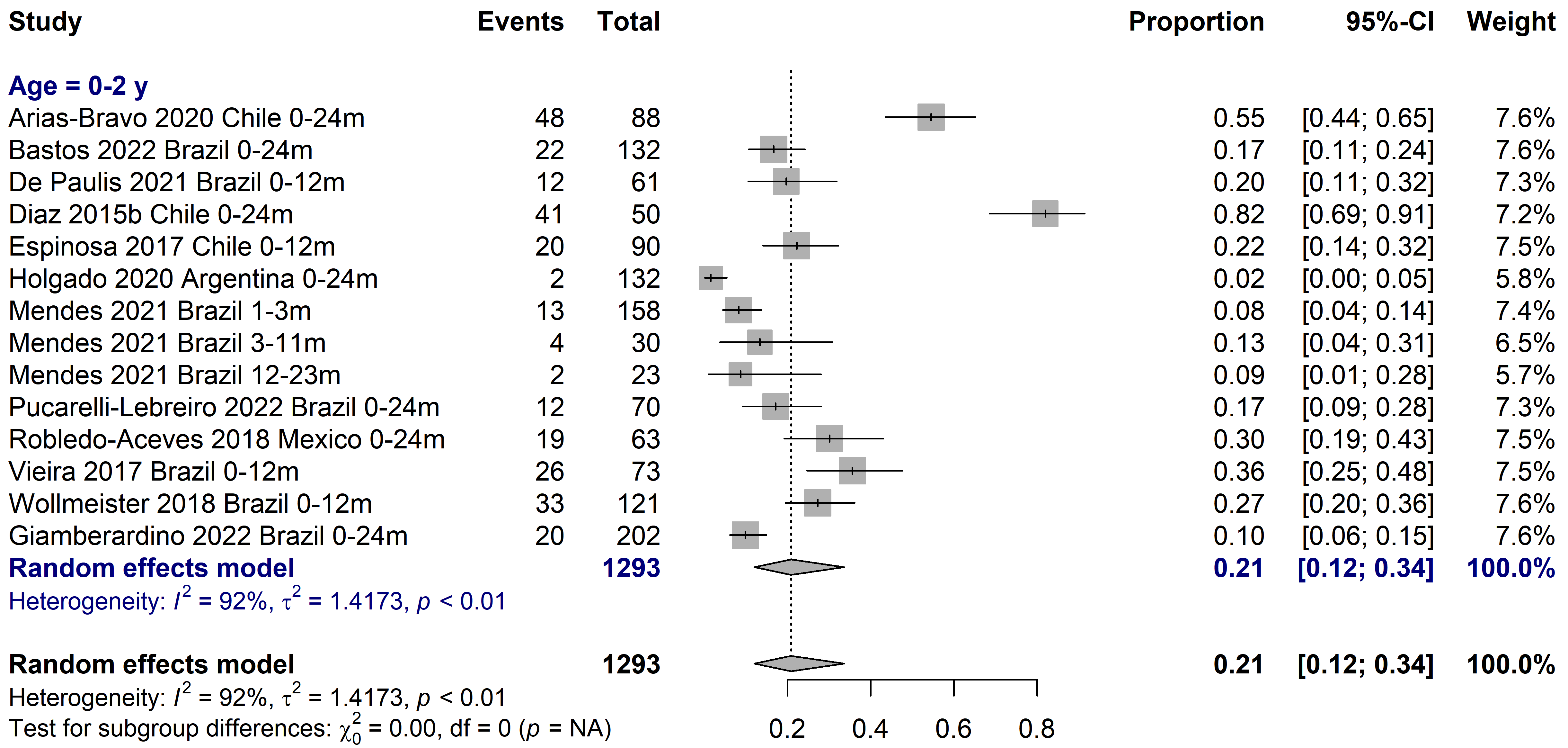
**

## Supplementary Figure 48. Viral coinfection/RSV cases 0-2 years by five year calendar period (2012-2016 and 2017-2022)

**
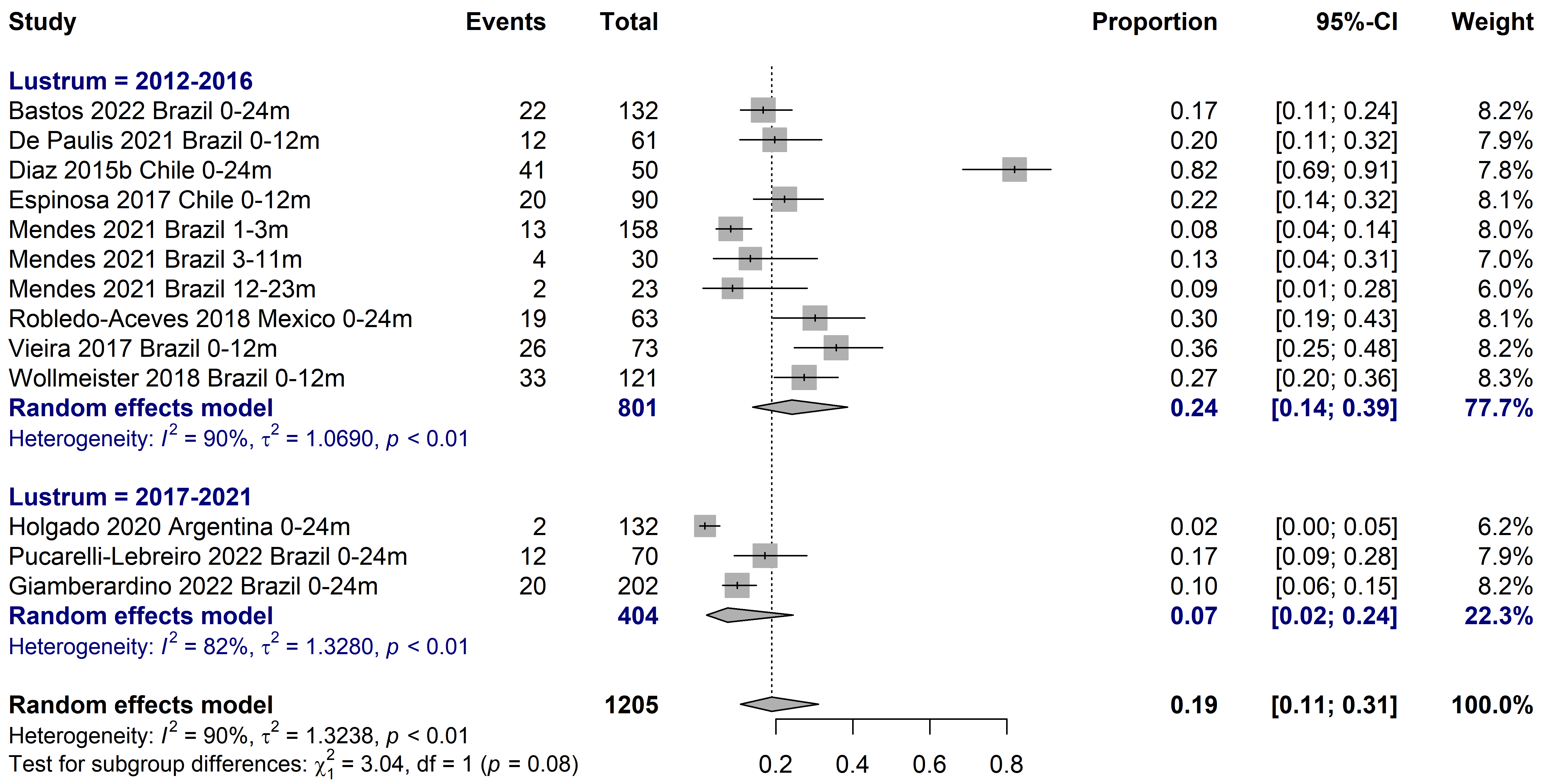
**

## Supplementary Figure 49. Viral coinfection/RSV cases 0-2 years by low ROB

**
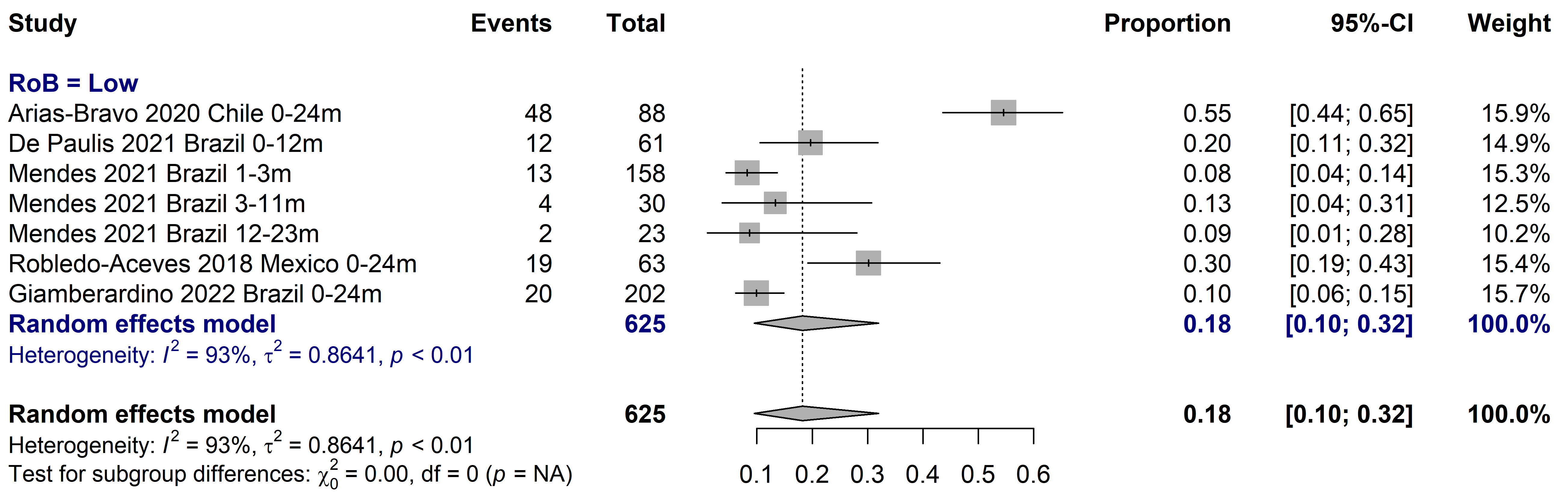
**

## Supplementary Figure 50. Viral coinfection/RSV ≥65 years

**
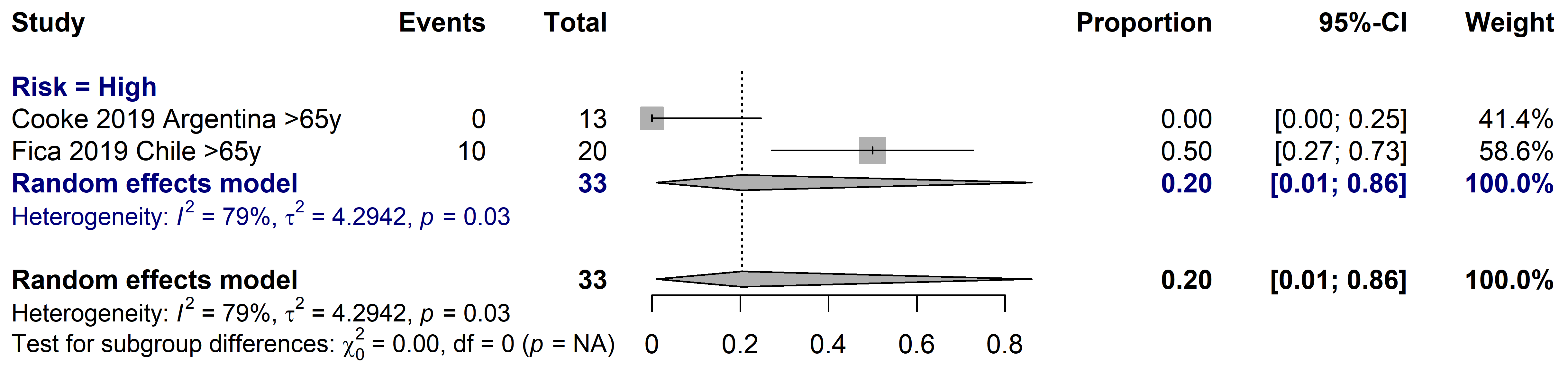
**

## Supplementary Figure 51. Bacterial coinfection/RSV cases 0-5 years by risk group (high and normal risk)

**
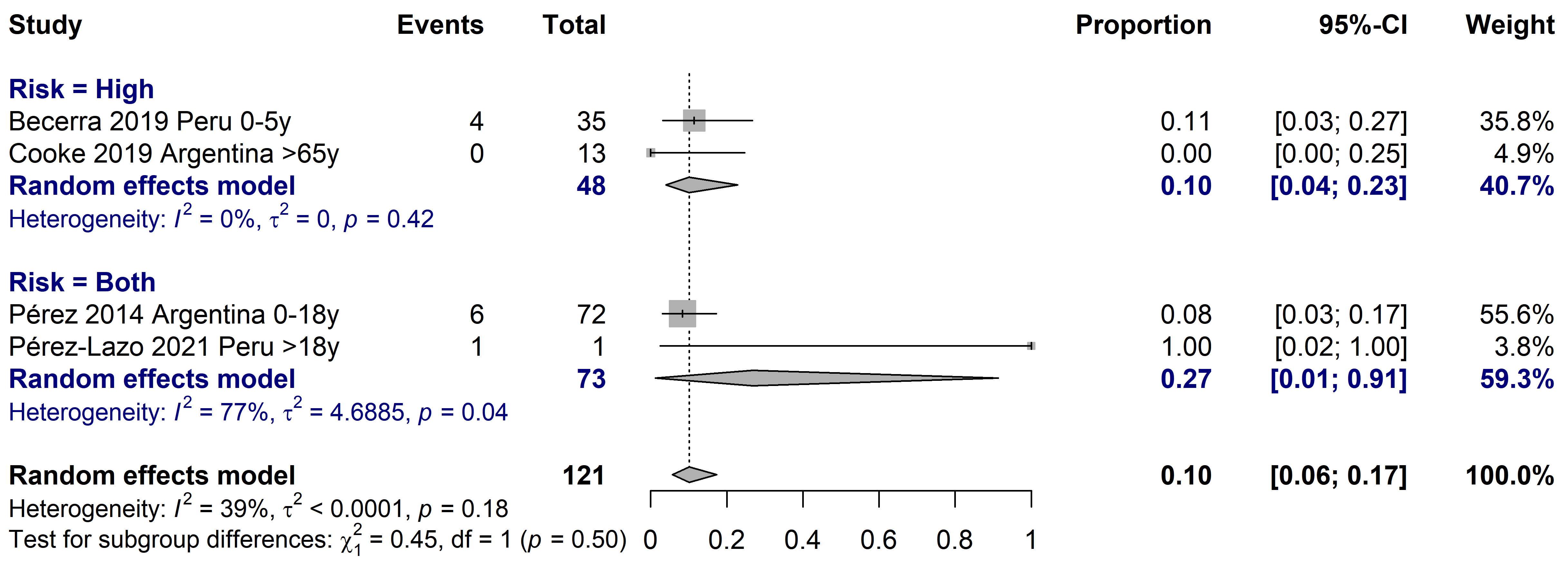
**

## Supplementary Figure 52. Bacterial coinfection/RSV cases 0-18 years

**
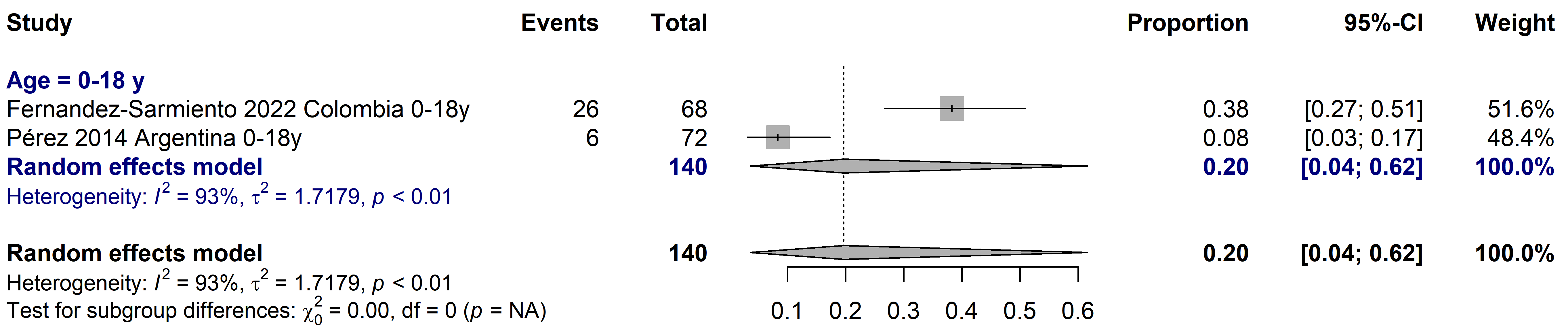
**

## References

1. Acuña D, Goya S, Nabaes Jodar MS, Grandis É, Mistchenko AS, Viegas M. RSV reemergence in Argentina since the SARS-CoV-2 pandemic. *J Clin Virol* (2022) 149:105126. doi: 10.1016/j.jcv.2022.105126

2. Andino G, Ruiz Díaz N, Espinoza J, Cóceres M, Lezcano R, Pisarello MS. Diagnóstico y vigilancia epidemiológica de virus respiratorios en la provincia de Corrientes. *Rev argent salud pública* (2017) 8:37–39. http://www.scielo.org.ar/scielo.php?script=sci_abstract&pid=S1853-810X2017000100007&lng=es&nrm=iso&tlng=es [Accessed October 1, 2023]

3. Atwell JE, Geoghegan S, Karron RA, Polack FP. Clinical Predictors of Critical Lower Respiratory Tract Illness Due to Respiratory Syncytial Virus in Infants and Children: Data to Inform Case Definitions for Efficacy Trials. *J Infect Dis* (2016) 214:1712–1716. doi: 10.1093/infdis/jiw447

4. Barbaro C, Monteverde E, Guiñazú G, Dastugue M, Mascardi N, Neira P, Golubicki A. Impacto del uso de oxigenoterapia de alto flujo en el Hospital de Niños Dr. Ricardo Gutiérrez. http://revistapediatria.com.ar/edicion-277-impacto-del-uso-oxigenoterapia-alto-flujo-hospital-ninos-dr-ricardo-gutierrez/ [Accessed October 1, 2023]

5. Baumeister E, Duque J, Varela T, Palekar R, Couto P, Savy V, Giovacchini C, Haynes AK, Rha B, Arriola CS, et al. Timing of respiratory syncytial virus and influenza epidemic activity in five regions of Argentina, 2007-2016. *Influenza Other Respi Viruses* (2019) 13:10–17. doi: 10.1111/irv.12596

6. Caballero MT, Bianchi AM, Nuño A, Ferretti AJP, Polack LM, Remondino I, Rodriguez MG, Orizzonte L, Vallone F, Bergel E, et al. Mortality Associated With Acute Respiratory Infections Among Children at Home. *J Infect Dis* (2019) 219:358–364. doi: 10.1093/infdis/jiy517

7. Castello A, Rodriguez Pérez A, Lihue Rojo G, Alvarez D, Musto A, Montali C, Sancilio A, Hamilton G, Garcia C, Sosa L, et al. Caracterización de cepas de Virus Sincitial Respiratorio en el Gran Buenos Aires y aspectos de su diseminación. *Revista Argentina de Salud Pública* (2017) 8:19–25. http://www.scielo.org.ar/scielo.php?script=sci_abstract&pid=S1853-810X2017000300004&lng=es&nrm=iso&tlng=es [Accessed October 1, 2023]

8. Cooke B, Obed M, Romandetta A, Spinelli N, Alzogaray MF, Chaparro G, Galeano L, Alcorta MB, Balbuena J, Russo M, et al. Rol del virus sincicial respiratorio en una cohorte de adultos mayores. *Actualizaciones en Sida e Infectología* (2019) doi: 10.52226/revista.v27i100.15

9. Ferolla FM, Soffe J, Mistchenko A, Contrini MM, López EL. Clinical and epidemiological impact of respiratory syncytial virus and identification of risk factors for severe disease in children hospitalized due to acute respiratory tract infection. *Arch Argent Pediatr* (2019) 117:216–223. doi: 10.5546/aap.2019.eng.216

10. Ferolla FM, Yfran EW, Ballerini MG, Caratozzolo A, Toledano A, Giordano AC, Acosta PL, Cassinelli H, Bergada I, Ropelato MG, et al. Serum Vitamin D Levels and Life-Threatening Respiratory Syncytial Virus Infection in Previously Healthy Infants. *J Infect Dis* (2022) 226:958–966. doi: 10.1093/infdis/jiac033

11. Ferrero F, Ossorio MF, Rial MJ. The return of RSV during the COVID-19 pandemic. *Pediatr Pulmonol* (2022) 57:770–771. doi: 10.1002/ppul.25802

12. Holgado MP, Raiden S, Sananez I, Seery V, De Lillo L, Maldonado LL, Kamenetzky L, Geffner J, Arruvito L. Fcγ Receptor IIa (FCGR2A) Polymorphism Is Associated With Severe Respiratory Syncytial Virus Disease in Argentinian Infants. *Front Cell Infect Microbiol* (2020) 10:607348. doi: 10.3389/fcimb.2020.607348

13. Leone MB, Ponti DA, Fernández Berengeno MN, Grisolía NA, Aprea VP, Yazde de Puleio ML, Svartz A, Haleblian E. Pesquisa de virus respiratorios habituales en pacientes pediátricos ambulatorios, a dos años de la pandemia por COVID-19. *Arch Argent Pediatr* (2022) 120: doi: 10.5546/aap.2022.264

14. López EL, Ferolla FM, Toledano A, Yfran EW, Giordano AC, Carrizo B, Feldman F, Talarico LB, Caratozzolo A, Contrini MM, et al. Genetic Susceptibility to Life-threatening Respiratory Syncytial Virus Infection in Previously Healthy Infants. *Pediatr Infect Dis J* (2020) 39:1057–1061. doi: 10.1097/INF.0000000000002827

15. Lucion MF, Juarez M del V, Viegas M, Castellano V, Romanin VS, Grobaporto M, Bakir J, Mistchenko AS, Gentile A. Respiratory syncytial virus: clinical and epidemiological pattern in pediatric patients admitted to a children’s hospital between 2000 and 2013. *Arch Argent Pediatr* (2014) 112:397–404. doi: 10.5546/aap.2014.eng.397

16. Orqueda AS, Lucion MF, Juárez M del V, Barquez R, Stach P, Nievas A, Losi LF, Suárez RF, Romero Bollón L, Pejito MN, et al. Vigilancia de virus sincicial respiratorio e influenza en niños escolarizados asistidos en un hospital pediátrico durante 2 meses del segundo semestre de 2021. *Arch Argent Pediatr* (2022) 120: doi: 10.5546/aap.2022.269

17. Pérez G, Rosanova MT, Taicz M, Inda L, Martirén S, García Escude N, Casimir L, Viale D, Hernández C, Bologna R. Neumonía de la comunidad en pediatría: Estudio descriptivo en un hospital de alta complejidad. *Med Infant* (2014)90–96. http://www.medicinainfantil.org.ar/images/stories/volumen/2014/xxi_2_090.pdf [Accessed October 1, 2023]

18. Rojo GL, Goya S, Orellana M, Sancilio A, Rodriguez Perez A, Montali C, García C, Sosa L, Musto A, Alvarez D, et al. Unravelling respiratory syncytial virus outbreaks in Buenos Aires, Argentina: Molecular basis of the spatio-temporal transmission. *Virology* (2017) 508:118–126. doi: 10.1016/j.virol.2017.04.030

19. Russo M, Avaro MM, Czech A, Benedetti E, Pardón F, Campos AM, Macias EM, Pontoriero A, Baumeister E. Epidemiology and molecular characterization of Respiratory Syncytial Virus in Argentina 2017. *Int J Infect Dis* (2018) 73:200. doi: 10.1016/j.ijid.2018.04.3868

20. Viegas M, Goya S, Mistchenko AS. Sixteen years of evolution of human respiratory syncytial virus subgroup A in Buenos Aires, Argentina: GA2 the prevalent genotype through the years. *Infect Genet Evol* (2016) 43:213–221. doi: 10.1016/j.meegid.2016.04.034

21. Zuázaga DM, Pellegrini S, Gallagher R, Casais G, Landry L. Evolución de pacientes con bronquiolitis tratados con cánulas de alto flujo de oxígeno que ingresan a la unidad de cuidados intensivos. https://www.medicinainfantil.org.ar/index.php/2020-volumen-xxvii/numero-2/565-evolucion-de-pacientes-con-bronquiolitis-tratados-con-canulas-de-alto-flujo-de-oxigeno-que-ingresan-a-la-unidad-de-cuidados-intensivos [Accessed October 1, 2023]

22. Chavez D, Gonzales-Armayo V, Mendoza E, Palekar R, Rivera R, Rodriguez A, Salazar C, Veizaga A, Añez A. Estimation of influenza and respiratory syncytial virus hospitalizations using sentinel surveillance data-La Paz, Bolivia. 2012-2017. *Influenza Other Respi Viruses* (2019) 13:477–483. doi: 10.1111/irv.12663

23. Gareca Perales J, Soleto Ortiz L, Loayza Mafayle R, Machuca Soto B, Hidalgo Flores L, López Montaño J, Zuna Calle N, Jarovsky D, Naaman Berezin E. Diagnosis of Community-acquired Pneumonia in Hospitalized Children: A Multicenter Experience in Bolivia. *Pediatr Infect Dis J* (2021) 40:32–38. doi: 10.1097/INF.0000000000002909

24. Alvarez AE, Marson FAL, Bertuzzo CS, Bastos JCS, Baracat ECE, Brandão MB, Tresoldi AT, das Neves Romaneli MT, Almeida CCB, de Oliveira T, et al. Association between single nucleotide polymorphisms in TLR4, TLR2, TLR9, VDR, NOS2 and CCL5 genes with acute viral bronchiolitis. *Gene* (2018) 645:7–17. doi: 10.1016/j.gene.2017.12.022

25. Araujo Castro I, Costa LDC, Oliveira ACR, Souza M, das Dôres de Paula Cardoso D, Camargos PAM, Costa PSS, Fiaccadori FS. Circulation profile of respiratory viruses in symptomatic and asymptomatic children from Midwest Brazil. *Braz J Microbiol* (2020) 51:1729–1735. doi: 10.1007/s42770-020-00368-0

26. Azevedo TR, Kern LB, Polese-Bonatto M, Sartor ITS, Varela FH, Fernandes IR, Zavaglia GO, Giannini GLT, Cordero EAA, Santos AP, et al. Prevalencia de agentes infecciosos respiratorios em adultos hospitalizados durante a pandemia de COVID-19 em 2020. *Braz J Infect Dis* (2022) 26:102058. doi: 10.1016/j.bjid.2021.102058

27. Bamberg EL, Nogueira RL, Pinheiro DA de A, Silva CM, Paschoalini S. Mudança no padrão de vírus respiratórios identificados em uma população pediátrica hospitalizada durante os anos de 2019 e 2020. *Rev Assoc Med Minas Gerais* (2021) 31: doi: 10.5935/2238-3182.2021e31112

28. Bastos JCS, Simas PVM, Caserta LC, Bragunde AEA, Marson FA de L, Martini MC, Padilla MA, Ribeiro JD, Santos MMABD, Arns CW. Rhinoviruses as critical agents in severe bronchiolitis in infants. *J Pediatr*  (2022) 98:362–368. doi: 10.1016/j.jped.2021.11.006

29. Boschiero MN, Duarte A, Palamim CVC, Alvarez AE, Mauch RM, Marson FAL. Frequency of respiratory pathogens other than SARS-CoV-2 detected during COVID-19 testing. *Diagn Microbiol Infect Dis* (2022) 102:115576. doi: 10.1016/j.diagmicrobio.2021.115576

30. Camargo Costa LD, Camargos PAM, Brand PLP, Fiaccadori FS, de Lima Dias E Souza MB, das Dôres de Paula Cardoso D, de Araújo Castro Í, Minamisava R, Sucasas da Costa PS. Asthma exacerbations in a subtropical area and the role of respiratory viruses: a cross-sectional study. *BMC Pulm Med* (2018) 18:109. doi: 10.1186/s12890-018-0669-6

31. Cesar R, Bispo B, Felix PH, Modolo MC, Cabó S, Souza A, Pizzini P, Horigoshi N, Rotta A. A randomized controlled trial of high-flow nasal cannula versus CPAP in critical bronchiolitis. *Crit Care Med* (2018) 46:553. doi: 10.1097/01.ccm.0000529144.79628.12

32. De Paulis M, Oliveira DBL, Thomazelli LM, Ferraro AA, Durigon EL, Vieira SE. The importance of viral load in the severity of acute bronchiolitis in hospitalized infants. *Clinics*  (2021) 76:e3192. doi: 10.6061/clinics/2021/e3192

33. Faggion HZ, Leotte J, Trombetta H, Pereira LA, Lapinski BA, Nogueira MB, Vidal LR, Almeida BM, Petterle RR, Raboni SM. Influenza Sentinel Surveillance and Severe Acute Respiratory Infection in a Reference Hospital in Southern Brazil. *Rev Soc Bras Med Trop* (2019) 53:e20170498. doi: 10.1590/0037-8682-0498-2017

34. Ferlini R, Pinheiro FO, Andreolio C, Carvalho PRA, Piva JP. Characteristics and progression of children with acute viral bronchiolitis subjected to mechanical ventilation. *Rev Bras Ter Intensiva* (2016) 28:55–61. doi: 10.5935/0103-507X.20160003

35. Freitas F, Silva C. Respiratory viruses in infants in a pediatric sentinel surveillance unit, Brazil. *Int J Infect Dis* (2019) 79:101. doi: 10.1016/j.ijid.2018.11.252

36. Furlan TM. Estudo da viabilidade para introduzir na rotina testes de diagnóstico para infecção respiratória aguda. (2016). http://www.teses.usp.br/teses/disponiveis/5/5144/tde-30062016-161948/publico/TeresaMariaFurlanVersaoCorrigida.pdf [Accessed October 1, 2023]

37. Giamberardino HIG, Pacheco APO, Pereira LA, Debur M do C, Genehold G, Raboni SM. Respiratory syncytial virus: host genetic susceptibility and factors associated with disease severity in a cohort of pediatric patients. *J Trop Pediatr* (2022) 68: doi: 10.1093/tropej/fmac091

38. Gurgel RQ, Bezerra PG de M, Duarte M do CMB, Moura AÁ, Souza EL, Silva LS da S, Suzuki CE, Peixoto RB. Relative frequency, Possible Risk Factors, Viral Codetection Rates, and Seasonality of Respiratory Syncytial Virus Among Children With Lower Respiratory Tract Infection in Northeastern Brazil. *Medicine*  (2016) 95:e3090. doi: 10.1097/MD.0000000000003090

39. Holtman Ferreira L, Silva BG da, Giamberardino HI, Pacheco AP, Pereira LA, Genelhoud G, Petterle RR, Raboni SM. The association of breastfeeding and other factors on respiratory virus positivity and severity in hospitalized children. *Microbiol Immunol* (2022) 66:216–224. doi: 10.1111/1348-0421.12969

40. Lomar FP, Alencar R, Serpa Neto A, Correa TD, Pinho JR, Pasternak J, Barbas CSV. “Viral Respiratory Infections in Hospitalized Symptomatic Respiratory Patients.,” *C63. VIRAL RESPIRATORY INFECTIONS*. American Thoracic Society International Conference Abstracts. American Thoracic Society (2017). p. A6064–A6064 doi: 10.1164/ajrccm-conference.2017.195.1_MeetingAbstracts.A6064

41. Medeiros DNM, Mafra ACCN, Souza DC de, Troster EJ. Epidemiology and treatment of sepsis at a public pediatric emergency department. *Einstein* (2022) 20:eAO6131. doi: 10.31744/einstein_journal/2022AO6131

42. Mendes ET, Paranhos HL, Santos ICM, Souza LB de, Aquino JLB de, Leandro-Merhi VA, Silva RV da, Lima MPJS. Prognosis of hospitalized children under 2 years of age with co-detection of influenza A and respiratory syncytial virus at the healthcare facility. *Rev Bras Saude Mater Infant* (2021) 21:531–537. doi: 10.1590/1806-93042021000200010

43. Moreira FB, Rosario CS, Santos JS, Avanzi VM, Nogueira MB, Vidal LR, Raboni SM. Molecular characterization and clinical epidemiology of human respiratory syncytial virus (HRSV) A and B in hospitalized children, Southern Brazil. *J Med Virol* (2017) 89:1489–1493. doi: 10.1002/jmv.24795

44. Neves KC, Vieira SE. Conditions of vulnerability to the inadequate treatment of bronchiolitis. *Rev Assoc Med Bras* (2020) 66:187–193. doi: 10.1590/1806-9282.66.2.187

45. Ocadaque CJ. Aspectos clínicos e epidemiológicos de pneumonias infantis associadas aos quatro tipos de vírus para influenza em Fortaleza-CE. (2015) http://www.repositorio.ufc.br/handle/riufc/15381 [Accessed October 2, 2023]

46. Pinheiro Lopes G, Amorim ÍPS, Melo B de O de, Maramaldo CEC, Bomfim MRQ, Lima Neto LG, Alves MS, Silva FB, Soeiro-Pereira PV, Falcai A. Identification and seasonality of rhinovirus and respiratory syncytial virus in asthmatic children in tropical climate. *Biosci Rep* (2020) 40: doi: 10.1042/BSR20200634

47. Piva J, Ferlini R, Pinheiro F, Baldasso E, Andreolio C, Lago P, Giorgio C, Carvalho P, Trotta E. High survival rate is the rule for children with acute viral bronchiolitis submitted to mechanical ventilation. *Pediatr Crit Care Med* (2014) 15:105. doi: 10.1097/01.pcc.0000449185.12949.6b

48. Pucarelli-Lebreiro G, Venceslau MT, Cordeiro CC, Maciel FQ, Anachoreta TD, de Abreu TF, Frota ACC, Castiñeiras TMPP, da Costa AM, Lopes AC da L, et al. Clinical Manifestations and Complications of Children With COVID-19 Compared to Other Respiratory Viral Infections: A Cohort Inpatient Study From Rio de Janeiro, Brazil. *Front Pediatr* (2022) 10:934648. doi: 10.3389/fped.2022.934648

49. Rodrigues de Araujo KL, Aquino ÉC de, Silva LLS da, Ternes YMF. Factors associated with Severe Acute Respiratory Syndrome in a Brazilian central region. *Cien Saude Colet* (2020) 25:4121–4130. doi: 10.1590/1413-812320202510.2.26802020

50. Santos R-KO, Borges IC, Souza ML, Bouzas ML, Nascimento-Carvalho CM. Seasonality of distinct respiratory viruses in a tropical city: implications for prophylaxis. *Trop Med Int Health* (2021) 26:672–679. doi: 10.1111/tmi.13571

51. Serra Campelo F, de Aguiar Cordeiro R, Moura FEA. The role of human bocavirus as an agent of community-acquired pneumonia in children under 5 years of age in Fortaleza, Ceará (Northeast Brazil). *Braz J Microbiol* (2022) 53:1915–1924. doi: 10.1007/s42770-022-00806-1

52. Silva AMCE, Lima JL, Virolli J, Vidal P, Quental B, Scota S, Schunck A, Franca Silva ILA. Prevalence of respiratory viruses in immunosupressed patients in an oncological hospital in Sao Paulo-Brazil. *Antimicrob Resist Infect Control* (2017) 6:1–162. doi: 10.1186/s13756-017-0201-4

53. Sobrinho FLDV, Aragon DC, Carlotti APCP. Epidemiology and factors associated with the severity of viral acute lower respiratory infection in children hospitalized in Manaus, Amazonas, in 2017-2018: An observational study. *Medicine*  (2021) 100:e25799. doi: 10.1097/MD.0000000000025799

54. Stein RT. The COVID-19 Pandemic and Its Effect in Brazil. *Pediatr Pulmonol* (2021)S33–S34. https://pesquisa.bvsalud.org/global-literature-on-novel-coronavirus-2019-ncov/resource/pt/covidwho-1292506 [Accessed October 2, 2023]

55. Tabor DE, Fernandes F, Langedijk AC, Wilkins D, Lebbink RJ, Tovchigrechko A, Ruzin A, Kragten-Tabatabaie L, Jin H, Esser MT, et al. Global Molecular Epidemiology of Respiratory Syncytial Virus from the 2017−2018 INFORM-RSV Study. *J Clin Microbiol* (2020) 59:10.1128/jcm.01828-20. doi: 10.1128/jcm.01828-20

56. Varela FH, Sartor ITS, Polese-Bonatto M, Azevedo TR, Kern LB, Fazolo T, de David CN, Zavaglia GO, Fernandes IR, Krauser JRM, et al. Rhinovirus as the main co-circulating virus during the COVID-19 pandemic in children. *J Pediatr*  (2022) 98:579–586. doi: 10.1016/j.jped.2022.03.003

57. Vianna LA, Siqueira MM, Volpini LPB, Louro ID, Resende PC. Seasonality, molecular epidemiology, and virulence of Respiratory Syncytial Virus (RSV): A perspective into the Brazilian Influenza Surveillance Program. *PLoS One* (2021) 16:e0251361. doi: 10.1371/journal.pone.0251361

58. Vieira SE, Thomazelli LM, de Paulis M, Ferronato AE, Oliveira DB, Martinez MB, Durigon EL. Infections Caused by HRSV A ON1 Are Predominant among Hospitalized Infants with Bronchiolitis in São Paulo City. *Biomed Res Int* (2017) 2017:3459785. doi: 10.1155/2017/3459785

59. Watanabe RAS, Cruz JS, de Souza Luna LK, Alves VRG, Conte DD, Lyra L, Nishiyama F, Camargo BS, Bellei N. Respiratory syncytial virus: viral load, viral decay, and disease progression in children with bronchiolitis. *Braz J Microbiol* (2022) 53:1241–1247. doi: 10.1007/s42770-022-00742-0

60. Wollmeister E, Alvarez AE, Bastos JCS, Marson FAL, Ribeiro JD, Baracat ECE, Arns CW, Riccetto AGL. Respiratory syncytial virus in Brazilian infants - Ten years, two cohorts. *J Clin Virol* (2018) 98:33–36. doi: 10.1016/j.jcv.2017.12.002

61. Yamaguto GE, Zhen F, Moreira MM, Montesanti BM, Raboni SM. Community respiratory viruses and healthcare-associated infections: epidemiological and clinical aspects. *J Hosp Infect* (2022) 122:187–193. doi: 10.1016/j.jhin.2022.01.009

62. Arias-Bravo G, Valderrama G, Inostroza J, Reyes-Farías M, Garcia-Diaz DF, Zorondo-Rodríguez F, Fuenzalida LF. Overnutrition in Infants Is Associated With High Level of Leptin, Viral Coinfection and Increased Severity of Respiratory Infections: A Cross-Sectional Study. *Front Pediatr* (2020) 8:44. doi: 10.3389/fped.2020.00044

63. Bertrand PJ, Vázquez Y, Beckhaus AA, González LA, Contreras AM, Ferrés M, Padilla O, Riedel CA, Kalergis AM, Bueno SM. Identification of biomarkers for disease severity in nasopharyngeal secretions of infants with upper or lower respiratory tract viral infections. *Clin Exp Immunol* (2022) 210:68–78. doi: 10.1093/cei/uxac083

64. Corvalán L. P, Arias B. G, Morales S. P, González M. R, Inostroza S. J, Fuenzalida I. L. Inmunofluorescencia indirecta versus reacción de polimerasa en cadena para el diagnóstico de virus respiratorios en niños ingresados en un hospital de la Región Metropolitana. *Rev Chilena Infectol* (2019) 36:26–31. doi: 10.4067/S0716-10182019000100026

65. Espinosa Y, San Martín C, Torres AA, Farfán MJ, Torres JP, Avadhanula V, Piedra PA, Tapia LI. Genomic Loads and Genotypes of Respiratory Syncytial Virus: Viral Factors during Lower Respiratory Tract Infection in Chilean Hospitalized Infants. *Int J Mol Sci* (2017) 18: doi: 10.3390/ijms18030654

66. Diaz P, Reynolds H, Zepeda G, Gaggero A, Rivas M, Uasapud P, Pinto R, Boza ML. Detection of Hemophilus Influenzae in children with respiratory syncytial virus and rhinovirus lower respiratory tract infection. *Eur Respir J* (2015) 46: doi: 10.1183/13993003.congress-2015.PA3623

67. Fica A, Pinto F, Sotomayor V, Fasce R, Andrade W, Dabanch J, Soto A, Triantafilo V. Host characteristics predict outcome among adult patients admitted by severe acute respiratory infection. *Rev Med Chil* (2019) 147:842–851. doi: 10.4067/S0034-98872019000700842

68. Le-Corre N, Pérez R, Vizcaya C, Martínez-Valdebenito C, López T, Monge M, Alarcón R, Moller F, Martínez M-T, Massardo J-M, et al. Relevance of codetection of respiratory viruses in the severity of acute respiratory infection in hospitalized children. *Andes Pediatr* (2021) 92:349–358. doi: 10.32641/andespediatr.v92i3.1756

69. Leiva B F, Elberg L M, Palomino M MA. Letalidad en lactantes con cardiopatías congénitas hospitalizados por virus respiratorio sincicial. *Rev Pediatr* (2021)26–32. https://www.revistapediatria.cl/volumenes/2021/vol18num1/pdf/LETALIDAD_LACTANTES_CARDIOPATIAS_CONGENITAS_VIRUS_SINCICIAL.pdf [Accessed October 6, 2023]

70. Puchi S. A, Karlsruher R. G, Ramírez M. F. Identificación de virus respiratorios en pacientes pediátricos. *Bol Trimest Hosp Vina del Mar* (2014)61–66. http://fundacionlucassierra.cl/wp-content/files_mf/1440768010Identificación.pdf [Accessed October 6, 2023]

71. Riquelme R, Rioseco ML, Agüero Y, Ubilla D, Mechsner P, Inzunza C, Riquelme M. Infección por virus respiratorios en adultos hospitalizados en un Servicio de Medicina Interna. *Rev méd Chile* (2014) 142:696–701. doi: 10.4067/S0034-98872014000600002

72. Saldías Peñafiel F, Ortega Gutiérrez M, Fuentes López G, Elola Aránguiz JM, Uribe Monasterio J, Morales Soto A, Díaz Patiño O. Participación de los virus respiratorios en la neumonía del adulto inmunocompetente adquirida en la comunidad. *Rev méd Chile* (2016) 144:1513–1522. doi: 10.4067/S0034-98872016001200002

73. Santolaya ME, Alvarez AM, Acuña M, Avilés CL, Salgado C, Tordecilla J, Varas M, Venegas M, Villarroel M, Zubieta M, et al. Efficacy and safety of withholding antimicrobial treatment in children with cancer, fever and neutropenia, with a demonstrated viral respiratory infection: a randomized clinical trial. *Clin Microbiol Infect* (2017) 23:173–178. doi: 10.1016/j.cmi.2016.11.001

74. Valencia Sapunar M, Coke González V, González Celis A. Infecciones respiratorias no COVID. *Bol Trimest Hosp Viña del Mar* (2021)70–77. https://www.fundacionlucassierra.cl/wp-content/uploads/2021/12/INFECCIONES-RESPIRATORIAS-NO-COVID.pdf [Accessed October 6, 2023]

75. Yañez AH, Tapia LI, Benadof D, Palomino MA. Infecciones respiratorias agudas bajas: descripción de los egresos del hospital Roberto del Río en el año 2016. *Rev Pediatr* (2017)50–54. http://www.revistapediatria.cl/volumenes/2017/vol14num1/pdf/INFECCIONES_RESPIRATORIAS_AGUDAS_BAJAS_DESCRIPCION_EGRESOS_HRR_2016.pdf [Accessed October 6, 2023]

76. Zepeda T J, Vásquez Z J, Delpiano M L. Direct costs of low respiratory infection due to RSV in children under one year. *Rev Chil Pediatr* (2018) 89:462–470. doi: 10.4067/S0370-41062018005000401

77. Acuña Cordero R, Colmenares A, Gonzalez L. Impact of respiratory syncytial virus infection and palivizumab on severe acute lower respiratory infection during the first 6 months of life in a cohort of Colombian infants. *Paediatric respiratory epidemiology*. European Respiratory Society (2018) doi: 10.1183/13993003.congress-2018.pa1345

78. Bettin-Martínez A, Villareal-Camacho J, Cervantes-Acosta G, Acosta-Reyes J, Barbosa J, San Juan H. Evaluation of a loop-mediated isothermal amplification method for rapid detection of human respiratory syncytial virus in children with acute respiratory infection. *Biomedica* (2019) 39:415–426. doi: 10.7705/biomedica.v39i2.4428

79. Buendía JA, Guerrero Patiño D. Importance of respiratory syncytial virus as a predictor of hospital length of stay in bronchiolitis. *F1000Res* (2021) 10:110. doi: 10.12688/f1000research.40670.1

80. Buendía JA, Polack FP, Patiño DG. Clinical Manifestations and Outcomes of Respiratory Syncytial Virus Infection in Children Less Than Two Years in Colombia. *Indian Pediatr* (2021) 58: https://pubmed.ncbi.nlm.nih.gov/32893827/ [Accessed October 6, 2023]

81. Buendía JA, Patiño DG. Impact of the updating of clinical guidelines for RSV bronchiolitis on the use of diagnostic testing and medications in tertiary hospitals in Colombia. *Pan Afr Med J* (2022) 42:219. doi: 10.11604/pamj.2022.42.219.24876

82. Camacho-Cruz J, Briñez S, Alvarez J, Leal V, Villamizar Gómez L, Vasquez-Hoyos P. Use of the ReSVinet Scale for parents and healthcare workers in a paediatric emergency service: a prospective study. *BMJ Paediatr Open* (2021) 5:e000966. doi: 10.1136/bmjpo-2020-000966

83. Casallas Vega A, Rodriguez DR, Gómez CG, Beltrán NP, Quintero DM. Perfil del lactante con insuficiencia respiratoria que se beneficia del uso de cánula nasal de alto flujo, Bogotá - Colombia. *rhe* (2021) 32:79–90. https://revistaaisthesis.uc.cl/index.php/RHE/article/view/29419 [Accessed October 6, 2023]

84. Fernandez-Sarmiento J, Corrales SC, Obando E, Amin J, Bastidas Goyes A, Barrera Lopez PA, Bernal Ortiz N. Factors associated with severe acute respiratory infections due to Rhinovirus/Enterovirus complex in children and their comparison with those of respiratory syncytial virus. *Arch Pediatr Infect Dis* (2021) 10: doi: 10.5812/pedinfect.115548

85. García-Corzo JR, Universidad Industrial de Santander, Niederbacher-Velásquez J, González-Rugeles C, Rodríguez-Villamizar L, Machuca-Pérez M, Torres-Prieto A, Ortiz-Rodríguez G, Romero-Salazar M, Universidad Industrial de Santander, et al. Etiología y estacionalidad de las infecciones respiratorias virales en menores de cinco años en Bucaramanga, Colombia. *IATREIA* (2017) 30:117–116. doi: 10.17533/udea.iatreia.v30n2a01

86. Londono-Avendano MA, Peláez-Moreno M, López Medina E, Moreno Turriago MS, Parra Patiño B. Transmission of Respiratory Syncytial Virus genotypes in Cali, Colombia. *Influenza Other Respi Viruses* (2021) 15:521–528. doi: 10.1111/irv.12833

87. Lozano-Espinosa DA, Huertas-Quiñones VM, Rodríguez-Martínez CE. Impact of pulmonary hypertension and congenital heart disease with hemodynamic repercussion on the severity of acute respiratory infections in children under 5 years of age at a pediatric referral center in Colombia, South America. *Cardiol Young* (2020) 30:1866–1873. doi: 10.1017/S1047951120002991

88. Pedraza-Bernal AM, Rodriguez-Martinez CE, Acuña-Cordero R. Predictors of severe disease in a hospitalized population of children with acute viral lower respiratory tract infections. *J Med Virol* (2016) 88:754–759. doi: 10.1002/jmv.24394

89. Piña-Hincapie SM, Sossa-Briceño MP, Rodriguez-Martinez CE. Predictors for the prescription of albuterol in infants hospitalized for viral bronchiolitis. *Allergol Immunopathol*  (2020) 48:469–474. doi: 10.1016/j.aller.2019.10.007

90. Preciado H, Castillo MA, Díaz TF, Rodríguez JD. Bronquiolitis: Factores de riesgo en menores de dos años. Hospital de San José de Bogotá D.C. Colombia. 2013-2014. *Rev Repert Med Cir* (2015) 24:194–200. doi: 10.31260/repertmedcir.v24.n3.2015.615

91. Rodriguez-Martinez CE, Sossa-Briceño MP, Nino G. Predictors of prolonged length of hospital stay for infants with bronchiolitis. *J Investig Med* (2018) 66:986–991. doi: 10.1136/jim-2018-000708

92. Rodriguez-Martinez CE, Sossa-Briceño MP, Castro-Rodriguez JA. Direct medical costs of RSV-related bronchiolitis hospitalizations in a middle-income tropical country. *Allergol Immunopathol*  (2020) 48:56–61. doi: 10.1016/j.aller.2019.04.004

93. Rodriguez-Martinez CE, Barbosa-Ramirez J, Acuña-Cordero R. Predictors of poor outcomes of respiratory syncytial virus acute lower respiratory infections in children under 5 years of age in a middle-income tropical country based on the National Public Health Surveillance System. *Pediatr Pulmonol* (2022) 57:1188–1195. doi: 10.1002/ppul.25866

94. Ruiz-Ponce de León I, Cruz S, Rojas JP. Virus respiratorios detectados a través de biología molecular en niños hospitalizados por Infección respiratoria aguda en tiempos de SARS-CoV-2/COVID-19. *Infectio* (2021) 26:73. doi: 10.22354/in.v26i1.997

95. Suescún-Carrero SH, Vargas-Rodríguez L, González-Jiménez N, Valdivieso-Bohórquez SJ. Infección respiratoria aguda viral en dos instituciones del departamento de Boyacá, durante los años 2016-2017. *Salud UIS* (2021) 53: doi: 10.18273/saluduis.53.e:210102

96. Vela Avendaño MY. Caracterización epidemiológica de las Infecciones Respiratorias Agudas (IRA) en el área de Hospitalización Pediátrica, Clínica Antioquia- Bello, Colombia, Año 2016. (2018)70–70. http://cies.edu.ni/cedoc/digitaliza/t155/t155.pdf [Accessed October 6, 2023]

97. Villamil M, Aponte Barrios N, Restrepo SM, Rodriguez-Martinez CE. Associated Factors with Severe RSV, Adenovirus or Influenza Infections in Children Under 3 Years of Age in a Colombian Referral Hospital. American Thoracic Society International Conference Abstracts. American Thoracic Society (2019). p. A1884–A1884 doi: 10.1164/ajrccm-conference.2019.199.1_MeetingAbstracts.A1884

98. Zarur-Torralvo S, Stand-Niño I, Flórez-García V, Mendoza H, Viana-Cárdenas E. Viruses responsible for acute respiratory infections before (2016-2019) and during (2021) circulation of the SARS-CoV-2 virus in pediatric patients in a reference center at Barranquilla Colombia: A pattern analysis. *J Med Virol* (2023) 95:e28439. doi: 10.1002/jmv.28439

99. Fonseca SS. Characterization of the patients with lower respiratory tract infection who needed intensive care admission to the Intensive Care Unit at Hospital Nacional de Niños “Carlos Saenz Herrera” during the first 43 days of the respiratory season 2014. *Ital J Pediatr* (2019) 45:162. doi: 10.1186/s13052-019-0746-3

100. Gordillo Hernández A, Acosta Herrera B, Valdés Ramírez O. Etiología viral de las infecciones agudas del tracto respiratorio inferior en Cuba. *Arch méd Camagüey* (2018) 22:651–676. http://scielo.sld.cu/scielo.php?script=sci_abstract&pid=S1025-02552018000500651&lng=es&nrm=iso&tlng=es [Accessed October 6, 2023]

101. Macia Quintosa A, Marie James S, Tamayo Reus CM, Squires Murray S, Saco Rivaflecha B. Infecciones respiratorias agudas virales en pacientes menores de 5 años hospitalizados. *MEDISAN* (2021) 25:357–372. http://scielo.sld.cu/scielo.php?script=sci_abstract&pid=S1029-30192021000200357&lng=es&nrm=iso&tlng=es [Accessed October 6, 2023]

102. Martínez Muné MK, Piñón Ramos A, Acosta Herrera B, Valdés Ramírez O, Muné Jiménez M, Savón Valdés C, Arencibia García A, González Báez G, González Muñoz G, Oropesa Fernández S, et al. Introducción de nuevos ensayos de reacción en cadena de la polimerasa en tiempo real para el diagnóstico y vigilancia de virus respiratorios. *Rev Cubana Med Trop* (2018) 70:102–107. http://scielo.sld.cu/scielo.php?script=sci_abstract&pid=S0375-07602018000300011&lng=es&nrm=iso&tlng=pt [Accessed October 7, 2023]

103. Azziz-Baumgartner E, Bruno A, Daugherty M, Chico ME, Lopez A, Arriola CS, de Mora D, Ropero AM, Davis WW, McMorrow M, et al. Incidence and seasonality of respiratory viruses among medically attended children with acute respiratory infections in an Ecuador birth cohort, 2011-2014. *Influenza Other Respi Viruses* (2022) 16:24–33. doi: 10.1111/irv.12887

104. Castro Ortega JS, Atiencia Palacios JD, Juma Fernández M del C, Neira Molina VA. Estudio Transversal: Prevalencia de Infecciones por Virus Sincitial Respiratorio en Pacientes Ingresados en el Hospital de Especialidades José Carrasco Arteaga, 2017. *Rev Médica Hosp José Carrasco Arteaga* (2019) 11:132–136. doi: 10.14410/2019.11.2.ao.21

105. Suryadevara M, Fajardo FP, Aponte CC, Carrillo Aponte JL, Prado EO, Hidalgo I, Bonville CA, Torres I, Domachowske JB. Etiologies of outpatient medically attended acute respiratory infections among young Ecuadorian children prior to the start of the 2020 SARS-CoV-2 pandemic. *Influenza Other Respi Viruses* (2023) 17:e13056. doi: 10.1111/irv.13056

106. Aragón Montenegro GS. Perfil clínico y epidemiológico de los pacientes con diagnóstico de neumonía viral incluidos en la vigilancia centinela del Hospital Nacional de Niños Benjamín Bloom, de enero 2014 a diciembre 2015. (2018)81–81. http://fi-admin.bvsalud.org/document/view/8avb9 [Accessed October 7, 2023]

107. López de Orellana ML. Factores de riesgo asociados a la severidad de la Infección causada por el virus Sincitial Respiratorio (Vsr) en Menores de 2 Años del Hospital Benjamin Bloom de San Salvador, El Salvador 2014-2015. (2017)48–48. http://cies.edu.ni/cedoc/digitaliza/t947/t947.pdf [Accessed October 7, 2023]

108. Rivera Macías RE. Epidemiología y clínica de los pacientes de 1 mes a 5 años ingresados en el HNNBB con enfermedad respiratoria grave de etiología viral. Enero 2012 ­ diciembre 2013. (2016)53–53. https://fi-admin.bvsalud.org/document/view/yy33m [Accessed October 7, 2023]

109. Cannesson A, Elenga N. Community-Acquired Pneumonia Requiring Hospitalization among French Guianese Children. *Int J Pediatr* (2021) 2021:4358818. doi: 10.1155/2021/4358818

110. Gatica MA. Respiratory syncytial virus among children under age 5 years with viral acute lower respiratory infections in Guatemala. *Open Forum Infect Dis* (2014) 1:S332–S332. doi: 10.1093/ofid/ofu052.826

111. Milucky J, Pondo T, Gregory CJ, Iuliano D, Chaves SS, McCracken J, Mansour A, Zhang Y, Aleem MA, Wolff B, et al. The epidemiology and estimated etiology of pathogens detected from the upper respiratory tract of adults with severe acute respiratory infections in multiple countries, 2014-2015. *PLoS One* (2020) 15:e0240309. doi: 10.1371/journal.pone.0240309

112. Gassant P. Viral Respiratory Infections in Children in a Resource Limited Setting. *J Pediatric Infect Dis Soc* (2022) 11:S14–S14. doi: 10.1093/jpids/piac041.054

113. Ampuero JS, Lorenzana I, Gomez D, Ochoa-Diaz M, Arango A, Aguayo N, Gonzalez M, Roca Y, Ocana O, Chavez E, et al. Multiplexed detection of pathogens in respiratory illness in Latin America. *Am J Trop Med Hyg* (2019) 101:576–576.

114. Azziz-Baumgartner E, Duca LM, González R, Calvo A, Kaydos-Daniels SC, Olson N, MacNeil A, Veguilla V, Domínguez R, Vicari A, et al. Incidence of respiratory virus illness and hospitalizations in a Panama and El Salvador birth cohort, 2014-2018. *Lancet Reg Health Am* (2022) 13:None. doi: 10.1016/j.lana.2022.100304

115. Chadha M, Hirve S, Bancej C, Barr I, Baumeister E, Caetano B, Chittaganpitch M, Darmaa B, Ellis J, Fasce R, et al. Human respiratory syncytial virus and influenza seasonality patterns-Early findings from the WHO global respiratory syncytial virus surveillance. *Influenza Other Respi Viruses* (2020) 14:638–646. doi: 10.1111/irv.12726

116. Dawood FS, Jara J, Estripeaut D, Vergara O, Luciani K, Corro M, de León T, Saldaña R, Castillo Baires JM, Rauda Flores R, et al. What Is the Added Benefit of Oropharyngeal Swabs Compared to Nasal Swabs Alone for Respiratory Virus Detection in Hospitalized Children Aged <10 Years? *J Infect Dis* (2015) 212:1600–1603. doi: 10.1093/infdis/jiv265

117. Langley JM, Bianco V, Domachowske JB, Madhi SA, Stoszek SK, Zaman K, Bueso A, Ceballos A, Cousin L, D’Andrea U, et al. Incidence of Respiratory Syncytial Virus Lower Respiratory Tract Infections During the First 2 Years of Life: A Prospective Study Across Diverse Global Settings. *J Infect Dis* (2022) 226:374–385. doi: 10.1093/infdis/jiac227

118. Obando-Pacheco P, Justicia-Grande AJ, Rivero-Calle I, Rodríguez-Tenreiro C, Sly P, Ramilo O, Mejías A, Baraldi E, Papadopoulos NG, Nair H, et al. Respiratory Syncytial Virus Seasonality: A Global Overview. *J Infect Dis* (2018) 217:1356–1364. doi: 10.1093/infdis/jiy056

119. Staadegaard L, Caini S, Wangchuk S, Thapa B, de Almeida WAF, de Carvalho FC, Njouom R, Fasce RA, Bustos P, Kyncl J, et al. The Global Epidemiology of RSV in Community and Hospitalized Care: Findings From 15 Countries. *Open Forum Infect Dis* (2021) 8:ofab159. doi: 10.1093/ofid/ofab159

120. Vasquez-Hoyos P, Díaz F, Jabornisky R, Serra A, Grigolli Cesar R, Monteverde-Fernández N, Pietroboni P, Céspedes-Lesczinsky M, Wegner A, Pino P, et al. Epidemiology, patterns of care and outcomes for children with acute respiratory failure in 35 Picus from Latin America: Insights from a prospective collaborative clinical registry. *Pediatr Crit Care Med* (2021) 22:197–198. doi: 10.1097/01.pcc.0000739840.89731.bf

121. Vasquez-Hoyos P, Diaz-Rubio F, Monteverde-Fernandez N, Jaramillo-Bustamante JC, Carvajal C, Serra A, Karsies T, Rotta AT, González-Dambrauskas S, LARed Network. Reduced PICU respiratory admissions during COVID-19. *Arch Dis Child* (2020) doi: 10.1136/archdischild-2020-320469

122. Benítez-Guerra D, Piña-Flores C, Zamora-López M, Escalante-Padrón F, Lima-Rogel V, González-Ortiz AM, Guevara-Tovar M, Bernal-Silva S, Benito-Cruz B, Castillo-Martínez F, et al. Respiratory syncytial virus acute respiratory infection-associated hospitalizations in preterm Mexican infants: A cohort study. *Influenza Other Respi Viruses* (2020) 14:182–188. doi: 10.1111/irv.12708

123. Diaz J, Morales-Romero J, Pérez-Gil G, Bedolla-Barajas M, Delgado-Figueroa N, García-Román R, López-López O, Bañuelos E, Rizada-Antel C, Zenteno-Cuevas R, et al. Viral coinfection in acute respiratory infection in Mexican children treated by the emergency service: A cross-sectional study. *Ital J Pediatr* (2015) 41:33. doi: 10.1186/s13052-015-0133-7

124. Díaz-Chiguer DL, Tirado-Mendoza R, Márquez-Navarro A, Ambrosio-Hernández JR, Ruiz-Fraga I, Aguilar-Vargas RE, Lira-Martínez JM, López-Valdés JC. Detección y caracterización molecular de virus respiratorios causantes de infección respiratoria aguda en población adulta. *Gac Med Mex* (2019) 155:S16–S21. doi: 10.24875/GMM.19005138

125. Fernandes-Matano L, Monroy-Muñoz IE, Angeles-Martínez J, Sarquiz-Martinez B, Palomec-Nava ID, Pardavé-Alejandre HD, Santos Coy-Arechavaleta A, Santacruz-Tinoco CE, González-Ibarra J, González-Bonilla CR, et al. Prevalence of non-influenza respiratory viruses in acute respiratory infection cases in Mexico. *PLoS One* (2017) 12:e0176298. doi: 10.1371/journal.pone.0176298

126. González-Ortiz AM, Bernal-Silva S, Comas-García A, Vega-Morúa M, Garrocho-Rangel ME, Noyola DE. Severe Respiratory Syncytial Virus Infection in Hospitalized Children. *Arch Med Res* (2019) 50:377–383. doi: 10.1016/j.arcmed.2019.10.005

127. McCarthy S, Roldán-Aragón Y, Kapushoc H, Holley HP, Guerrero ML, Gamiño-Arroyo AE, Ramírez-Venegas A, Llamosas-Gallardo B, Galindo-Fraga A, Moreno-Espinosa S, et al. Efficacy and Safety of Nitazoxanide in Addition to Standard of Care for the Treatment of Severe Acute Respiratory Illness. *Clin Infect Dis* (2019) 69:1903–1911. doi: 10.1093/cid/ciz100

128. Quezada M, Orozco GA, Gonzalez Aguilar EE, Zapata PD, Macias AE. El virus sincicial y otros patógenos respiratorios en menores de 36 meses, en un hospital regional del centro de México. *Enfermedades Infecciosas y Microbiología* (2020) 4:75–80. http://www.amimc.org.mx/wp-content/uploads/2021/01/EIM3-2020.pdf#page=7 [Accessed October 7, 2023]

129. Robledo-Aceves M, Moreno-Peregrina M de J, Velarde-Rivera F, Ascencio-Esparza E, Preciado-Figueroa FM, Caniza MA, Escobedo-Melendez G. Risk factors for severe bronchiolitis caused by respiratory virus infections among Mexican children in an emergency department. *Medicine*  (2018) 97:e0057. doi: 10.1097/MD.0000000000010057

130. Núñez-Samudio V, Landires I. Epidemiology of viral respiratory infections in a pediatric reference hospital in Central Panama. *BMC Infect Dis* (2021) 21:43. doi: 10.1186/s12879-020-05720-1

131. Arias P, Fonseca R, Real C, Zapata L, Genes L, Escobar R, Strübing E. Infecciones respiratorias virales en pacientes hospitalizados en una unidad de cuidados intensivos neonatales. *Pediatr (Asunción)* (2019) 46:165–172. doi: 10.31698/ped.46032019003

132. Becerra M, Fiestas V, Tantaleán J, Mallma G, Alvarado M, Gutiérrez V, Huaringa M, Rojas N. Viral etiology of severe acute respiratory infections in a pediatric intensive care unit. *Rev Peru Med Exp Salud Publica* (2019) 36:231–238. doi: 10.17843/rpmesp.2019.362.4081

133. Chirinos-Saire Y, Reyna-García R, Aguilar-Huauya E, Santillán-Salas C. Respiratory viruses and clinical- epidemiological characteristics in episodes of acute respiratory infection. *Rev Peru Med Exp Salud Publica* (2021) 38:101–107. doi: 10.17843/rpmesp.2021.381.6346

134. Gómez de la Torre Pretell JC, Hueda-Zavaleta M, Cáceres-DelAguila JA, Barletta-Carrillo C, Copaja-Corzo C, Poccorpachi MDPS, Delgado MSV, Sanchez GMML, Benites-Zapata VA. Clinical Characteristics Associated with Detected Respiratory Microorganism Employing Multiplex Nested PCR in Patients with Presumptive COVID-19 but Negative Molecular Results in Lima, Peru. *Trop Med Infect Dis* (2022) 7: doi: 10.3390/tropicalmed7110340

135. Huayta Meza FT, Pacheco JMA, Poma FCG, Poma MGG, Pelaiza LEM, Crispin FVS. Sistema de seguimiento y control eProtect infecciones respiratorias. *Boletín de Malariología y Salud Ambiental* (2022) 62:518–525. http://iaes.edu.ve/iaespro/ojs/index.php/bmsa/article/view/505 [Accessed October 7, 2023]

136. Pérez-Lazo G, Silva-Caso W, Del Valle-Mendoza J, Morales-Moreno A, Ballena-López J, Soto-Febres F, Martins-Luna J, Carrillo-Ng H, Del Valle LJ, Kym S, et al. Identification of Coinfections by Viral and Bacterial Pathogens in COVID-19 Hospitalized Patients in Peru: Molecular Diagnosis and Clinical Characteristics. *Antibiotics (Basel)* (2021) 10: doi: 10.3390/antibiotics10111358

137. Ramírez-Soto MC, Ortega-Cáceres G, Garay-Uribe J. Characteristics of Respiratory Syncytial Virus versus Influenza Infection in Hospitalized Patients of Peru: A Retrospective Observational Study. *Trop Med Infect Dis* (2022) 7: doi: 10.3390/tropicalmed7100317

138. Romero C, Kasper MR, Tinoco Y, Juarez D, Silva M, Bausch DG, Williams M. Use of the filmarray respiratory panel in a resource-limited population based cohort-setting to determine the etiology of influenza-like illness. *Am J Trop Med Hyg* (2013) 89:143.

139. Romero C, Soto G, Bazán I, Casanova W, Rodriguez H, Hernández R, Tinoco Y, McCoy A. INFLUENZA AND OTHER RESPIRATORY PATHOGENS AMONG HOSPITALIZED CHILDREN IN PUBLIC HOSPITALS IN PERU. *Am J Trop Med Hyg* (2019) 101:577.

140. Tinoco Y, Romero C, Vidal F, Soto G, Silva M, Iuliano D, McCoy A. ETIOLOGY OF ACUTE RESPIRATORY INFECTIONS IN CHILDREN UNDER FIVE YEARS OLD. RESULTS FROM AN ACTIVE COMMUNITY SURVEILLANCE AND PASSIVE HOSPITAL SURVEILLANCE IN LIMA, PERU. *Am J Trop Med Hyg* (2019) 101:576.

141. Hernández Marquez GC, Oliver-Torres LS, Diaz-Diaz AM, Cotto-Aponte A, Diaz-Mejía RM, Puig-Ramos A, Puig-Ramos G, Santiago-Rodriguez C. Potassium Levels After Albuterol Continuous Therapy in Hispanic Critically Ill Children with Respiratory Distress. (2019)A1877–A1877. doi: 10.1164/ajrccm-conference.2019.199.1_MeetingAbstracts.A1877

142. Paz-Bailey G, Adams L, Munoz J, Lozier M, Olsen S, Blanton L, Waterman S, Alvarado L. Trends in influenza and other respiratory viruses in Southern Puerto Rico, 2012-2017. *Am J Trop Med Hyg* (2018) 99:1–718. doi: 10.4269/ajtmh.abstract2018

143. Quandelacy TM, Adams LE, Munoz J, Santiago GA, Kada S, Johansson MA, Alvarado LI, Rivera-Amill V, Paz-Bailey G. Reduced spread of influenza and other respiratory viral infections during the COVID-19 pandemic in southern Puerto Rico. *PLoS One* (2022) 17:e0266095. doi: 10.1371/journal.pone.0266095

144. Rivera-Sepulveda A, Garcia-Rivera EJ. Epidemiology of bronchiolitis: a description of emergency department visits and hospitalizations in Puerto Rico, 2010-2014. *Trop Med Health* (2017) 45:24. doi: 10.1186/s41182-017-0064-7

145. Rodriguez-Santiago RM, Rodriguez-Gonzalez R, Torres-Rivera K, Rivera-Amill V, Alvarado-Domenech LI, Adams L. Respiratory virus infections from the sentinel enhanced dengue surveillance system, 2016-2019. *Am J Trop Med Hyg* (2020) 103:433. doi: 10.4269/ajtmh.abstract2020

146. Grunberg M, Sno R, Adhin MR. Epidemiology of respiratory viruses in patients with severe acute respiratory infections and influenza-like illness in Suriname. *Influenza Other Respi Viruses* (2021) 15:72–80. doi: 10.1111/irv.12791

147. Juliana AE, Tang M-J, Kemps L, Noort AC, Hermelijn S, Plötz FB, Zonneveld R, Wilschut JC. Viral causes of severe acute respiratory infection in hospitalized children and association with outcomes: A two-year prospective surveillance study in Suriname. *PLoS One* (2021) 16:e0247000. doi: 10.1371/journal.pone.0247000

148. Machado K, Pérez W, Pírez C, Stoll M. Hospitalizaciones por IRAB de probable etiología viral en niños durante el año 2012: estrategia asistencial. *Arch Pediatr Urug* (2016) 87:5–11. http://www.scielo.edu.uy/scielo.php?script=sci_abstract&pid=S1688-12492016000100002&lng=es&nrm=iso&tlng=es [Accessed October 7, 2023]

149. Machado K, Notejane M, Mello M, Pírez MC, Giachetto G, Pérez W. Infecciones respiratorias agudas bajas en niños menores de 2 años. Hospitalizaciones durante el invierno del año 2014. *An Fac Med* (2018) 5:45–55. doi: 10.25184/anfamed2018v5n1a9

150. Morosini F, Notejane M, Machado K, Páez M, Rompani E, Taboada R, Mariño H, Castelli X, Pírez C. Ventilación no invasiva y oxigenoterapia de alto flujo en niños en salas de cuidados moderados. Experiencia en la Unidad de Cuidados Respiratorios Especiales Agudos del Hospital Pediátrico del CHPR durante 2013-2016. *Arch Pediatr Urug* (2018) 89:78–85. doi: 10.31134/ap.89.2.2

151. Pinchak C, García L, Peluffo G, Vázquez M, Halty M, Chamorro F, Mogni A, Iglesias S, Noria A, Ferratti M, et al. Experiencia en la utilización de cánula nasal de alto flujo en niños con infecciones respiratorias agudas hospitalizados en un sector de internación. *Arch Pediatr Urug* (2019) 90:257–269. doi: 10.31134/ap.90.5.2

152. Sánchez M, Carugati MJ, Pinto S, Etcheverry G, Pírez C. Hospitalizaciones pediátricas por infecciones respiratorias agudas durante la pandemia por SARS-CoV-2. Hospital Británico, Uruguay. *Arch Pediatr Urug* (2021) 92: doi: 10.31134/ap.92.1.4

153. Astudillo Molina CM, Palomino Montenegro MA. Frecuencia de niños con síndrome de Down hospitalizados por virus respiratorio sincicial en el Hospital Roberto del Rio en los años 2014-2015^ies. *Rev Pediatr* (2018) 15:2–6.

154. Billard MN, van de Ven PM, Baraldi B, Kragten-Tabatabaie L, Bont LJ, Wildenbeest JG. International changes in respiratory syncytial virus (RSV) epidemiology during the COVID-19 pandemic: Association with school closures. *Influenza Other Respi Viruses* (2022) 16:926–936. doi: 10.1111/irv.12998

155. Silva DGBP da, Almeida FJ, Arnoni MV, Sáfadi MAP, Mimica MJ, Jarovsky D, Rossetti GP de A, Magalhães M, Departamento de MicrobiologiaOliveira DBL de, Departamento de MicrobiologiaThomazelli LM, et al. First report of two consecutive respiratory syncytial virus outbreaks by the novel genotypes ON-1 and NA-2 in a neonatal intensive care unit^ienPrimeiro relato de dois surtos consecutivos de vírus sincicial respiratório pelos novos genótipos ON-1 e NA-2 e. *J Pediatr*  (2020) 96:233–239.

156. CONITEC. Palivizumabe para a prevenção da infecção pelo vírus sincicial respiratório. (2012)

157. Buendia JA, Lindarte EF, Polack FP. TLR4 Gene Polymorphisms Interaction With Ascaris Infection in Severe RSV Bronchiolitis. *Frontiers in Pediatrics* (2022) 10:876882. doi: 10.3389/fped.2022.876882

158. Caballero MT, Grigaites SD, Niveyro PXD, Esperante S, Bianchi AM, Nuno A, Valle S, Afarian G, Ferretti AJP, Baglivo SJ, et al. Uncovering Causes of Childhood Death Using the Minimally Invasive Autopsy at the Community Level in an Urban Vulnerable Setting of Argentina: A Population-Based Study. *Clin Infect Dis* (2021) 73:S435–S441. doi: 10.1093/cid/ciab838

159. Cardoso AM, Resende PC, Paixao ES, Tavares FG, Farias YN, Barreto CTG, Pantoja LN, Ferreira FL, Martins AL, Lima ÂB, et al. Investigation of an outbreak of acute respiratory disease in an indigenous village in Brazil: Contribution of Influenza A(H1N1)pdm09 and human respiratory syncytial viruses. *PLoS One* (2019) 14:e0218925. doi: 10.1371/journal.pone.0218925

160. Cintra MACT, Santos MS, Correia MG, Tura BR. Cost effectiveness analysis of respiratory syncytial virus infection prophylaxis in children with congenital heart disease. *Value Health* (2014) 17:A176. doi: 10.1016/j.jval.2014.03.1028

161. Ferreira HLDS, Costa KLP, Cariolano MS, Oliveira GS, Felipe KKP, Silva ESA, Alves MS, Maramaldo CEC, de Sousa EM, Rego JS, et al. High incidence of rhinovirus infection in children with community-acquired pneumonia from a city in the Brazilian pre-Amazon region. *J Med Virol* (2019) 91:1751–1758. doi: 10.1002/jmv.25524

162. Fernandes RRA. Custo-efetividade do uso do palivizumabe na prevenção de internações por doença respiratória grave em crianças de alto risco infectadas pelo vírus sincicial respiratório na perspectiva do Sistema Único de Saúde. *Pós-Graduação em Saúde Coletiva* (2014) Mestrado: https://docs.bvsalud.org/biblioref/2018/09/914906/dissertacao_ufrg_2014.pdf

163. Galvis C, Colmenares A, Cabrales L, Ibata L, Marulanda J, Ovalle O, Puello D, Rojas C, Africano M, Ballesteros A, et al. Impact of immunoprophylaxis with palivizumab on respiratory syncytial virus infection in preterm infants less than 35 weeks in Colombian hospitals. *Pediatr Pulmonol* (2022) 57:2420–2427. doi: 10.1002/ppul.26051

164. García Corzo JR, Niederbacher Velasquez J, González Rugéles CI, Rodríguez Villamizar LA, Machuca Pérez M, Torres Prieto A, Ortiz Rodríguez GC, Romero Salazar M. Etiología viral de infección respiratoria aguda en niños menores de 5 años en las provincias Comunera y García Rovira de Santander^iesEtiology of acute respiratory infection in children under 5 years in the provinces Comunera and García Rovira of Santande. *Rev Univ Ind Santander, Salud* (2016) 48:240–245.

165. Gaytán-Morales JF, Castorena-Villa I, Cortés-Flores DC, Avilés-Robles MJ, Sánchez-Huerta JL, Ortiz-Navarrete V, Olvera-Gómez I, López-Martínez B, Parra-Ortega I. Respiratory viral infections in pediatric patients with hematopoietic stem cell transplantation. *Bol Med Hosp Infant Mex* (2021) 78:191–199. doi: 10.24875/bmhim.20000126

166. Gentile A, Lucion MF, Del Valle Juarez M, Areso MS, Bakir J, Viegas M, Mistchenko A. Burden of respiratory syncytial virus disease and mortality risk factors in Argentina: 18 years of active surveillance in a children’s hospital. *Pediatr Infect Dis J* (2019) 38:589–594. doi: 10.1097/INF.0000000000002271

167. Sanchez LEG, Wentzel G, Ramirez MNM. Bronquiolitis por rinovirus en menores de 1 año y riesgo de sibilancias recurrentes y asma. *Pediatr (Asunción)* (2021) 48:113–119. doi: 10.31698/ped.48022021005

168. Gonzalez-Dambrauskas S, Diaz F, Monteverde-Fernandez N, Carvajal C, Serra JA, Wegner A, Cruces P, Cespedes M, Nunez-Sanchez MJ, Guachichulca R, et al. Wide variability of intensive care management for infants with bronchiolitis in the Latin-American collaborative network (LARED). *Pediatr Crit Care Med* (2018) 19:230. http://ovidsp.ovid.com/ovidweb.cgi?T=JS&PAGE=reference&D=emed19&NEWS=N&AN=623815260

169. Jiménez Herrera LG, Collado Herrera M, Gálvez González AM. Costo-efectividad incremental del Palivizumab en prematuros en la Seguridad Social de Costa Rica^iesIncremental cost- effectiveness of palivizumab in premature at Costa Rican Social Security^ienCusto-efetividade incremental do palivizumabe em prematuros d. *Horiz sanitario (en linea)* (2020) 19:195–207.

170. Jurado Hernandez VH, Sanchez-Casillas JL, Vo P, Laws AJ, Gooch KL, Kendall RM. The short-term economic impact of childhood preventive health programs in Mexico. *Value Health* (2014) 17:A172–A173. doi: 10.1016/j.jval.2014.03.1006

171. Luisi F, Roza CA, Silveira VD, Machado CC, Rosa KM da, Pitrez PM, Jones MH, Stein RT, Leitão LA de A, Comaru T, et al. A azitromicina administrada para bronquiolite aguda pode ter um efeito de proteção na sibilância recorrente^iptAzithromycin administered for acute bronchiolitis may have a protective effect on subsequent wheezing^ien. *J Bras Pneumol* (2020) 46:

172. Majer I, Pichardo-Pina CA, Sanchez-Casillas JL, Schmidt R, Vo P. Cost-effectiveness of palivizumab in premature infants and children with chronic lung disease in Mexico. *Value Health* (2015) 18:A834. http://ovidsp.ovid.com/ovidweb.cgi?T=JS&PAGE=reference&D=emed16&NEWS=N&AN=72101101

173. Meyer VMC, Siqueira MM, Costa P, Caetano BC, Oliveira Lopes JC, Folescu TW, Motta FDC. Clinical impact of respiratory virus in pulmonary exacerbations of children with Cystic Fibrosis. *PLoS One* (2020) 15:e0240452. doi: 10.1371/journal.pone.0240452

174. Moubarak MJ, Bonville CA, Domachowske JB, Suryadevara M, Salazar-Gomez GM, Borbor-Cordova MJ, Sippy R, Cueva-Aponte C, Ortiz-Prado E, Hidalgo I, et al. 2018-2019 seasonal epidemiology of infections caused by influenza viruses and rsv in ecuadorean children less than 5 years of age residing at opposite extremes of elevation. *Open Forum Infectious Diseases* (2019) 6:S799. doi: 10.1093/ofid/ofz360.2005

175. Mould-Quevedo JF, Contreras-Hernandez I, Martinez-Valverde S, Villasis-Keever MA, Granados-Garcia VM, Salinas-Escudero G, Munoz-Hernandez O. Direct medical costs of treating Mexican children under 2 years of age with respiratory syncytial virus. *Bol Med Hosp Infant Mex* (2012) 69:111–115. http://ovidsp.ovid.com/ovidweb.cgi?T=JS&PAGE=reference&D=emed13&NEWS=N&AN=365462123

176. Mykietiuk A, Obed M, Cooke B, Alzogaray MF, Romandetta A, Pablo Balbuena J, Galeano L, Chaparro G, Baumeister E. Respiratory syncytial virus in elderly adults. *Open Forum Infectious Diseases* (2019) 6:S984–S985. doi: 10.1093/ofid/ofz360.2464

177. Nader PDJ, Nader SS, Jung C, Harff A. Respiratory infections in children up two years of age on prophylaxis with palivizumab, three years of experience. *Eur J Pediatr* (2016) 175:1780–1781. doi: 10.1007/s00431-016-2785-8

178. de Oliveira M, Pereira C, Costa MG. PIH15 EXTERNAL VALIDATION OF THE EX-ANTE BUDGET IMPACT ANALYSIS FOR PALIVIZUMAB. *Value Health* (2019) 22:S630. doi: 10.1016/j.jval.2019.09.1197

179. Paladio Hernandez JA, Lagunas-Quiroz JI, Matias-Juan NA. PIH26 COST EFFECTIVENESS OF PALIVIZUMAB FOR RESPIRATORY SYNCYTIAL VIRUS PROPHYLAXIS AMONG EXTREME PRETERM INFANTS (29 WEEKS OF GESTATIONAL AGE) AT THE SOCIAL SECURITY MEXICAN INSTITUTE (IMSS). *Value Health* (2020) 23:S156. doi: 10.1016/j.jval.2020.04.421

180. Pineros JG, de la Hoz-Valle J, Galvis C, Celis A, Ovalle O, Sandoval CC, Orrego J, Vides S, Rojas I, Bustamante H, et al. Effectiveness of palivizumab immunoprophylaxis in infants with respiratory syncytial virus disease in Colombia. *J Infect Dev Ctries* (2021) 15:1708–1713. doi: 10.3855/JIDC.12561

181. de Souza ABC, da Silva PM, Silva GF, Simioni AJ, Zanetti LP, Moreno JRD, de Oliveira LCS, Colturato I, Franceschi FLD, dos Santos ACF, et al. RSV infection in HSCT recipients: Experience from one single center. *Bone Marrow Transplant* (2019) 54:410–411.

182. Romero-Espinoza JA, Moreno-Valencia Y, Coronel-Tellez RH, Castillejos-Lopez M, Hernandez A, Dominguez A, Miliar-Garcia A, Barbachano-Guerrero A, Perez-Padilla R, Alejandre-Garcia A, et al. Virome and bacteriome characterization of children with pneumonia and asthma in Mexico City during winter seasons 2014 and 2015. *PLoS One* (2018) 13:e0192878. doi: 10.1371/journal.pone.0192878

183. Rosselli D, Rueda JD, Ruiz JG. Cost-effectiveness of prophylaxis of respiratory syncytial virus infection (RSV) with palivizumab in preterm infants in colombia. *Value Health* (2013) 16:A75. doi: 10.1016/j.jval.2013.03.337

184. Rueda J-D, Rosselli D, Ruiz-Pelaez JG. Cost-Effectiveness of Respiratory Syncytial Virus Infection (RSV) Prophylaxis with Palivizumab in Preterm Infants in Colombia. *Coyuntura Economica: Investigacion Economica y Social* (2013) 43:137–151.

185. Seliem W, Sultan AM. Heliox delivered by high flow nasal cannula improves oxygenation in infants with respiratory syncytial virus acute bronchiolitis^ienHeliox administrado por cânula nasal de alto fluxo melhora a oxigenação em crianças com bronquiolite aguda por vírus sincic. *J Pediatr*  (2018) 94:56–61.

186. Sinisterra D, Buendia JA. Cost illness of RSV infection in Colombia. *Value Health* (2017) 20:A923–A924. doi: 10.1016/j.jval.2017.08.2886

187. Solis-Esquilin EA, Flores-Arroyo P, Quiles-Sanchez V, Pagan-Albelo C, Puig-Ramos A, Puig-Ramos G, Esquilin I. Profile of Hispanic Patients admitted to a Pediatric Intensive Care Unit in a community hospital due to influenza. *Pediatrics* (2020) 146:169–170. doi: 10.1542/peds.146.1_MeetingAbstract.169-a

188. Tomashek KM, Lorenzi O, Gonzalez G, Perez-Padilla J, Munoz J, Hunsperger E, Rivera A, Oberste S, Nix WA, Henderson E, et al. First year findings from an acute febrile illness surveillance study in Puerto Rico. *Am J Trop Med Hyg* (2013) 89:171–172. http://ovidsp.ovid.com/ovidweb.cgi?T=JS&PAGE=reference&D=emed14&NEWS=N&AN=71312392

189. Tobolowsky FA, Zambrano L, Sharp T, Beckham JD, Alvarado L, Paz-Bailey G. Association of body mass index with rates of hospitalization in patients with respiratory viral infections?Puerto Rico, 2012-2018. *Open Forum Infectious Diseases* (2019) 6:S986–S987. doi: 10.1093/ofid/ofz360.2469

190. Villamil JPS, Polack FP, Buendía JA. Disability-adjusted life years for respiratory syncytial virus in children under 2 years. *BMC Public Health* (2020) 20:1–5. doi: 10.1186/s12889-020-09796-x
